# Characterization and trajectories of hematological parameters prior to severe COVID-19 based on a large-scale prospective health checkup cohort in western China: a longitudinal study of 13-year follow-up

**DOI:** 10.1186/s12916-024-03326-x

**Published:** 2024-03-07

**Authors:** Yifei Lin, Yong Yang, Nanyan Xiang, Le Wang, Tao Zheng, Xuejun Zhuo, Rui Shi, Xiaoyi Su, Yan Liu, Ga Liao, Liang Du, Jin Huang

**Affiliations:** 1 Department of Urology, Innovation Institute for Integration of Medicine and Engineering, West China Hospital, Sichuan University, Chengdu, Sichuan People’s Republic of China; 2 Health Management Center, General Practice Medical Center, Innovation Institute for Integration of Medicine and Engineering, West China Hospital, Sichuan University, Chengdu, Sichuan People’s Republic of China; 3 Department of Urology, Innovation Institute for Integration of Medicine and Engineering, Frontiers Science Center for Disease-Related Molecular Network, West China Hospital, Sichuan University, Chengdu, Sichuan People’s Republic of China; 4 Department of Urology, Innovation Institute for Integration of Medicine and Engineering, Frontiers Science Center for Disease-Related Molecular Network, Key Laboratory of Bio-Resource and Eco-Environment of Ministry of Education, College of Life Sciences, West China Hospital, Sichuan University, Chengdu, Sichuan People’s Republic of China; 5 Engineering Research Center of Medical Information Technology, Ministry of Education, West China Hospital of Sichuan University, Chengdu, Sichuan People’s Republic of China; 6 Department of Urology, Innovation Institute for Integration of Medicine and Engineering, Chinese Evidence-Based Medicine Center, West China Hospital, West China Hospital, Sichuan University, Chengdu, Sichuan People’s Republic of China; 7 Department of Neurosurgery, Innovation Institute for Integration of Medicine and Engineering, Ministry of Education, West China Hospital of Sichuan University, Chengdu, Sichuan People’s Republic of China; 8 State Key Laboratory of Oral Diseases, National Clinical Research Center for Oral Diseases, West China Hospital of Stomatology, Sichuan University, Chengdu, Sichuan People’s Republic of China

**Keywords:** Health checkup, Prospective cohort, COVID-19, Proactive health, Long term

## Abstract

**Background:**

The relaxation of the “zero-COVID” policy on Dec. 7, 2022, in China posed a major public health threat recently. Complete blood count test was discovered to have complicated relationships with COVID-19 after the infection, while very few studies could track long-term monitoring of the health status and identify the characterization of hematological parameters prior to COVID-19.

**Methods:**

Based on a 13-year longitudinal prospective health checkup cohort of ~ 480,000 participants in West China Hospital, the largest medical center in western China, we documented 998 participants with a laboratory-confirmed diagnosis of COVID-19 during the 1 month after the policy. We performed a time-to-event analysis to explore the associations of severe COVID-19 patients diagnosed, with 34 different hematological parameters at the baseline level prior to COVID-19, including the whole and the subtypes of white and red blood cells.

**Results:**

A total of 998 participants with a positive SARS-CoV-2 test were documented in the cohort, 42 of which were severe cases. For white blood cell-related parameters, a higher level of basophil percentage (HR = 6.164, 95% CI = 2.066–18.393, *P* = 0.001) and monocyte percentage (HR = 1.283, 95% CI = 1.046–1.573, *P* = 0.017) were found associated with the severe COVID-19. For lymphocyte-related parameters, a lower level of lymphocyte count (HR = 0.571, 95% CI = 0.341–0.955, *P* = 0.033), and a higher CD4/CD8 ratio (HR = 2.473, 95% CI = 1.009–6.059, *P* = 0.048) were found related to the risk of severe COVID-19. We also observed that abnormality of red cell distribution width (RDW), mean corpuscular hemoglobin concentration (MCHC), and hemoglobin might also be involved in the development of severe COVID-19. The different trajectory patterns of RDW-SD and white blood cell count, including lymphocyte and neutrophil, prior to the infection were also discovered to have significant associations with the risk of severe COVID-19 (all *P* < 0.05).

**Conclusions:**

Our findings might help decision-makers and clinicians to classify different risk groups of population due to outbreaks including COVID-19. They could not only optimize the allocation of medical resources, but also help them be more proactive instead of reactive to long COVID-19 or even other outbreaks in the future.

**Supplementary Information:**

The online version contains supplementary material available at 10.1186/s12916-024-03326-x.

## Background

Throughout 2023, the world continued to face the challenges posed by the prolonged COVID-19 pandemic, which has now spanned 3 years. According to the World Health Organization (WHO) Coronavirus (COVID-19) Dashboard, as of February 17, 2023, there have been more than 756 million confirmed cases of COVID-19, including more than 6 million deaths globally [1]. Even though the severity and mortality of COVID-19 seem to be declined due to the mutation of variants, the large population base and numerous elder people with complex comorbidities would still pose higher challenges for the government, healthcare systems, and researchers [2].

As one of the most common laboratory tests, a complete blood count test was discovered to have complicated relationships with COVID-19 by numerous researchers. Several leukocyte counts, including lymphocyte, monocyte, and neutrophil, were found altered after diagnosis of COVID-19 [3]. In particular, they play an important role in the hyperinflammatory state and cytokine storm, a lethal inflammatory situation in COVID-19 patients [4]. Furthermore, hemoglobin concentration was also found to decrease with disease severity for the increased levels of glycolytic intermediates and oxidation and fragmentation of membrane proteins in red blood cells [5].

However, most of the studies were limited due to cross-sectional or hospital-based design after the infection [6, 7]. Recently, increasing evidence tried to prove that the baseline health status prior to COVID-19 infection might also determine the severity and prognosis [8], whereas very few could track long-term monitoring of the health status prior to the infection.

Since the Chinese government lifted the “zero-COVID” restriction on Dec. 7, 2022, the Omicron variant of COVID-19 has spread rapidly across the country and the outbreak was predicted to peak in late December [9, 10]. On Dec. 21st., the deputy director of the Chinese Center for Disease Control and Prevention (China CDC) claimed that, in large regions, namely Sichuan province which is also the largest economy in western China, more than 50% of residents had been infected [10]. Consistently ranked as the top one research hospital in China, West China Hospital (WCH) stands as the largest and most advanced medical center in Sichuan province and Western China [11]. Featuring three distinct medical subcenters and five health management checkup subcenters, WCH has played a pivotal role in responding to various outbreaks [12]. More importantly, to better surveil the health status of the population and help respond to epidemic disease, WCH started to collect checkup information in 2010 and further established a Big Data Platform to integrate all the electrical medical records [13].

Therefore, based on the longitudinal prospective checkup cohort of ~ 480,000 participants in Western China, we investigated the associations of baseline hematological parameters prior to COVID-19 including the whole and subtypes of red blood cell white blood cell and other related parameters with the risk of developing severe COVID-19. Among the individuals with COVID-19, we further characterized the potential trajectories of certain hematological parameters prior to COVID-19 and examined the possible associations with COVID-19 severity. We hope our findings could help decision-makers and clinicians not only classify different risk groups to optimize the allocation of medical resources but also help them be more proactive [14] instead of reactive to long COVID-19 or even other outbreaks in the future [15].

## Methods

### Design, setting, and participants

This study reports on the initial data release of the WHALE cohort (West China Hospital Alliance Longitudinal Epidemiology Wellness Cohort). Established as a comprehensive longitudinal initiative, the WHALE cohort represents a large-scale prospective cohort of health checkup participants conducted in West China Hospital, Sichuan University, from 2010 to 2023 (Chinese Clinical Trial Registry [http://www.chictr.org.cn/index.aspx], identifier: ChiCTR2200066950). A total of 478,898 participants have undergone periodic health checkups at the Health Management Center of West China Hospital, which consists of one headquarters and four subcenters including Wuhou, Wenjiang, Tianfu, and Shangjin [16]. During the following 1 month after the relaxation of the “zero COVID” policy in China since Dec. 7, 2022, we documented 998 participants with a laboratory-confirmed diagnosis of COVID-19 (positive SARS-CoV-2 nucleic acid test). All participants have completed at least one admission with general health checkup items including vital signs, body measurement (height, weight, body mass index (BMI), blood pressure, etc.), laboratory tests (blood routine test, urine routine test, etc.), and so on. Using a unique ID, one participant’s electronic health records can be obtained from the Big Data Platform of the West China Hospital, which include three independent medical subcenters (University Campus, Wenjiang Hospital, and Shangjin Hospital) [13]. This study was approved by the Ethics Committee of West China Hospital, Sichuan University, with a waiver of informed consent (No. 2023–245). Results are reported in accordance with the Strengthening the Reporting of Observational Studies in Epidemiology (STROBE) reporting guideline [17].

### Exposure assessments

After fasting overnight for 10–12 h, the peripheral blood samples were collected in the morning by experienced nurses at the Health Management Centers of West China Hospital. Then, the blood cell tests were all performed at the clinical laboratory of the West China Hospital following standard procedures [18]. The hematological parameters available investigated in the current study include white blood cell-related parameters (white blood cell count (WBC), neutrophil count (NeuC), neutrophil percentage (Neu%), lymphocyte count (LymC), lymphocyte percentage (Lym%), basophil count (BasC), basophil percentage (Bas%), eosinophil count (EosC), eosinophil percentage (Eos%), monocyte count (MonC), monocyte percentage (Mon%)), red blood cell-related parameters (red blood cell count (RBC), red cell distribution width (SD) (RDW-SD), red cell distribution width (CV) (RDW-CV), hematocrit (HCT), mean corpuscular hemoglobin concentration (MCHC), mean corpuscular hemoglobin (MCH), mean corpuscular volume (MCV), hemoglobin (Hb), T cell markers (CD3 count/percentage, CD4/count percentage, CD8 count/percentage and CD4/CD8 ratio), and blood platelet count (PLT).

Inflammation signifies the immune system’s response to harmful stimuli. Thus, in addition to the aforementioned parameters, we investigated five ratios between cell population frequencies to enhance our understanding of immune and hematological status in our study population [19, 20]. These ratios include monocyte-to-lymphocyte ratio (MoLR), neutrophil-to-lymphocyte ratio (NLR), eosinophil-to-lymphocyte ratio (ELR), basophil-to-lymphocyte ratio (BLR), and platelet-to-lymphocyte ratio (PLR).

Data for hematology analytes including red blood cell count, white blood cell count, platelet (PLT), and hemoglobin (Hb) were determined using the XE-2100 and XE-5000 systems (Sysmex, Kobe, Japan). The levels of T lymphocytes (CD3, CD4, and CD8) were detected by flow cytometry (six-color flow cytometry, BD Company, USA); equation* K* value of erythrocyte sedimentation rate (ESR-K) and erythrocyte sedimentation rate (ESR) were detected using Alifax Test 1 (ALIFAX Company, Italy).

### Ascertainment of outcome

Although regular nucleic acid (RT-PCR) testing for COVID-19 is not required anymore after the relaxation of the “zero COVID” policy in China since Dec. 7, 2022, still numerous people would come to hospitals to have COVID-19 nucleic acid tests and even in need intensive care. Thus, we documented participants from the prospective cohort with a laboratory-confirmed diagnosis of COVID-19 (positive SARS-CoV-2 nucleic acid test) during the following 1 month (Dec. 7, 2022 to Jan 6, 2023), in West China Hospital, Sichuan University, in the current study.

According to the in-hospital care received [21], all the participants were categorized into two groups: (1) patients with severe COVID-19 required intensive care (including mechanical ventilatory [22] or high-flow oxygen [23]) or signed a critical illness notice; (2) patients with no need for above interventions were recognized as non-severe COVID-19 cases. Further, we classified the non-severe COVID-19 cases into two subgroups for sensitivity analysis, namely mild COVID-19 which required ambulatory care and moderate COVID-19 which required non-intensive hospitalized treatment.

### Ascertainment of covariates

As age, gender (male, female), BMI, smoking status (never smokers, former smokers, often smokers, and occasionally smokers), drinking habits (lifetime abstainers, former drinkers, often drinkers, and occasionally drinkers), hypertension (with hypertension, no hypertension), and diabetes (with diabetes, no diabetes) were identified as considerable risk factors for COVID-19, we add these indicators as covariates sequentially in our main and sensitivity analysis [24, 25]. All the covariates information was collected using a standardized questionnaire during the health checkup.

### Statistical analysis

In this study, the baseline characteristics were described according to the COVID-19 severity groups. Continuous variables are presented as the median and interquartile range (IQR, 25–75th percentile), and categorical variables are reported as numbers (*n*) and percentages (%). To estimate differences across COVID-19 severity groups, continuous variables were statistically inferred by the Mann–Whitney *U* test or Kruskal–Wallis *H* test, and categorical variables were tested by Fisher’s exact test.

To explore the association between baseline hematological parameters and the severity of COVID-19, we first identified the time-to-event in days from the date of cohort admission to the date of COVID-19 diagnosis. Then, we modeled the association between baseline hematological parameters and the risk of severe COVID-19 using unadjusted Cox regression proportional hazards analysis and 3 sequential models of adjusted Cox proportional hazards analysis. Model 1 was adjusted for baseline age, gender, and BMI. Model 2 also included smoking status and drinking habits. Model 3 also included hypertension and diabetes. The hazard ratio (HR) and 95% confidence interval (CI) were used to determine the strength of the effects. Considering the small value of BasC, MoLR, and BLR, we rescaled it (multiply by 10) for all the models to make the results reasonable, while in the descriptive analysis, we kept the original value of this parameter.

For sensitivity analysis, we then adopted binary and ordinal logistic regression using the same model (unadjusted model, models 1, 2, and 3) to validate the association between hematological parameters and the severity of COVID-19. In the ordinal logistic analysis, 998 COVID-19 patients were divided into three groups (mild, moderate, and severe) according to the severity of the COVID-19. The odds ratio (OR) and 95% confidence interval were used to report the results.

To identify potential trajectory patterns of hematological parameters before COVID-19, we extracted all follow-up data of the studied patients from the cohort while excluding those with less than twice blood tests. Parameters identified significantly associated with COVID-19 in the foregoing analysis, as well as those reported as related factors of COVID-19, were included [26–28] (detailed methods for construction of trajectory can be found in Additional file 1: Supplemental Method [29–35]).

Finally, we investigated the relationship of the severity of COVID-19 with different trajectory pattern groups of hematological parameters by performing the Cox proportional hazards analysis, using the previous 4 models. Binary logistic regression analysis was also conducted to validate the results from Cox analysis. For the significant parameters obtained in the Cox and logistic analysis for trajectories, Kaplan–Meier analysis was then employed to further examine the association between severe COVID-19 and trajectories of specific hematological parameters. The trajectory analysis was performed using the R package “lcmm” (version 2.0.0) and all analyses were conducted using R software (version 4.0.3, R package “survival” “TableOne”) [28]. A *P* value of < 0.05 was considered significant for all analyses.

## Results

### Baseline characteristics

**Fig. 1:**
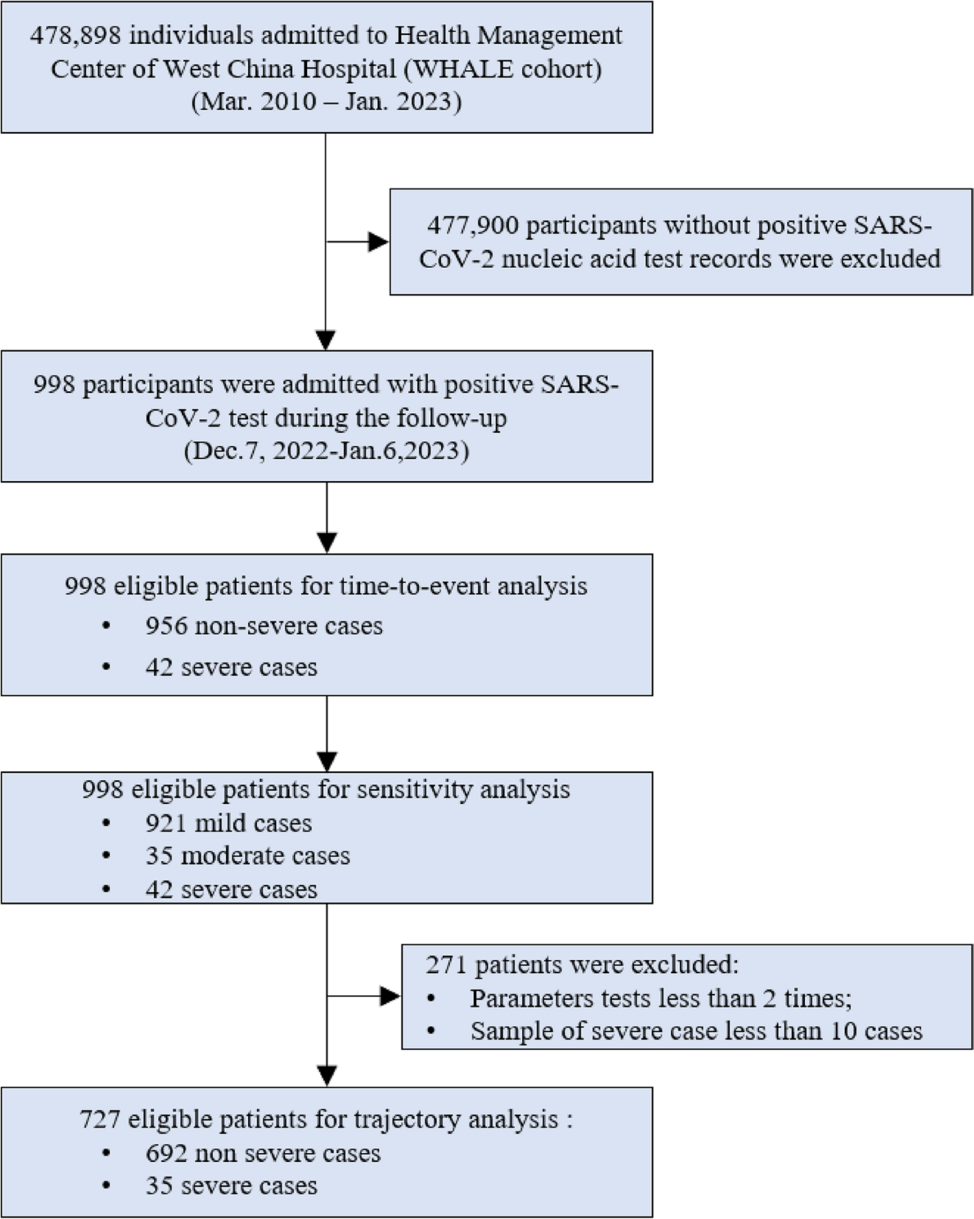
Flow diagram of the study

Among the 478,898 participants admitted to the Health Management Center of West China Hospital from 2010 to 2023, we documented 998 participants with a positive SARS-CoV-2 test over 13 years of follow-up (Fig. 1 shows the flow diagram of the study). Among those participants, 4.2% (*n* = 42) were severe cases and 95.8% (*n* = 956) were non-severe cases.

Of the 998 patients, 43.9% (*n* = 438) were women and 93.0% (*n* = 928) were less than 65 years old and the median age was 37 years old (IQR, 28–48 years). 61.6% (*n* = 615) of patients reported never drinking and 77.3% (*n* = 772) of participants reported never smoking. 11.2% (*n* = 112) of patients had hypertension, and 4.5% (*n* = 45) had diabetes. The baseline results of all 34 hematological parameter-related examinations can be found in Table 1.

**Table 1:** Baseline characteristics of patients with COVID-19

	**Illness severity**			
	**Non-severe (*n* = 956)**	**Severe (*n* = 42)**	**All (*n* = 998)**	***P* value**
**Variables**				
Age (years)*				< 0.001
< **80**	950 (99.4%)	36 (85.7%)	986	
≥ **80**	6 (0.6%)	6 (14.3%)	12	
**Sex**				< 0.001
**Male**	525 (54.9%)	35 (83.3%)	560	
**Female**	431 (45.1%)	7 (16.7%)	438	
**Smoking status**				0.135
**Never**	744 (77.8%)	28 (66.7%)	772	
**Former**	17 (1.8%)	1 (2.4%)	18	
**Often**	147 (15.4%)	8 (19%)	155	
**Occasionally**	48 (5%)	5 (11.9%)	53	
**Drinking status**				0.307
**Never**	589 (61.6%)	26 (61.9%)	615	
**Former**	3 (0.3%)	1 (2.4%)	4	
**Often**	66 (6.9%)	2 (4.8%)	68	
**Occasionally**	298 (31.2%)	13 (31%)	311	
**Hypertension**				< 0.001
**No hypertension**	864 (90.4%)	22 (52.4%)	886	
**With hypertension**	92 (9.6%)	20 (47.6%)	112	
**Diabetes**				< 0.001
**No diabetes**	919 (96.1%)	34 (81%)	953	
**With diabetes**	37 (3.9%)	8 (19%)	45	
**Continuous variables (median (IQR))**				
**Red blood cell count (10**^**12**^**/L)**	4.89 (4.55–5.23)	4.73 (4.47–5.12)	4.87 (4.55–5.23)	0.060
**Red cell distribution width (SD) (fL)**	43.55 (41.40–45.60)	46.95 (45.10–48.65)	43.60 (41.50–45.80)	< 0.001
**Red cell distribution width (CV) (%)**	13.10 (12.60–13.60)	13.75 (13.30–14.30)	13.20 (12.60–13.70)	< 0.001
**Hematokrit (L/L)**	0.45 (0.42–0.48)	0.42 (0.41–0.47)	0.44 (0.41–0.48)	0.838
**Mean corpuscular hemoglobin concentration (g/L)**	331.0 (324.0–338.0)	329.0 (325.0–334.75)	331.0 (324.0–338.0)	0.353
**Mean corpuscular hemoglobin (pg)**	30.20 (29.20–31.20)	30.75 (30.0–31.87)	30.30 (29.28–31.20)	0.005
**Mean corpuscular volume (fL)**	91.10 (88.30–93.80)	93.30 (91.43–96.45)	91.20 (88.40–93.90)	< 0.001
**Hemoglobin (g/L)**	147.0 (136.0–159.0)	147.0 (137.0–158.0)	147.0 (136.0–159.0)	0.710
**Blood sedimentation equation K value**	11.78 (9.48–25.31)	33.67 (26.21–44.64)	12.38 (9.84–27.16)	0.001
**Erythrocyte sedimentation rate (mm/h)**	3.0 (2.0–6.0)	9.50 (6.0–15.0)	3.0 (2.0–7.0)	0.001
**White blood cell count (10**^**9**^**/L)**	5.97 (4.98–7.06)	5.86 (5.15–7.05)	5.96 (4.99–7.06)	0.926
**Neutrophil count (10**^**9**^**/L)**	3.46 (2.81–4.27)	3.38 (2.94–4.12)	3.45 (2.81–4.26)	0.848
**Neutrophil percent (%)**	58.50 (53.32–63.48)	57.95 (53.40–65.10)	58.50 (53.38–63.60)	0.478
**Lymphocyte count (10**^**9**^**/L)**	1.94 (1.58–2.30)	2.0 (1.33–2.40)	1.94 (1.58–2.30)	0.373
**Lymphocyte percentage (%)**	32.90 (28.30–37.70)	32.60 (24.95–37.10)	32.90 (28.17–37.70)	0.281
**Basophil count (10**^**9**^**/L)**	0.02 (0.02–0.04)	0.02 (0.02–0.04)	0.02 (0.02–0.04)	0.904
**Basophil percentage (%)**	0.40 (0.20–0.60)	0.40 (0.30–0.60)	0.40 (0.20–0.60)	0.813
**Eosinophil count (10**^**9**^**/L)**	0.11 (0.07–0.18)	0.12 (0.09–0.17)	0.11 (0.07–0.18)	0.288
**Eosinophil percentage (%)**	1.80 (1.20–2.90)	1.95 (1.40–2.85)	1.80 (1.20–2.90)	0.417
**Monocyte count (10**^**9**^**/L)**	0.33 (0.26–0.41)	0.36 (0.30–0.41)	0.33 (0.26–0.41)	0.240
**Monocyte percentage (%)**	5.55 (4.62–6.70)	5.70 (5.12–7.05)	5.60 (4.70–6.70)	0.218
**CD3 count (cell/ul)**	1151.50 (927.25–1433.25)	995.0 (835.0–1067.0)	1135.0 (907.0–1419.0)	0.089
**CD3 percentage (%)**	67.40 (61.80–73.70)	69.40 (59.70–72.65)	67.80 (61.77–73.75)	0.962
**CD4 count (cell/ul)**	627.0 (503.0–782.25)	719.0 (421.0–734.0)	628.0 (500.0–781.0)	0.599
**CD4 percentage (%)**	37.50 (32.0–43.10)	39.50 (33.80–49.0)	37.70 (32.22–43.18)	0.410
**CD8 count (cell/ul)**	407.0 (304.75–574.25)	296.0 (197.0–305.0)	395.0 (295.0–571.0)	0.019
**CD8 percentage (%)**	24.0 (17.80–29.70)	19.10 (16.30–25.55)	23.55 (17.80–29.72)	0.227
**CD4/CD8 ratio**	1.58 (1.16–2.20)	2.48 (1.11–3.34)	1.58 (1.16–2.25)	0.244
**Blood platelet count (10**^**9**^**/L)**	200.0 (165.0–246.0)	155.50 (141.75–207.50)	199.0 (163.0–245.0)	< 0.001
**Monocyte-to-lymphocyte ratio**	0.17 (0.14–0.22)	0.18 (0.14–0.27)	0.17 (0.14–0.22)	0.109
**Neutrophil-to-lymphocyte ratio**	1.77 (1.42–2.25)	1.74 (1.47–2.67)	1.77 (1.43–2.26)	0.276
**Eosinophil-to-lymphocyte ratio**	0.06 (0.03–0.09)	0.07 (0.04–0.10)	0.06 (0.03–0.09)	0.076
**Basophil-to-lymphocytes ratio**	0.01 (0.01–0.02)	0.02 (0.01–0.02)	0.01 (0.01–0.02)	0.383
**Platelet-to-lymphocyte ratio**	104.74 (82.33–131.76)	89.54 (68.17–137.48)	104.50 (80.84–131.96)	0.172

Data are *n* (%) or median (IQR). *P* value denotes the comparison among mild, moderate, and severe illness groups. The outcomes were designated as non-severe and severe respectively

*Red cell distribution width (SD)*, red blood cell distribution width standard deviation; *Red cell distribution width (CV)*, red blood cell distribution width coefficient of variation

^*^Since only 66% of those older than 80 had been fully vaccinated by late November 2022, the age of 80 was set as the cutting point to minimize the confounding effect of vaccination [10]

### Associations of severe COVID-19 and different hematological parameters

In the time-to-event analysis, we applied unadjusted and three sequential adjusted models to explore the association between severe COVID-19 and different hematological parameters in the baseline model. Although no significant difference was identified in platelet count using all 4 models, outcomes of white blood cell-related parameters and red cell-related parameters varied.

**Fig. 2:**
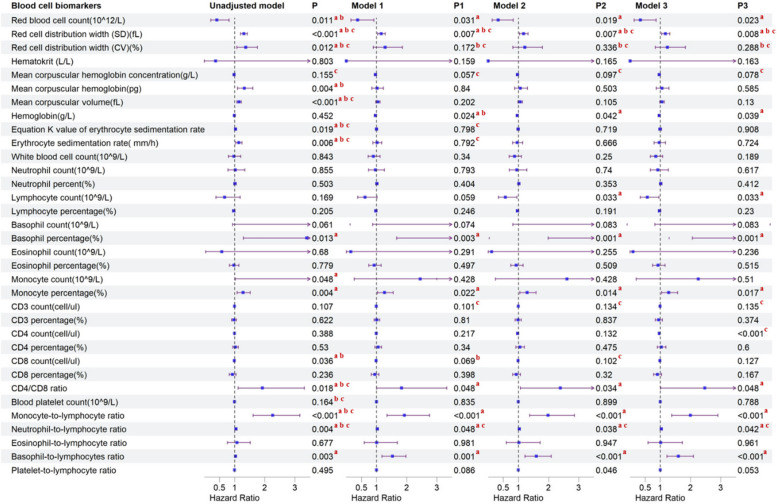
**a** *P* < 0.05 in Cox regression analysis. **b*** P* < 0.05 in bivariable logistic regression analysis; **c** *P* < 0.05 in multivariable ordinal logistic regression analysis. Model 1: adjusted for baseline age, gender, and BMI. Model 2: further adjusted for smoking status and drinking habits. Model 3: further adjusted for hypertension and diabetes

For white blood cell-related parameters, after adjusting age, sex, BMI, smoking status, drinking status, diabetes, and hypertension (model 3, fully adjusted model), severe COVID-19 was significantly associated with a lower level of LymC (adjusted HR [aHR] = 0.571, 95% CI = 0.341–0.955, *P* = 0.033), and a higher level of Bas% (aHR = 6.164, 95% CI = 2.066–18.393, *P* = 0.001) and Mon% (aHR = 1.283, 95% CI = 1.046–1.573, *P* = 0.017). Similar signals were captured in the other 3 models. For lymphocyte-related parameters specifically, only higher levels of CD4/CD8 ratio (aHR = 2.473, 95% CI = 1.009–6.059, *P* = 0.048) were found associated with severe COVID-19 in all of the 4 models with significance. Furthermore, a higher level of monocyte count (HR = 7.693, 95% CI = 1.020–58.009, *P* = 0.048) and lower level of CD8 count (HR = 0.990, 95% CI = 0.981–0.999,* P* = 0.036) were also discovered to have a significant association with severe COVID-19 in unadjusted model (Fig. 2, Additional file 2: Table S1).

For red blood cell-related parameters, after adjusting age, sex, BMI, smoking status, drinking status, diabetes, and hypertension status (model 3, fully adjusted model), severe COVID-19 was significantly associated with a higher level of RDW-SD (aHR = 1.171, 95% CI = 1.042–1.315, *P* = 0.008) and lower level of RBC (aHR = 0.343, 95% CI = 0.136–0.865, *P* = 0.023) and Hb (aHR = 0.966, 95% CI = 0.935–0.998, *P* = 0.039). Similar signals were also identified in the other three models. Further, in the unadjusted model, RDW-CV (HR = 1.371, 95% CI = 1.073–1.752, *P* = 0.012), MCV (HR = 1.150, 95% CI = 1.079–1.227, *P* = 0.201 × 10^−4^), ESR-K (HR = 1.029, 95% CI = 1.005–1.054,* P* = 0.019), and ESR (HR = 1.137, 95% CI = 1.037–1.247, *P* = 0.006) were also discovered to have a significant association with severe COVID-19 (Additional file 2: Table S1).

For ratios between cell population frequencies, after adjusting age, sex, BMI, smoking status, drinking status, diabetes, and hypertension (model 3, fully adjusted model), severe COVID-19 was significantly associated with a higher level of MoLR (aHR = 1.645, 95% CI = 1.258–2.152, *P* < 0.001), NLR (aHR = 1.032, 95% CI = 1.001–1.065, *P* = 0.042), and BLR (aHR = 1.601, 95% CI = 1.223–2.096, *P* < 0.001). Similar signals were also identified in the other three models (Additional file 2: Table S1).

### Sensitivity analysis

We then performed binary and ordinal logistic regression analysis as sensitivity analysis using the same models. Notably, after classifying the 956 non-severe COVID-19 cases into mild (*n* = 921, 92.3%) and moderate (*n* = 35, 3.5%) subgroups, 54.7% (*n* = 504) of the mild subgroups were male, 99.3% (*n* = 915) were less than 80 years old, 60% (*n* = 21) of the moderate subgroups were male, and 100% (*n* = 35) were less than 65 years old (Additional file 2: Table S2).

For white blood cell-related parameters, lower count of CD3, CD4, and CD8 and higher count of CD4/CD8 ratio showed significant association with severe COVID-19 in different models using both kinds of regression analysis (all adjusted OR [aOR] < 1, all *P* < 0.05). For red blood cell-related parameters, both binary and ordinal logistic analyses additionally found a higher level of red cell distribution width (CV) had a significant association with severe COVID-19 (all aOR > 1, all *P* < 0.05), apart from red cell distribution width (SD) (all aOR > 1, all *P* < 0.05). Further, ordinal logistic regression analysis found that the decrease of MCHC was associated with a higher risk of severe COVID-19 (all aOR < 1, all *P* < 0.05). Moreover, severe COVID-19 was found significantly associated with both higher ESR and higher ESR-K whether or not adjusting for age, sex, and BMI (all OR > 1, all *P* < 0.05) (Additional file 2: Table S3-S4).

For ratios between cell population frequencies, higher MoLR and NLR were found significant association with severe COVID-19 in different adjusting models using both binary and ordinal logistic regression analysis (all aOR > 1, all *P* < 0.05). Besides, lower platelet count showed a slight significance associated with severe COVID-19 using either binary or ordinal logistic regression analysis in an unadjusted model (binary logistic regression OR = 0.994, 95% CI = 0.990–0.998, *P* = 0.005; ordinal logistic regression OR = 0.990, 95% CI = 0.984–0.996, *P* = 0.001) (Additional file 2: Table S3-S4).

### Relationship between hematological parameters trajectory and severity of COVID-19

After excluding participants without at least two blood cell tests, 727 COVID-19 patients were left in the trajectory analysis, 35 of which were identified as severe cases. A total of 14 parameters were excluded, 9 of which were with fewer than 10 severe COVID-19 patients and 5 of which did not satisfy the criterion to generate optimal trajectories. The results of the fitting process and trajectories of all hematological parameters can be found in Additional file 2: Table S5-S29 and Additional file 3: Fig. S1-19. As shown in Table 2, in the unadjusted Cox proportional hazards models, the high-increasing RDW-SD trajectory was associated with a higher risk of severe COVID-19 compared with the low-increasing group (HR = 3.654, 95% CI = 1.406–9.497, *P* = 0.008). In terms of the NeuC, the N-shape trajectory was found to have a lower risk of severe COVID-19 than the inverted N-shape trajectory (HR = 0.261, 95% CI = 0.080–0.854, *P* = 0.026). The U-shape trajectory of LymC was identified to be positively associated with severe COVID-19 cases compared with the stable trajectory, and the adjusted models all yielded similar results (all *P* < 0.05).

**Table 2:** Hazard ratios (HRs) and 95% confidence intervals (CIs) for hematological parameters trajectories associated with incident severity of COVID-19

Hematological parameters	Trajectories	Unadjusted model		Model 1		Model 2		Model 3	
		**HR (95% CI)**	***P***	**HR (95% CI)**	***P***	**HR (95% CI)**	***P***	**HR (95% CI)**	***P***
**Red blood cell count**	Decreasing (ref.)								
	Increasing	0.651 [0.318, 1.331]	0.239	1.075 [0.507, 2.278]	0.85	1.039 [0.479, 2.250]	0.923	1.055 [0.488, 2.279]	0.891
**Red cell distribution width (SD)**	Low-increasing (ref.)								
	High-increasing	3.654 [1.406, 9.497]	0.008	1.063 [0.357, 3.169]	0.913	1.043 [0.349, 3.118]	0.941	0.962 [0.308, 3.009]	0.947
**Red cell distribution width (CV)**	Stable (ref.)								
	Decreasing	0.000 [0.000, Inf]	0.997	0.000 [0.000, Inf]	0.997	0.000 [0.000, Inf]	0.997	0.000 [0.000, Inf]	0.997
**Mean corpuscular hemoglobin**	N-shape (ref.)								
	Increasing	0.601 [0.144, 2.514]	0.486	0.436 [0.102, 1.867]	0.263	0.421 [0.098, 1.810]	0.245	0.406 [0.091, 1.811]	0.237
**Mean corpuscular volume**	N-shape (ref.)								
	Increasing	0.732 [0.175, 3.059]	0.669	0.651 [0.154, 2.760]	0.561	0.638 [0.150, 2.711]	0.543	0.685 [0.157, 2.983]	0.614
**White blood cell count**	U-shape (ref.)								
	Decreasing	1.738 [0.861, 3.508]	0.123	0.795 [0.361, 1.752]	0.569	0.744 [0.327, 1.693]	0.482	0.728 [0.315, 1.679]	0.456
**Neutrophil count**	Inverted N shape (ref.)								
	N-shape	0.261 [0.080, 0.854]	0.026	0.481 [0.144, 1.611]	0.236	0.486 [0.144, 1.644]	0.246	0.499 [0.146, 1.699]	0.266
**Lymphocytes count**	stable (ref.)								
	Low-decreasing	0.787 [0.369, 1.677]	0.534	0.969 [0.431, 2.182]	0.94	0.981 [0.433, 2.222]	0.963	0.922 [0.392, 2.169]	0.853
	U-shape	4.287 [1.275, 14.413]	0.019	3.584 [1.039, 12.363]	0.043	3.778 [1.054, 13.544]	0.041	3.823 [1.027, 14.231]	0.045
**Lymphocyte percentage**	Decreasing (ref.)								
	U-shape	2.406 [0.733, 7.891]	0.148	2.662 [0.794, 8.919]	0.113	2.654 [0.784, 8.984]	0.117	2.757 [0.790, 9.621]	0.112
**Basophil count**	Stable (ref.)								
	Increasing	0.364 [0.087, 1.519]	0.166	0.628 [0.145, 2.722]	0.535	0.536 [0.117, 2.457]	0.422	0.558 [0.122, 2.557]	0.453
**Basophil percentage**	Stable (ref.)								
	Inverted U-shape	0.517 [0.071, 3.783]	0.516	1.058 [0.141, 7.935]	0.956	0.957 [0.123, 7.449]	0.967	1.074 [0.134, 8.584]	0.946
**Eosinophil percentage**	N-shape (ref.)								
	Stable	0.711 [0.170, 2.968]	0.64	0.391 [0.091, 1.688]	0.209	0.380 [0.087, 1.651]	0.197	0.338 [0.075, 1.517]	0.157
**Monocyte count**	J-shape (ref.)								
	Inverted U-shape	0.464 [0.109, 1.965]	0.297	0.931 [0.201, 4.317]	0.928	0.866 [0.185, 4.046]	0.855	0.736 [0.148, 3.659]	0.708
**Monocyte percentage**	J-shape (ref.)								
	Inverted U-shape	0.683 [0.209, 2.237]	0.529	0.959 [0.277, 3.325]	0.948	0.983 [0.272, 3.559]	0.979	1.069 [0.292, 3.913]	0.92
**Blood platelet count**	U-shape (ref.)								
	Inverted U-shape	0.492 [0.172, 1.404]	0.185	1.612 [0.521, 4.986]	0.407	1.608 [0.518, 4.997]	0.411	1.678 [0.532, 5.300]	0.377
**Monocyte-to-lymphocyte ratio**	U-shape (ref.)								
	Increasing	0.694 [0.208, 2.314]	0.552	0.407 [0.115, 1.439]	0.163	0.376 [0.103, 1.374]	0.139	0.388 [0.108, 1.398]	0.148
**Neutrophil-to-lymphocyte ratio**	N-shape (ref.)								
	Stable	0.336 [0.102, 1.102]	0.072	0.533 [0.125, 2.276]	0.396	0.541 [0.126, 2.326]	0.409	0.530 [0.120, 2.350]	0.404
**Eosinophil-to-lymphocyte ratio**	Stable (ref.)								
	Increasing	1.808 [0.523, 6.258]	0.350	1.536 [0.416, 5.673]	0.520	1.646 [0.440, 6.162]	0.459	1.574 [0.417, 5.936]	0.503
**Platelet-to-lymphocyte ratio**	N-shape (ref.)								
	Stable	0.878 [0.305, 2.530]	0.809	0.429 [0.145, 1.271]	0.127	0.420 [0.139, 1.271]	0.125	0.436 [0.140, 1.356]	0.151
	U-shape	1.335 [0.376, 4.737]	0.654	0.570 [0.158, 2.059]	0.391	0.541 [0.147, 1.995]	0.356	0.530 [0.142, 1.971]	0.343

Ref. The reference group in the model; Model 1: adjusted for baseline age, gender, and BMI; Model 2: further adjusted for smoking status and drinking habits; Model 3: further adjusted for hypertension and diabetes

*Red cell distribution width (SD)*, red blood cell distribution width standard deviation; *Red cell distribution width (CV)*, red blood cell distribution width coefficient of variation

**Fig. 3:**
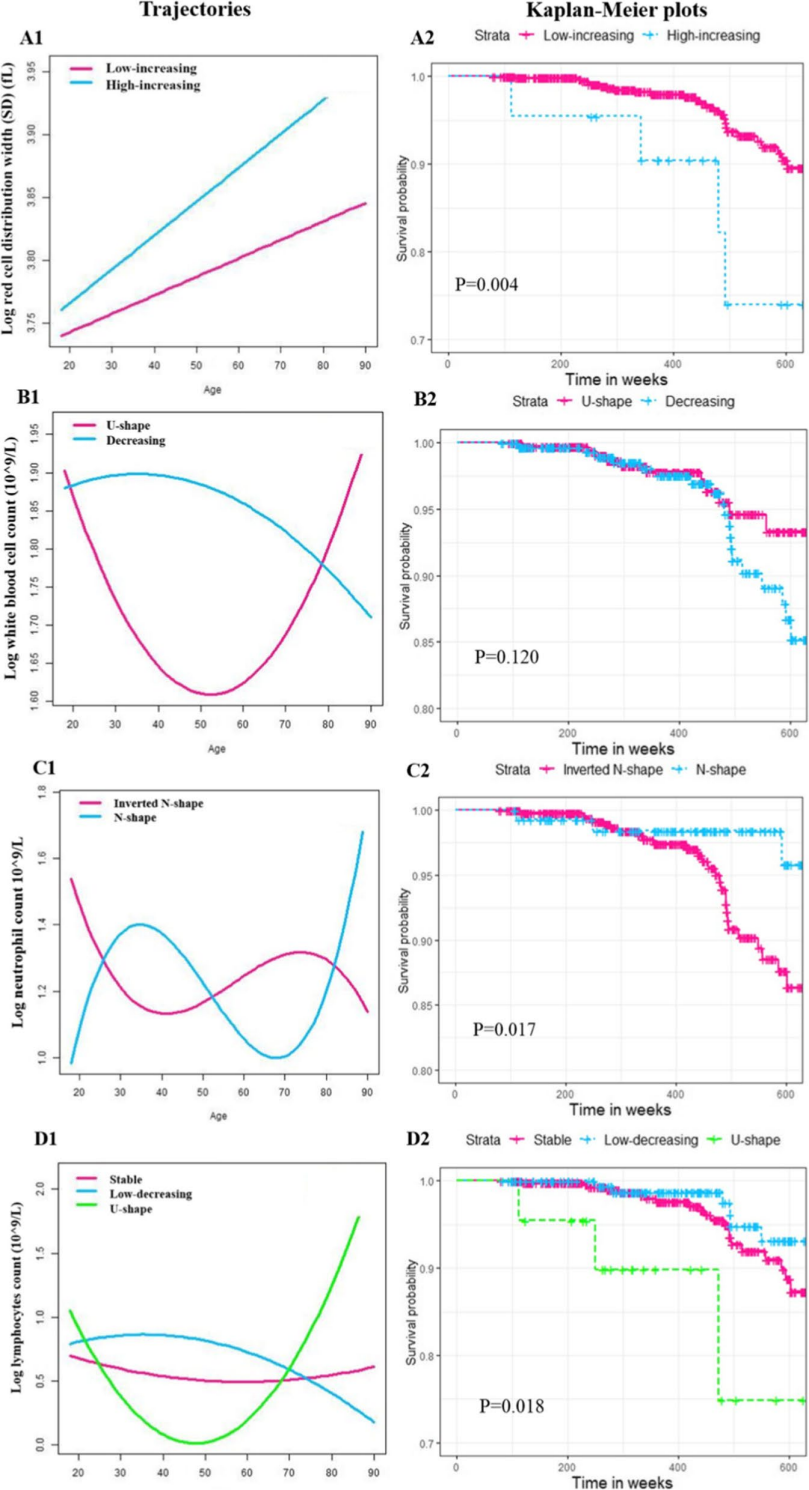
Trajectories prior to COVID-19 and Kaplan–Meier curves of 4 hematological parameters. **A1** Trajectories of red cell distribution width (SD). **B1** Trajectories of white blood cell count. **C1** Trajectories of neutrophil count. **D1** Trajectories of lymphocyte count. **A2** Kaplan–Meier analysis of red cell distribution width (SD). **B2** Kaplan–Meier analysis of white blood cell count. **C2** Kaplan–Meier analysis of neutrophil count. **D2** Kaplan–Meier analysis of lymphocyte count

The binary logistic regression models agreed well with the results of Cox regression (Additional file 2: Table S29). Furthermore, the logistic regression models witnessed the decreasing trajectory of WBC had a higher risk of severe COVID-19, compared with the U-shape trajectory (OR = 2.267, 95% CI = 1.111–4.629, *P* = 0.001). No significant results of the 5 ratio trajectories were found in all the models. Stratifying the patients using trajectory groups of RDW-SD, WBC, NeuC, and LymC, the Kaplan–Meier analysis showed a significant association with the incidence of severe COVID-19 cases (Fig. 3).

## Discussion

Based on the 13-year longitudinal prospective health check-up cohort (WHALE), we demonstrated significant correlations between the risk of severe COVID-19 and different kinds of hematological parameters at the baseline level, accounting for pre-existing conditions. Specifically, subtypes of white blood cells, including basophil, monocyte, lymphocyte, and CD4/CD8 ratio, and ratios between cell population frequencies, including MoLR, NLR, and BLR, were found related to the risk of severe COVID-19. We also observed that abnormality of red cell distribution width (RDW), mean corpuscular hemoglobin concentration (MCHC), and hemoglobin might also be involved in the development of severe COVID-19. The trajectory patterns of RDW-SD and white blood cell count, including lymphocyte and neutrophil, prior to the infection could help further distinguish the higher-risk population of COVID-19 proactively.

The current WHALE cohort was conducted at West China Hospital (WCH) of Sichuan University. Although it is located in Sichuan province, it was still considered to be population-based and can be considered nationally representative, mainly for three reasons. First, WCH is not only the largest medical center in western China but also consistently ranked in second place among all hospitals in China; the healthcare system provides tertiary care for the population of Sichuan (of over 80 million) and other provinces [1, 36, 37]. Second, Sichuan province is not only the largest economy in Western China but also the 18th-largest economy ahead of the GDP of Turkey, as well as the 19th most populous as of 2021. Third, in history, there have been several times of massive resettlement and immigration of people from the neighboring regions in China [38], so the participants in Sichuan province are quite diverse. Therefore, residents recruited in the WHALE cohort in this study are large enough and representative enough to identify specific population health problems geographically, economically, and historically.

The white blood cells, also named leukocytes, are an important and sophisticated group of cells and are primarily involved in inflammatory disease pathogenesis [39]. Some of them are involved in the pathogenesis of several inflammatory immune-mediated disorders, in particular, systemic chronic inflammation [40]. Notably, leukocytes, as inflammation parameters, have been successfully used to prognosticate patients with inflammatory diseases, especially various types of cancers [41–43]. The role of inflammation parameters in severe infectious diseases has also been identified and their ability to predict risk was demonstrated [44]. Therefore, we focused on the association between severe COVID-19 and plentiful leukocyte-related inflammatory parameters prior to COVID-19. Even though very few studies focused on the long-term changes prior to COVID-19, many post-infection studies tried to explore the key role of leukocytes after diagnosis of COVID-19.

In this study, we found that patients with increased basophil percentage prior to infection had a higher risk for severe COVID-19 after adjustment for a series of confounders. For instance, a similar result was also found in a retrospective study with 548 patients which presented that a progressive increase in basophil count was a risk factor for fatal outcomes of COVID-19 by comparing longitudinal variations between on-admission and end hospitalization [45]. Further, a large number of literature data suggested that basophils played an active role in a coordinated adaptive immune response to SARS-CoV-2. Contrary to our results, a decreased basophil count was found in patients especially after the diagnosis of acute and severe COVID-19 [46] and associated with a worse prognosis [47]. The decrease is thought to be due to elevated IL-6 levels resulting from hyperinflammatory cytokine responses which suppress anti-CoV-2 IgG responses in severe cases, leading to an acceleration of basophil depletion [46]. Therefore, the variation of association of basophil cell count underscores the significance of careful study design and accurate measurements for tracking longitudinal changes. Further research is imperative to thoroughly investigate the pathophysiology of basophils in the context of COVID-19 or other pandemics.

Given the potential danger posed by dysregulated cytokine storms which monocytes may contribute to, understanding the role of monocytes in risk prediction is useful for the prevention of severe COVID-19 [48]. Our Cox regression analysis revealed that the baseline monocyte percentage in the severe group was significantly higher than the non-severe group, which was consistent with many previous post-infection studies [49, 50]. For example, Biamonte et al. carried out their single-institutional research with 50 patients and found that monocyte count was one of the main markers discriminating against high- and low-risk groups [51]. By contrast, our study not only included a larger sample size on the basis of a prospective cohort but also unraveled the long-term change of monocytes before COVID-19.

Moreover, lymphocyte count/percentage prior to SARS-CoV-2 infection was with a negative correlation between and the severity of COVID-19 in both our Cox and logistic regression, which is supported by many previous biological and pathological studies. For example, lymphopenia is a widely discussed hematological abnormality linked with the severity of COVID-19 infection and prognosis [52], as COVID-19 encompassed both the innate and adaptive immune responses, which might be caused by a deficient immunological response to viral infection [53]. Since it is similar to other viral inflammatory responses, which hinder lymphopoiesis and elevate lymphocyte apoptosis [54]. Our outcome might provide decision references when facing other infection pandemics.

In addition, identifying the trajectories of lymphocyte count could help distinguish different risk groups for severe COVID-19. Although the mechanism of why the U-shape trajectory group was prone to develop severe COVID-19 is unclear, it could help clinicians and decision-makers to recognize individuals with a high risk of severe COVID-19. Our trajectory analysis used a powerful statistical method (GMM) for uncovering unobserved subpopulations, which might provide valuable insights into heterogeneous developmental trajectories. Previous studies indicated that there might be a potential genetic correlation between the trajectories and the genetic factors [55]. For instance, the trajectories of white blood cells or lymphocyte count might be the results of inborn errors of Type I IFN immunity or autoantibodies against type I IFNs in patients associated with COVID-19 severity [56].

To be specific, it was presented that lower CD8 and higher CD4-to-CD8 ratios were significantly associated with severe COVID-19. A prospective and observational cohort study that analyzed blood samples from 19 patients with COVID-19 ARDS proved that the CD4-to-CD8 ratio was a widely recognized prognostic parameter for disease severity [57]. Therefore, we would highlight the CD4-to-CD8 ratio as an essential parameter to early identify the high-risk population for severe COVID-19.

Interestingly, this study did not discover any significant difference in neutrophil count between severe and non-severe COVID-19 in either Cox or logistic regression, but two trajectories of the neutrophil count, N-shape, and inverted N-shape were finally identified. Even though the two trajectories were not linear-like, the different time-to-event outcomes could help better classify the risk of COVID-19 patients [58]. Based on our results, N-shaped trajectory of neutrophils might potentially increase the incidence of severe COVID-19, which is similar to a previous research by Takayuki and colleagues. They performed a systematic review to find neutrophilia was correlated with severe COVID-19 [51]. The alteration in neutrophil count may be related to cytokine storm induced by virus invasion [52].

Even though the underlying pathology through which this intricate change trajectory impacts COVID-19 requires further investigation, it offers a fresh perspective on the long-term monitoring of patients based on checkup parameters. Our results and analytical examples lay the groundwork for leveraging this association in the prediction of the onset and progression of serious pandemics like COVID-19.

Furthermore, red blood cells (RBC), also named erythrocytes, are the functional component in human circulation, and their main physiological role is to assist gas exchange and transport nutrients to various parts of the body [59]. In our analysis, higher RDW-CV and RDW-SD were found a significant association with severe COVID-19. Although the exact pathophysiology behind the association has yet to be elucidated, numerous reports have indicated the hyperinflammatory state might suppress and destruct the hematopoietic function of bone marrow, resulting in abnormality of RBC size and subsequently elevated RDW levels [60, 61]. Moreover, MCHC and hemoglobin concentration were found negatively correlated with the severity of COVID-19, which was also agreed by previous studies. For example, SARS-CoV-2 might aggravate the disease by directly infecting red blood cell precursor cells and affecting hemoglobin biosynthesis in red blood cells [62]. Some COVID-19 patients might present insufficient blood oxygenation even though their lungs did not appear severely damaged, which indicated a direct involvement of erythrocytes in COVID-19 infections [63].

Ratios between cell population frequencies have a high diagnostic and prognostic value for many infectious and non-communicable chronic diseases, making them extremely important clinically [64]. Our results indicated significant associations of risk of COVID-19 severity with baseline levels of NLR and MoLR, which was agreed by many previous studies [45], even though most of them focused only on the post-infection status. For instance, a retrospective analysis, based on 199 COVID-19 patients, revealed elevated MoLR and NLR might be related to poor survival [65]. Rezaeian et al. also suggested that these two parameters could be applied as a valuable strategy for theragnosis goals and clinical management of COVID-19 [66]. The potential mechanism may be that COVID-19 can activate innate and adaptive immune responses, and elevated MoLR and NLR indicate an inflammatory status and heightened immune system activity [19]. While the exact pathophysiological mechanisms underlying this association remain elusive, it has been observed that basophils, which are implicated in allergic reactions, inflammation, and autoimmune disorders, might play a role in the progression of severe COVID-19 [67].

Except for the above significant associations, our main analysis did not find a significant difference in blood platelet count between severe and non-severe COVID-19. However, some researchers associated thrombocytopenia with critical COVID-19 and higher mortality [68]. Cytokine storm caused by severe COVID-19 is a high-risk factor for disseminated intravascular coagulopathy, which contributed to thrombocytopenia [69]. In addition, Tan et al. performed a retrospective analysis and revealed an inverse relationship between eosinophils and the severity of COVID-19 [70]. These conclusions were both not found in our analysis and needed more study to prove.

These discoveries indicated that impairment of baseline function of the immune or metabolism of energy and nutrients might result in severe COVID-19. The main strengths of the study include that it was based on the current largest health checkup prospective cohort, in which different types of blood cell tests were assessed prior to the pandemic of COVID-19. In addition, it is the first trajectory analysis over 13 years of baseline hematological parameters in participants prior to COVID-19, to the best of our knowledge. Notably, we would emphasize the vital role of lymphocytes, regardless of their count, percentage, and trajectory, which might act as an essential parameter to early identify the high-risk population for severe COVID-19. Our study provides valuable population-based evidence for the associations between severe COVID-19 and hematological parameters at baseline levels before COVID-19, which might help proactively identify high-risk groups of infection, including COVID-19, using similar progressions of hemocyte changes over ages.

In particular, vaccination status might play an essential role in the assessment of COVID-19 severity [71]. However, we would suggest it might only have minor influence on us, mainly for three reasons. First and foremost, our logistic and Cox regression analyses were applied to identify the associations between severe COVID-19 and baseline hematological parameters, which are all reported before COVID-19 happened in 2020; thus, no one would be affected by vaccinations. In addition, even though the COVID-19 vaccine might enhance immune response [72], change red blood cell morphology [73], or even cause thrombocytopenia [74], the alterations of the parameters would all be in the follow-up. Therefore, we would suggest that our trajectories could still track these alterations in the follow-up, as the outcomes were all identified. Moreover, since only 66% of those older than 80 had been fully vaccinated by late November [10], according to data released by the State Council’s joint prevention and control mechanism, we tried to perform a univariable sensitivity analysis excluding patients older than 80 years old (Additional file 2: Table S30). Notably, the results showed no statistically significant disparities between the outcomes before and after this sensitivity analysis for the main parameters we reported. Consequently, the influence of vaccination status on our conclusions appears to be minimal. Given the established connections between vaccination and both COVID-19 severity and blood cells [75], it is imperative to interpret and generalize our findings cautiously considering these factors.

However, the present study has several limitations. First, our analysis only included 998 samples, whereas the influence of the bias from the small sample size would be narrowed by using prospective data and controlling possible confounding variables. Second, the study was not a traditional multicenter study, yet not only WCH owns three independent medical subcenters and four health management checkup centers but also there were a number of participants who were referred from different geographic places for the health alliance of WCH. Thus, we assumed our outcomes were reliable and representative of the Chinese population. Third, we were unable to investigate whether the severe COVID-19 patients had a predisposition to this severity, because of lacking the genetic testing data for all the COVID-19 patients. However, as our previous publication suggested the underlying genetic correlation between blood cells and severe COVID-19 in European ancestry using UK Biobank [76], we believed it is truly a future scientific direction of our West China WHALE cohort, aiming not only for COVID-19 but also for other severe acute respiratory pandemic.

## Conclusion

In conclusion, based on the data from the largest prospective WHALE cohort in western China, we found that several abnormalities of blood cells indicated a substantially increased risk of severe COVID-19 among individuals subsequently infected with SARS-CoV-2. Specifically, increased basophil percentage, monocyte percentage, CD4-to-CD8 ratio, and RDW and decreased lymphocyte count, MCHC, and hemoglobin concentration predicted more severe disease. The trajectory patterns determined in our study might help optimize the allocation of medical resources by defining risk stratification earlier and more accurately. The use of a health checkup cohort in our analysis calls for further investigations focusing on the role of proactive health in the era of long COVID-19.

### Supplementary Information

**Additional file 1.** Supplemental methods.

**Additional file 2: Table S1-S30.** **Table S1.** Cox regression analysis of observational association between severe COVID-19 and hematological parameters. **Table S2** Baseline characteristics of patients with mild, moderate and severe COVID-19. **Table S3.** Binary logistic regression modeling examining factors associated with illness severity. **Table S4.** Multivariable ordinal logistic regression modeling examining factors associated with illness severity. **Table S5-S28.** GMM results of hematological parameters model fitting process. **Table S29.** Logistic regression results of relationship between hematological parameters trajectories and severity of COVID-19. **Table S30.** Results of binary logistic analysis testing the association of hematological parameters and severe COVID-19 cases by age group.

**Additional file 3: Figures S1-S19.** **FigS1-S19** Figures of trajectories for hematological parameters.

## Data Availability

Summary statistics of the data utilized in our study were provided in the Additional file 2. The data will be shared on a reasonable request to the corresponding author.

## Abbreviations

Bas%: Basophil percentage
BasC: Basophil count
BLR: Basophil-to-lymphocyte ratio
ELR: Eosinophil-to-lymphocyte ratio
Eos%: Eosinophil percentage
EosC: Eosinophil count
ESR: Erythrocyte sedimentation rate
ESR-K: Equation *K* value of erythrocyte sedimentation rate
Hb: Hemoglobin
HCT: Hematocrit
Lym%: Lymphocyte percentage
LymC: Lymphocyte count
MCH: Mean corpuscular hemoglobin
MCHC: Mean corpuscular hemoglobin concentration
MCV: Mean corpuscular volume
MoLR: Monocyte-to-lymphocyte ratio
Mon%: Monocyte percentage
MonC: Monocyte count
Neu%: Neutrophil percentage
NeuC: Neutrophil count
NLR: Neutrophil-to-lymphocyte ratio
PLR: Platelet-to-lymphocyte ratio
PLT: Blood platelet count
RBC: Red blood cell count
RDW-CV: Red cell distribution width (CV)
RDW-SD: Red cell distribution width (SD)
WBC: White blood cell count
