# Supplementary material for: Characterization and trajectories of hematological parameters prior to severe COVID-19 based on a large-scale prospective health checkup cohort in western China: a longitudinal study of 13-year follow-up
Source: BMC Med. 2024 Mar 7;22:105. doi: 10.1186/s12916-024-03326-x (PMC10921814; doi:10.1186/s12916-024-03326-x)
Supplement: Supplementary file 1 — Additional file 1. Supplemental methods. [file 12916_2024_3326_MOESM1_ESM.docx]

***Supplemental methods: Latent Class Growth Mixed Model (29-35)***

The GMMwere employed to derive trajectory subgroups for hematological parameters prior to COVID-19 infection. The package lcmm (version 2.0.0) for R (version 4.0.3) was used to fit the GMM models that perform the trajectories with a function of time. To ensure enough people to fit the trajectory, hematological parameters with at least 10 COVID-19 severe cases were included in the trajectory analysis.

The GMM were applied to classify individuals into distinct subgroups which share similar trajectories, and allowing for within-classes heterogeneity. Let *C* indicate the number of latent classes in the population, distributed with probabilities Pc, c=1…,c, with 0≤Pc≤1, and $\sum_{c=1}^{C} P_{c}=1$. Considering $Z_{ij|c}$ as a single outcome variable, observed on individual *i* at times $t_{j}$, where i=1,2,…N; j=0,1,..,J. A mixed effects model specific to the latent class *c* is modeled based on the observed data as the latent classes are unknown. Weighted by the probability of each class $P_{c}$, the joint distribution of the data is considered as the mixture of these distributions. The growth mixture models for continuous outcomes are specified as:

$Z_{ij|c}=\beta_{0i}^{c}+\beta_{1i}^{c}t_{j}+\epsilon_{ij}^{c}$, for c=1,…,C

Where $\beta_{0i}^{c}$=$\beta_{0}^{c}+u_{0i}^{c}$, $\beta_{1i}^{c}$=$\beta_{1}^{c}+u_{1i}^{c}$, $u_{1i}^{c}$=($u_{0i}^{c}$, $u_{1i}^{c}$), and $\epsilon_{ij}^{c}$ capture the distance between the observed data for the i-th individual to the true individual-specific trajectory, specifically for each class c (Fig. 1). The fixed coefficients $\beta_{0}^{c}$ and $\beta_{1}^{c}$ are shared by all individuals, while the error terms $u_{i}^{c}$ are unobserved random components that capture the individual departures from the population average trajectory.


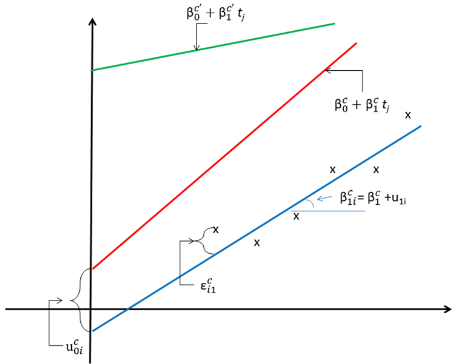


Fig. 1. Graphical representation of latent class growth mixture model. Blue line: individual-specific trajectory; red and green lines: class-specific trajectories; x: observations for individual I.

In initial model parameter determination, the individual growth trajectories within classes were assumed to be heterogeneous by setting the random effects of the models as a linear term of age. Then, the 1-class model structure was used to identify the optimal number of trajectories by testing models in a stepwise fashion up to models with 5 latent classes. For each single class, we assessed the polynomial function of linear, quadratic and cubic to explore the optimal shape of the trajectory.

To aid model convergence, we recentered and scaled age (centered on the median age of the cohort, and divided by 10).

The models with the preferred number of latent classes were chosen based on a combination of theory, interpretability, meaningfulness and fit indices, and was guided by the Guidelines for Reporting on Latent Trajectory Studies checklist. The following criteria was used: 1) an average posterior probability of assignment for each group equal to or greater than 0.7; 2) each group contains at least 2% of participants of the included populations; 3) As the Bayesian information criteria (BIC) indicated goodness of fit, a model with a lower BIC value is preferred.
