# Supplementary material for: Characterization and trajectories of hematological parameters prior to severe COVID-19 based on a large-scale prospective health checkup cohort in western China: a longitudinal study of 13-year follow-up
Source: BMC Med. 2024 Mar 7;22:105. doi: 10.1186/s12916-024-03326-x (PMC10921814; doi:10.1186/s12916-024-03326-x)
Supplement: Supplementary file 2 — Additional file 2: Table S1-S30. Table S1. Cox regression analysis of observational association between severe COVID-19 and hematological parameters. Table S2 Baseline characteristics of patients with mild, moderate and severe COVID-19. Table S3. Binary logistic regression modeling examining factors associated with illness severity. Table S4. Multivariable ordinal logistic regression modeling examining factors associated with illness severity. Table S5-S28. GMM results of hematological parameters model fitting process. Table S29. Logistic regression results of relationship between hematological parameters trajectories and severity of COVID-19. Table S30. Results of binary logistic analysis testing the association of hematological parameters and severe COVID-19 cases by age group. [file 12916_2024_3326_MOESM2_ESM.docx]

## Table S1. Cox regression analysis of observational association between severe COVID-19 and hematological parameters

| **hematological parameters** | **unadjusted** | | **model 1^a^** | | **model 2^b^** | | **model 3^c^** | |
| --- | --- | --- | --- | --- | --- | --- | --- | --- |
|  | **HR (95% CI)** | **P** | **HR (95% CI)** | **P** | **HR (95% CI)** | **P** | **HR (95% CI)** | **P** |
| Red blood cell count（10^12/L） | 0.415 (0.211-0.817) | 0.011 | 0.375 (0.153-0.916) | 0.031 | 0.333 (0.133-0.833) | 0.019 | 0.343 (0.136-0.865) | 0.023 |
| Red cell distribution width (SD)（fL） | 1.307 (1.199-1.425) | <0.001 | 1.160 (1.041-1.292) | 0.007 | 1.173 (1.045-1.317) | 0.007 | 1.171 (1.042-1.315) | 0.008 |
| Red cell distribution width (CV)（%） | 1.371 (1.073-1.752) | 0.012 | 1.289 (0.895-1.857) | 0.172 | 1.212 (0.819-1.793) | 0.336 | 1.237 (0.835-1.833) | 0.288 |
| Hematokrit （L/L） | 0.370 (0.000-936.057) | 0.803 | 0.000 (0.000-24.323) | 0.159 | 0.000 (0.000-29.196) | 0.165 | 0.000 (0.000-29.236) | 0.163 |
| Mean corpuscular hemoglobin concentration（g/L） | 0.981 (0.955-1.007) | 0.155 | 0.968 (0.936-1.001) | 0.057 | 0.971 (0.937-1.005) | 0.097 | 0.968 (0.933-1.004) | 0.078 |
| Mean corpuscular hemoglobin（pg） | 1.325 (1.094-1.605) | 0.004 | 1.019 (0.848-1.226) | 0.84 | 1.069 (0.879-1.300) | 0.503 | 1.057 (0.866-1.290) | 0.585 |
| Mean corpuscular volume（fL） | 1.150 (1.079-1.227) | <0.001 | 1.044 (0.977-1.117) | 0.202 | 1.059 (0.988-1.135) | 0.105 | 1.057 (0.984-1.135) | 0.13 |
| Hemoglobin（g/L） | 0.992 (0.973-1.012) | 0.452 | 0.965 (0.935-0.995) | 0.024 | 0.967 (0.937-0.999) | 0.042 | 0.966 (0.935-0.998) | 0.039 |
| Equation K value of erythrocyte sedimentation rate | 1.029 (1.005-1.054) | 0.019 | 1.004 (0.971-1.038) | 0.798 | 0.992 (0.952-1.034) | 0.719 | 0.997 (0.951-1.045) | 0.908 |
| Erythrocyte sedimentation rate（ mm/h） | 1.137 (1.037-1.247) | 0.006 | 1.019 (0.885-1.173) | 0.792 | 0.963 (0.811-1.143) | 0.666 | 0.965 (0.794-1.173) | 0.724 |
| White blood cell count（10^9/L） | 0.979 (0.796-1.205) | 0.843 | 0.900 (0.725-1.118) | 0.34 | 0.873 (0.693-1.100) | 0.25 | 0.852 (0.670-1.082) | 0.189 |
| Neutrophil count（10^9/L） | 1.025 (0.786-1.338) | 0.855 | 0.965 (0.737-1.263) | 0.793 | 0.950 (0.704-1.283) | 0.74 | 0.922 (0.671-1.267) | 0.617 |
| Neutrophil percent（%） | 1.014 (0.974-1.054) | 0.503 | 1.015 (0.980-1.052) | 0.404 | 1.018 (0.980-1.058) | 0.353 | 1.017 (0.978-1.057) | 0.412 |
| Lymphocyte count（10^9/L） | 0.673 (0.383-1.183) | 0.169 | 0.622 (0.380-1.019) | 0.059 | 0.573 (0.344-0.956) | 0.033 | 0.571 (0.341-0.955) | 0.033 |
| Lymphocyte percentage（%） | 0.972 (0.931-1.015) | 0.205 | 0.978 (0.942-1.016) | 0.246 | 0.973 (0.935-1.014) | 0.191 | 0.975 (0.936-1.016) | 0.23 |
| Basophil count（10^9/L） | 4.827 (0.932-24.994) | 0.061 | 4.020 (0.873-18.502) | 0.074 | 4.604 (0.818-25.906) | 0.083 | 4.639 (0.818-26.319) | 0.083 |
| Basophil percentage（%） | 3.387 (1.293-8.870) | 0.013 | 4.731 (1.672-13.386) | 0.003 | 5.997 (1.989-18.080) | 0.001 | 6.164 (2.066-18.393) | 0.001 |
| Eosinophil count（10^9/L） | 0.577 (0.042-7.856) | 0.68 | 0.151 (0.005-5.025) | 0.291 | 0.117 (0.003-4.708) | 0.255 | 0.100 (0.002-4.488) | 0.236 |
| Eosinophil percentage（%） | 0.977 (0.832-1.147) | 0.779 | 0.928 (0.749-1.151) | 0.497 | 0.929 (0.747-1.156) | 0.509 | 0.929 (0.744-1.160) | 0.515 |
| Monocyte count（10^9/L） | 7.693 (1.020-58.009) | 0.048 | 2.445 (0.267-22.361) | 0.428 | 2.610 (0.243-27.996) | 0.428 | 2.258 (0.200-25.511) | 0.51 |
| Monocyte percentage（%） | 1.281 (1.081-1.517) | 0.004 | 1.267 (1.035-1.552) | 0.022 | 1.290 (1.052-1.582) | 0.014 | 1.283 (1.046-1.573) | 0.017 |
| CD3 count（cell/ul） | 0.997 (0.993-1.001) | 0.107 | 0.995 (0.990-1.001) | 0.101 | 0.996 (0.991-1.001) | 0.134 | 0.993 (0.984-1.002) | 0.135 |
| CD3 percentage（%） | 0.979 (0.901-1.064) | 0.622 | 1.011 (0.925-1.105) | 0.81 | 0.989 (0.892-1.097) | 0.837 | 0.945 (0.835-1.070) | 0.374 |
| CD4 count（cell/ul） | 0.998 (0.993-1.003) | 0.388 | 0.995 (0.987-1.003) | 0.217 | 0.975 (0.944-1.008) | 0.132 | 0.966 (0.959-0.973) | <0.001 |
| CD4 percentage（%） | 1.028 (0.943-1.121) | 0.53 | 1.052 (0.948-1.167) | 0.34 | 1.050 (0.918-1.201) | 0.475 | 1.037 (0.905-1.190) | 0.6 |
| CD8 count（cell/ul） | 0.990 (0.981-0.999) | 0.036 | 0.989 (0.978-1.001) | 0.069 | 0.990 (0.978-1.002) | 0.102 | 0.991 (0.979-1.003) | 0.127 |
| CD8 percentage（%） | 0.930 (0.824-1.049) | 0.236 | 0.946 (0.831-1.077) | 0.398 | 0.931 (0.808-1.072) | 0.32 | 0.903 (0.780-1.044) | 0.167 |
| CD4/CD8 ratio | 1.922 (1.117-3.309) | 0.018 | 1.831 (1.006-3.331) | 0.048 | 2.389 (1.068-5.347) | 0.034 | 2.473 (1.009-6.059) | 0.048 |
| Blood platelet count（10^9/L） | 0.996 (0.989-1.002) | 0.164 | 0.999 (0.992-1.006) | 0.835 | 1.000 (0.993-1.007) | 0.899 | 0.999 (0.992-1.006) | 0.788 |
| Monocyte-to-Lymphocyte Ratio | 1.060 (1.035-1.086) | <0.001 | 1.603 (1.240-2.072) | <0.001 | 1.633 (1.256-2.125) | <0.001 | 1.645 (1.258-2.152) | <0.001 |
| Neutrophil-to-Lymphocyte Ratio | 1.048 (1.015-1.083) | 0.004 | 1.029 (1.000-1.059) | 0.048 | 1.032 (1.002-1.063) | 0.038 | 1.032 (1.001-1.065) | 0.042 |
| Eosinophil-to-Lymphocyte Ratio | 1.076 (0.763-1.516) | 0.677 | 1.006 (0.599-1.690) | 0.981 | 1.018 (0.601-1.723) | 0.947 | 1.013 (0.594-1.729) | 0.961 |
| Basophil-to-Lymphocytes Ratio | 1.030 (1.010-1.051) | 0.003 | 1.530 (1.184-1.976) | 0.001 | 1.594 (1.218-2.086) | <0.001 | 1.601 (1.223-2.096) | <0.001 |
| Platelet-to-Lymphocyte Ratio | 1.000 (0.999-1.001) | 0.495 | 1.001 (1.000-1.002) | 0.086 | 1.001 (1.000-1.002) | 0.046 | 1.001 (1.000-1.002) | 0.053 |

HR=hazard ratio. P value denotes the comparison between non-severe and severe illness group; ^a^Model 1: Adjusted for age, sex and BMI; ^b^Model 2: Model 1+ smoking status and drinking status; ^c^Model 3: Model 2 +hypertension and diabetes history of the patients; red cell distribution width (SD): Red blood cell distribution width standard deviation; red cell distribution width (CV): red blood cell distribution width coefficient of variation

## Table S2. Baseline characteristics of patients with mild, moderate and severe COVID-19

| **Variables** | **Illness Severity** | | |  |  |
| --- | --- | --- | --- | --- | --- |
|  | **mild** | **moderate** | **severe** | **all** | **P** |
| n | 921(92.3%) | 35(3.5%) | 42(4.2%) | 998 |  |
| Age (median [IQR]) | 36.0 [12.0, 88.0] | 38.0 [20.0, 69.0] | 67.5 [28.0, 87.0] | 37.0 (28.0, 48.0) | <0.001 |
| Age_group (%) |  |  |  |  | <0.001 |
| <80 | 915(99.3%) | 35(100.0%) | 36(85.7%) | 986 |  |
| ≥80 | 6(0.7%) | 0(0.0%) | 6(14.3%) | 12 |  |
| Gender (%) |  |  |  |  | 0.001 |
| male | 504(54.7%) | 21(60.0%) | 35(83.3%) | 560 |  |
| female | 417(45.3%) | 14(40.0%) | 7(16.7%) | 428 |  |
| Diabetes (%) |  |  |  |  | 0.001 |
| No diabetes | 885(96.1%) | 34(97.1%) | 34(81.0%) | 953 |  |
| With diabetes | 36(3.9%) | 1(2.9%) | 8(19.0%) | 45 |  |
| Hypertension (%) |  |  |  |  | <0.001 |
| No hypertension | 833(90.4%) | 31(88.6%) | 22(52.4%) | 886 |  |
| With hypertension | 88(9.6%) | 4(11.4%) | 20(47.6%) | 112 |  |
| Smoke (%) |  |  |  |  | 0.180 |
| Never | 718(78.0%) | 26(74.3%) | 28(66.7%) | 772 |  |
| Former | 17(1.8%) | 0(0.0%) | 1(2.4%) | 18 |  |
| Often | 142(15.4%) | 5(14.3%) | 8(19.0%) | 155 |  |
| Occasionally | 44(4.8%) | 4(11.4%) | 5(11.9%) | 53 |  |
| Drink (%) |  |  |  |  | 0.131 |
| Never | 572(62.1%) | 17(48.6%) | 26(61.9%) | 615 |  |
| Former | 3(0.3%) | 0(0.0%) | 1(2.4%) | 4 |  |
| Often | 64(6.9%) | 2(5.7%) | 2(4.8%) | 68 |  |
| Occasionally | 282(30.6%) | 16(45.7%) | 13(31.0%) | 311 |  |
| **Continuous variables (median [IQR])** | |  |  |  | |
| Red blood cell count | 4.87 [4.55,5.22] | 4.99 [4.69,5.41] | 4.73 [4.47,5.12] | 4.87 [4.55,5.23] | 0.062 |
| Red cell distribution width (SD) | 43.50 [41.40,45.60] | 44.40 [42.25,46.65] | 46.95 [45.10,48.65] | 43.60 [41.50,45.80] | <0.001 |
| Red cell distribution width (CV) | 13.10 [12.60,13.60] | 13.60 [13.10,14.05] | 13.75 [13.30,14.30] | 13.20 [12.60,13.70] | <0.001 |
| Hematokrit | 0.45 [0.41,0.48] | 0.51 [0.44,0.52] | 0.42 [0.41,0.47] | 0.44 [0.41,0.48] | 0.512 |
| Mean corpuscular hemoglobin concentration | 331.0 [268.0, 367.0] | 331.0 [291.0, 346.0] | 329.0 [311.0, 351.0] | 331.0 [324.0, 338.0] | 0.604 |
| Mean corpuscular hemoglobin | 30.20 [29.20,31.20] | 30.30 [29.45,31.10] | 30.75 [30.0,31.87] | 30.30 [29.28,31.20] | 0.021 |
| Mean corpuscular volume | 91.10 [88.30,93.80] | 90.70 [88.80,93.15] | 93.30 [91.43,96.45] | 91.20 [88.40,93.90] | 0.001 |
| Hemoglobin | 147.0 [75.0, 217.0] | 153.0 [103.0, 177.0] | 147.0 [99.0, 166.0] | 147.0 (136.0, 159.0) | 0.555 |
| Equation K value of erythrocyte sedimentation rate | 11.50 [9.48,21.14] | 34.50 [15.33,38.32] | 33.67 [26.21,44.64] | 12.38 [9.84,27.16] | 0.001 |
| Erythrocyte sedimentation rate | 3.0 [2.0,5.0] | 6.0 [4.0,10.0] | 9.50 [6.0,15.0] | 3.0 [2.0,7.0] | 0.002 |
| White blood cell count | 5.97 [4.98,7.05] | 6.01 [5.54,7.05] | 5.86 [5.15,7.05] | 5.96 [4.99,7.06] | 0.788 |
| Neutrophil count | 3.45 [2.80,4.27] | 3.56 [3.12,4.68] | 3.38 [2.94,4.12] | 3.45 [2.81,4.26] | 0.432 |
| Neutrophil percent | 58.40 [53.30,63.35] | 61.50 [55.10,66.65] | 57.95 [53.40,65.10] | 58.50 [53.38,63.60] | 0.115 |
| Lymphocyte count | 1.94 [1.59,2.30] | 1.86 [1.54,2.23] | 2.0 [1.33,2.40] | 1.94 [1.58,2.30] | 0.482 |
| Lymphocyte percentage | 33.10 [28.50,37.70] | 30.60 [26.0,37.35] | 32.60 [24.95,37.10] | 32.90 [28.17,37.70] | 0.202 |
| Basophil count | 0.02 [0.02,0.04] | 0.02 [0.01,0.03] | 0.02 [0.02,0.04] | 0.02 [0.02,0.04] | 0.703 |
| Basophil percentage | 0.40 [0.20,0.60] | 0.30 [0.25,0.55] | 0.40 [0.30,0.60] | 0.40 [0.20,0.60] | 0.524 |
| Eosinophil count | 0.11 [0.07,0.18] | 0.12 [0.06,0.19] | 0.12 [0.09,0.17] | 0.11 [0.07,0.18] | 0.566 |
| Eosinophil percentage | 1.80 [1.20,2.90] | 1.70 [1.0,3.0] | 1.95 [1.40,2.85] | 1.80 [1.20,2.90] | 0.659 |
| Monocyte count | 0.33 [0.26,0.41] | 0.31 [0.26,0.36] | 0.36 [0.30,0.41] | 0.33 [0.26,0.41] | 0.331 |
| Monocyte percentage | 5.60 [4.70,6.70] | 5.10 [4.30,6.35] | 5.70 [5.12,7.05] | 5.60 [4.70,6.70] | 0.091 |
| CD3 count | 1,151.5[940.0, 1,429.5] | 1,147.0 [820.0, 1,474.0] | 995.0 [835.0, 1,067.0] | 1135.0 [907.0,1419.0] | 0.226 |
| CD3 percentage | 67.40 [61.95,73.35] | 70.50 [65.55,75.45] | 69.40 [59.70,72.65] | 67.80 [61.77,73.75] | 0.959 |
| CD4 count | 627.0 [509.0,781.0] | 635.50 [459.25,811.75] | 719.0 [421.0,734.0] | 628.0 [500.0,781.0] | 0.862 |
| CD4 percentage | 37.50 [32.0,43.0] | 39.45 [37.12,41.78] | 39.50 [33.80,49.0] | 37.70 [32.22,43.18] | 0.654 |
| CD8 count | 407.0[308.25,571.75] | 460.50 [314.75,606.25] | 296.0 [197.0,305.0] | 395.0 [295.0,571.0] | 0.061 |
| CD8 percentage | 24.0 [17.80,29.65] | 27.20 [24.0,30.40] | 19.10 [16.30,25.55] | 23.55 [17.80,29.72] | 0.398 |
| CD4/CD8 ratio | 1.58 [1.16,2.21] | 1.49 [1.40,1.58] | 2.48 [1.11,3.34] | 1.58 [1.16,2.25] | 0.487 |
| Blood platelet count | 201.0 [165.0,247.0] | 196.0 [163.50,234.50] | 155.50 [141.75,207.50] | 199.0 [163.0,245.0] | 0.002 |
| Monocyte-to-Lymphocyte Ratio | 0.18 [0.14,0.22] | 0.15 [0.13,0.20] | 0.18 [0.14,0.27] | 0.17 [0.14,0.22] | 0.196 |
| Neutrophil-to-Lymphocyte Ratio | 1.77 [1.42,2.24] | 2.01 [1.47,2.58] | 1.74 [1.47,2.67] | 1.77 [1.43,2.26] | 0.139 |
| Eosinophil-to-Lymphocyte Ratio | 0.05 [0.03,0.09] | 0.06 [0.03,0.09] | 0.07 [0.04,0.10] | 0.06 [0.03,0.09] | 0.206 |
| Basophil-to-Lymphocytes Ratio | 0.01 [0.01,0.02] | 0.01 [0.01,0.02] | 0.02 [0.01,0.02] | 0.01 [0.01,0.02] | 0.543 |
| Platelet-to-Lymphocyte Ratio | 104.52 [82.35,131.60] | 109.50 [82.33,129.40] | 89.54 [68.17,137.48] | 104.50 [80.84,131.96] | 0.393 |

Data are n (%) or median (IQR), P value denotes the comparison among mild, moderate and severe illness group. The severity was used as the outcome, Non-hospitalized patients, hospitalized patients and severe patients were designated as mild, moderate and severe respectively.

red cell distribution width (SD): Red blood cell distribution width standard deviation

red cell distribution width (CV): red blood cell distribution width coefficient of variation

## Table S3. Binary logistic regression modeling examining factors associated with illness severity

| hematological parameters | unadjusted | | model 1^a^ | | | model 2^b^ | | | model 3^c^ | |  |
| --- | --- | --- | --- | --- | --- | --- | --- | --- | --- | --- | --- |
|  | **OR (95%CI)** | **P** | **OR (95%CI)** | **P** | **OR (95%CI)** | | **P** | **OR (95%CI)** | | **P** |  |
| Red blood cell count | 0.417 (0.222-0.785) | 0.007 | 0.448 (0.195-1.032) | 0.059 | 0.462 (0.197-1.085) | | 0.076 | 0.480 (0.206-1.117) | | 0.089 |  |
| Red cell distribution width (SD) | 1.340 (1.221-1.471) | <0.001 | 1.173 (1.043-1.319) | 0.008 | 1.189 (1.053-1.343) | | 0.005 | 1.177 (1.044-1.328) | | 0.008 |  |
| Red cell distribution width (CV) | 1.428 (1.135-1.795) | 0.002 | 1.464 (1.029-2.084) | 0.034 | 1.469 (1.025-2.104) | | 0.036 | 1.490 (1.036-2.143) | | 0.032 |  |
| Hematokrit | 0.089 (0.000-145.055) | 0.521 | 0.000 (0.000-6.672) | 0.108 | 0.001 (0.000-30.927) | | 0.18 | 0.001 (0.000-23.535) | | 0.167 |  |
| Mean corpuscular hemoglobin concentration | 0.991 (0.964-1.018) | 0.509 | 0.980 (0.945-1.017) | 0.28 | 0.983 (0.946-1.022) | | 0.386 | 0.981 (0.943-1.019) | | 0.323 |  |
| Mean corpuscular hemoglobin | 1.379 (1.124-1.692) | 0.002 | 1.014 (0.822-1.251) | 0.898 | 1.051 (0.838-1.317) | | 0.666 | 1.031 (0.823-1.292) | | 0.789 |  |
| Mean corpuscular volume | 1.152 (1.074-1.235) | <0.001 | 1.030 (0.954-1.111) | 0.451 | 1.039 (0.960-1.124) | | 0.347 | 1.034 (0.955-1.119) | | 0.411 |  |
| Hemoglobin | 0.992 (0.972-1.011) | 0.403 | 0.971 (0.944-1.000) | 0.047 | 0.975 (0.946-1.004) | | 0.093 | 0.974 (0.945-1.004) | | 0.086 |  |
| Equation K value of erythrocyte sedimentation rate | 1.043 (1.013-1.074) | 0.005 | 1.023 (0.986-1.061) | 0.231 | 1.013 (0.970-1.057) | | 0.561 | 1.012 (0.957-1.071) | | 0.669 |  |
| Erythrocyte sedimentation rate | 1.197 (1.076-1.331) | 0.001 | 1.095 (0.941-1.275) | 0.241 | 1.061 (0.891-1.263) | | 0.505 | 1.105 (0.851-1.435) | | 0.453 |  |
| White blood cell count | 1.029 (0.840-1.260) | 0.784 | 0.897 (0.714-1.127) | 0.35 | 0.895 (0.705-1.137) | | 0.363 | 0.862 (0.675-1.101) | | 0.236 |  |
| Neutrophil count | 1.084 (0.837-1.403) | 0.543 | 0.920 (0.680-1.245) | 0.589 | 0.916 (0.668-1.257) | | 0.587 | 0.877 (0.637-1.209) | | 0.425 |  |
| Neutrophil percent | 1.020 (0.980-1.061) | 0.342 | 1.011 (0.968-1.057) | 0.616 | 1.010 (0.964-1.057) | | 0.682 | 1.007 (0.962-1.054) | | 0.77 |  |
| Lymphocyte count | 0.784 (0.440-1.396) | 0.408 | 0.692 (0.385-1.244) | 0.218 | 0.702 (0.383-1.289) | | 0.254 | 0.683 (0.371-1.255) | | 0.219 |  |
| Lymphocyte percentage | 0.974 (0.932-1.017) | 0.225 | 0.990 (0.945-1.036) | 0.653 | 0.991 (0.944-1.040) | | 0.716 | 0.994 (0.948-1.043) | | 0.818 |  |
| Basophil count | 1.122 (0.206-6.122) | 0.894 | 1.263 (0.206-7.759) | 0.801 | 1.308 (0.194-8.827) | | 0.783 | 1.120 (0.162-7.732) | | 0.908 |  |
| Basophil percentage | 1.231 (0.433-3.502) | 0.697 | 1.795 (0.558-5.777) | 0.327 | 1.881 (0.571-6.198) | | 0.299 | 1.743 (0.533-5.707) | | 0.358 |  |
| Eosinophil count | 0.659 (0.052-8.383) | 0.748 | 0.331 (0.012-9.484) | 0.518 | 0.260 (0.008-8.755) | | 0.453 | 0.180 (0.005-6.954) | | 0.358 |  |
| Eosinophil percentage | 0.978 (0.830-1.152) | 0.789 | 0.963 (0.778-1.191) | 0.726 | 0.951 (0.764-1.185) | | 0.655 | 0.942 (0.751-1.181) | | 0.604 |  |
| Monocyte count | 3.357 (0.340-33.143) | 0.3 | 0.290 (0.017-4.896) | 0.39 | 0.343 (0.018-6.439) | | 0.475 | 0.245 (0.013-4.770) | | 0.353 |  |
| Monocyte percentage | 1.108 (0.928-1.323) | 0.259 | 0.981 (0.776-1.241) | 0.876 | 1.002 (0.789-1.272) | | 0.988 | 1.006 (0.792-1.278) | | 0.96 |  |
| CD3 count | 0.997 (0.994-1.001) | 0.097 | 0.996 (0.991-1.000) | 0.078 | 0.994 (0.989-1.000) | | 0.069 | 0.988 (0.969-1.007) | | 0.211 |  |
| CD3 percentage | 0.973 (0.893-1.060) | 0.526 | 0.997 (0.910-1.094) | 0.957 | 0.978 (0.879-1.089) | | 0.691 | 0.951 (0.840-1.077) | | 0.431 |  |
| CD4 count | 0.998 (0.994-1.003) | 0.532 | 0.996 (0.990-1.003) | 0.259 | 0.994 (0.986-1.002) | | 0.167 | 0.951 (0.879-1.028) | | 0.205 |  |
| CD4 percentage | 1.054 (0.952-1.168) | 0.31 | 1.057 (0.952-1.172) | 0.298 | 1.030 (0.918-1.156) | | 0.611 | 0.998 (0.881-1.130) | | 0.971 |  |
| CD8 count | 0.989 (0.980-0.999) | 0.028 | 0.988 (0.977-1.000) | 0.045 | 0.988 (0.976-1.001) | | 0.068 | 0.992 (0.978-1.005) | | 0.23 |  |
| CD8 percentage | 0.916 (0.816-1.028) | 0.134 | 0.934 (0.827-1.054) | 0.268 | 0.940 (0.824-1.072) | | 0.358 | 0.952 (0.830-1.092) | | 0.485 |  |
| CD4/CD8 ratio | 2.063 (1.117-3.812) | 0.021 | 1.861 (0.979-3.539) | 0.058 | 1.954 (0.937-4.075) | | 0.074 | 1.708 (0.761-3.837) | | 0.195 |  |
| Blood platelet count | 0.990 (0.984-0.996) | 0.001 | 0.997 (0.990-1.004) | 0.413 | 0.997 (0.990-1.004) | | 0.439 | 0.997 (0.990-1.004) | | 0.385 |  |
| Monocyte-to-Lymphocyte Ratio | | 1.440 (1.123-1.847) | 0.004 | 1.212 (0.899-1.634) | 0.208 | 1.250 (0.918-1.701) | | 0.157 | 1.223 (0.901-1.659) | | 0.197 |
| Neutrophil-to-Lymphocyte Ratio | | 1.513 (1.109-2.065) | 0.009 | 1.335 (0.903-1.974) | 0.148 | 1.356 (0.905-2.031) | | 0.140 | 1.335 (0.893-1.996) | | 0.160 |
| Eosinophil-to-Lymphocyte Ratio | | 2.237 (0.053-93.661) | 0.673 | 1.043 (0.006-195.497) | 0.988 | 0.718 (0.003-162.660) | | 0.905 | 0.751 (0.003-185.258) | | 0.919 |
| Basophil-to-Lymphocytes Ratio | | 1.144 (0.874-1.497) | 0.329 | 1.204 (0.877-1.654) | 0.251 | 1.233 (0.889-1.712) | | 0.210 | 1.218 (0.883-1.681) | | 0.230 |
| Platelet-to-Lymphocyte Ratio | | 0.994 (0.985-1.002) | 0.153 | 1.003 (0.994-1.012) | 0.514 | 1.003 (0.994-1.013) | | 0.515 | 1.003 (0.994-1.013) | | 0.477 |

^a^Model 1: Adjusted for age, sex and BMI; ^b^Model 2: Model 1+ smoking status and drinking status; ^c^Model 3: Model 2 +hypertension and diabetes history of the patients; red cell distribution width (SD): Red blood cell distribution width standard deviation; red cell distribution width (CV): red blood cell distribution width coefficient of variation

## Table S4. Multivariable ordinal logistic regression modeling examining factors associated with illness severity

| hematological parameters | unadjusted | | model 1^a^ | | model 2^b^ | | model 3^c^ | |
| --- | --- | --- | --- | --- | --- | --- | --- | --- |
|  | **OR (95% CI)** | **P** | **OR (95% CI)** | **P** | **OR (95% CI)** | **P** | **OR (95% CI)** | **P** |
| Red blood cell count | 0.771 (0.488-1.217) | 0.264 | 0.796 (0.438-1.447) | 0.455 | 0.787 (0.428-1.448) | 0.441 | 0.801 (0.438-1.464) | 0.47 |
| Red cell distribution width (SD) | 1.230 (1.148-1.317) | <0.001 | 1.134 (1.049-1.226) | 0.002 | 1.155 (1.065-1.252) | <0.001 | 1.152 (1.063-1.249) | 0.001 |
| Red cell distribution width (CV) | 1.423 (1.184-1.711) | <0.001 | 1.431 (1.155-1.773) | 0.001 | 1.444 (1.165-1.790) | 0.001 | 1.455 (1.173-1.804) | 0.001 |
| Hematokrit | 1.693 (0.006-471.461) | 0.855 | 0.012 (0.000-26.676) | 0.26 | 0.021 (0.000-56.707) | 0.339 | 0.021 (0.000-53.171) | 0.335 |
| Mean corpuscular hemoglobin concentration | 0.990 (0.989-0.990) | <0.001 | 0.980 (0.974-0.987) | <0.001 | 0.981 (0.974-0.988) | <0.001 | 0.980 (0.973-0.987) | <0.001 |
| Mean corpuscular hemoglobin | 1.115 (0.980-1.269) | 0.097 | 0.951 (0.843-1.073) | 0.416 | 0.966 (0.850-1.097) | 0.593 | 0.960 (0.845-1.090) | 0.527 |
| Mean corpuscular volume | 1.062 (1.012-1.115) | 0.015 | 0.999 (0.953-1.047) | 0.964 | 1.005 (0.956-1.056) | 0.846 | 1.003 (0.955-1.055) | 0.893 |
| Hemoglobin | 0.999 (0.985-1.014) | 0.928 | 0.983 (0.963-1.004) | 0.108 | 0.985 (0.964-1.006) | 0.16 | 0.985 (0.964-1.006) | 0.157 |
| Equation K value of erythrocyte sedimentation rate | 1.046 (1.019-1.073) | 0.001 | 1.034 (1.006-1.063) | 0.018 | 1.024 (0.992-1.057) | 0.144 | 1.028 (0.994-1.064) | 0.106 |
| Erythrocyte sedimentation rate | 1.188 (1.081-1.306) | <0.001 | 1.139 (1.004-1.291) | 0.043 | 1.095 (0.958-1.251) | 0.184 | 1.134 (0.972-1.323) | 0.11 |
| White blood cell count | 1.062 (0.914-1.234) | 0.432 | 1.009 (0.862-1.181) | 0.912 | 1.020 (0.865-1.202) | 0.817 | 0.998 (0.844-1.179) | 0.979 |
| Neutrophil count | 1.152 (0.954-1.393) | 0.142 | 1.094 (0.894-1.339) | 0.383 | 1.116 (0.904-1.378) | 0.306 | 1.089 (0.880-1.347) | 0.432 |
| Neutrophil percent | 1.029 (0.998-1.060) | 0.069 | 1.026 (0.994-1.059) | 0.107 | 1.028 (0.995-1.062) | 0.098 | 1.026 (0.993-1.060) | 0.117 |
| Lymphocyte count | 0.801 (0.519-1.237) | 0.318 | 0.766 (0.496-1.182) | 0.228 | 0.770 (0.497-1.195) | 0.244 | 0.752 (0.483-1.170) | 0.206 |
| Lymphocyte percentage | 0.970 (0.939-1.003) | 0.072 | 0.978 (0.946-1.011) | 0.19 | 0.976 (0.943-1.010) | 0.169 | 0.978 (0.945-1.012) | 0.206 |
| Basophil count | 0.799 (0.212-3.002) | 0.739 | 0.742 (0.193-2.848) | 0.664 | 0.794 (0.202-3.126) | 0.741 | 0.720 (0.181-2.860) | 0.641 |
| Basophil percentage | 0.802 (0.345-1.866) | 0.608 | 0.851 (0.354-2.047) | 0.719 | 0.889 (0.367-2.149) | 0.793 | 0.864 (0.359-2.083) | 0.746 |
| Eosinophil count | 0.683 (0.104-4.470) | 0.691 | 0.397 (0.043-3.674) | 0.416 | 0.390 (0.040-3.787) | 0.417 | 0.335 (0.033-3.426) | 0.356 |
| Eosinophil percentage | 0.973 (0.859-1.102) | 0.664 | 0.948 (0.820-1.096) | 0.468 | 0.946 (0.817-1.096) | 0.46 | 0.943 (0.813-1.095) | 0.441 |
| Monocyte count | 1.462 (0.236-9.046) | 0.683 | 0.442 (0.059-3.309) | 0.426 | 0.512 (0.065-4.050) | 0.526 | 0.399 (0.049-3.262) | 0.391 |
| Monocyte percentage | 0.996 (0.864-1.148) | 0.96 | 0.924 (0.785-1.086) | 0.337 | 0.931 (0.790-1.097) | 0.395 | 0.928 (0.787-1.094) | 0.374 |
| CD3 count | 0.998 (0.996-1.000) | 0.061 | 0.998 (0.995-1.000) | 0.028 | 0.995 (0.991-0.999) | 0.012 | 0.993 (0.989-0.997) | <0.001 |
| CD3 percentage | 0.985 (0.913-1.063) | 0.701 | 1.004 (0.928-1.087) | 0.918 | 0.986 (0.900-1.080) | 0.76 | 0.985 (0.898-1.079) | 0.741 |
| CD4 count | 0.999 (0.995-1.002) | 0.507 | 0.998 (0.993-1.002) | 0.251 | 0.994 (0.987-1.000) | 0.067 | 0.973 (0.967-0.980) | <0.001 |
| CD4 percentage | 1.052 (0.959-1.154) | 0.283 | 1.054 (0.962-1.156) | 0.258 | 1.039 (0.935-1.154) | 0.474 | 1.019 (0.917-1.134) | 0.724 |
| CD8 count | 0.995 (0.989-1.000) | 0.066 | 0.995 (0.989-1.000) | 0.072 | 0.991 (0.984-0.999) | 0.028 | 0.994 (0.986-1.001) | 0.088 |
| CD8 percentage | 0.946 (0.860-1.040) | 0.25 | 0.959 (0.870-1.056) | 0.392 | 0.957 (0.861-1.063) | 0.409 | 0.977 (0.878-1.087) | 0.669 |
| CD4/CD8 ratio | 1.840 (1.037-3.263) | 0.037 | 1.681 (0.931-3.035) | 0.085 | 1.743 (0.920-3.305) | 0.088 | 1.503 (0.764-2.958) | 0.238 |
| Blood platelet count | 0.994 (0.990-0.998) | 0.005 | 0.998 (0.994-1.003) | 0.448 | 0.998 (0.994-1.003) | 0.412 | 0.998 (0.993-1.002) | 0.332 |
| Monocyte-to-Lymphocyte Ratio | 1.024 (1.003-1.045) | 0.024 | 1.140 (0.914-1.422) | 0.244 | 1.168 (0.933-1.463) | 0.176 | 1.149 (0.916-1.441) | 0.230 |
| Neutrophil-to-Lymphocyte Ratio | 1.041 (1.015-1.067) | 0.002 | 1.404 (1.071-1.840) | 0.014 | 1.434 (1.087-1.892) | 0.011 | 1.404 (1.061-1.857) | 0.0175 |
| Eosinophil-to-Lymphocyte Ratio | 1.077 (0.810-1.433) | 0.609 | 1.129 (0.035-36.653) | 0.945 | 1.069 (0.032-36.135) | 0.970 | 0.925 (0.025-34.258) | 0.966 |
| Basophil-to-Lymphocytes Ratio | 1.004 (0.982-1.027) | 0.729 | 1.033 (0.813-1.312) | 0.791 | 1.053 (0.826-1.342) | 0.677 | 1.039 (0.815-1.324) | 0.756 |
| Platelet-to-Lymphocyte Ratio | 1.000 (0.999-1.000) | 0.220 | 1.002 (0.996-1.009) | 0.480 | 1.002 (0.996-1.008) | 0.543 | 1.002 (0.995-1.008) | 0.596 |

Results of multivariate ordinal logistic model using three levels of severity as response. The severity was used as the outcome, Non-hospitalized patients, hospitalized patients and severe patients were designated as mild, moderate and severe respectively; ^a^Model 1: Adjusted for age, sex and BMI; ^b^Model 2: Model 1+ smoking status and drinking status; ^c^Model 3: Model 2 +hypertension and diabetes history of the patients; red cell distribution width (SD): Red blood cell distribution width standard deviation; red cell distribution width (CV): red blood cell distribution width coefficient of variation

## Table S5. GMM results of red blood cell count model fitting process

| Shape of trajectory | No. Latent class | BIC | entropy | Sample size per class (%) | | | | | Mean posterior probabilities |
| --- | --- | --- | --- | --- | --- | --- | --- | --- | --- |
|  |  |  |  | class 1 | class 2 | class 3 | class 4 | class 5 |  |
| Linear | 1 | -10016.13 | 1 | 100 |  |  |  |  |  |
| Quadratic | 1 | -10026.18 | 1 | 100 |  |  |  |  |  |
| Cubic | 1 | -10024.95 | 1 | 100 |  |  |  |  |  |
| Linear | 2 | -9996.36 | 5.4E-04 | 48.42 | 51.58 |  |  |  | 0.5098/0.5093 |
| **Quadratic** | **2** | **-10017.98** | **0.38** | **57.63** | **42.37** |  |  |  | **0.7939/0.7925** |
| Cubic | 2 | -10027.00 | 0.50 | 97.11 | 2.89 |  |  |  | 0.8657/0.6687 |
| Linear | 3 | -9991.34 | 0.62 | 0.41 | 46.49 | 53.09 |  |  | 0.8507/0.8063/0.802 |
| Quadratic | 3 | -10018.08 | 0.61 | 52.13 | 47.18 | 0.69 |  |  | 0.7981/0.7966/0.8624 |
| Cubic | 3 | -10037.72 | 0.83 | 98.35 | 0.69 | 0.96 |  |  | 0.9425/0.9483/0.7821 |
| Linear | 4 | -9997.97 | 0.73 | 0.55 | 0.96 | 55.43 | 43.05 |  | 0.8017/0.8121/0.8355/0.8408 |
| Quadratic | 4 | -10019.39 | 0.74 | 0.83 | 55.16 | 43.33 | 0.69 |  | 0.8278/0.8371/0.8332/0.9067 |
| Cubic | 4 | -10043.91 | 0.73 | 52.96 | 45.53 | 0.69 | 0.83 |  | 0.8335/0.8286/0.9581/0.9318 |
| Linear | 5 | -9981.43 | 0.61 | 0.28 | 0.83 | 50.76 | 22.01 | 26.13 | 0.9587/0.9033/0.7567/0.7341/0.6088 |
| Quadratic | 5 | -9969.05 | 0.39 | 36.31 | 23.80 | 33.15 | 0.69 | 6.05 | 0.4991/0.6024/0.561/0.8544/0.5253 |
| Cubic | 5 | -10019.84 | 0.73 | 45.25 | 0.83 | 52.68 | 0.41 | 0.83 | 0.8043/0.8652/0.8354/0.8963/0.8943 |

No. Latent class: latent class number of the model;

BIC: the Bayesian information Criterion;

The best fitting model is highlighted in bold characters. (NaN: not applicable).

## Table S6. GMM results of red cell distribution width (SD) model fitting process

| Shape of trajectory | No. Latent class | BIC | entropy | Sample size per class (%) | | | | | Mean posterior probabilities |
| --- | --- | --- | --- | --- | --- | --- | --- | --- | --- |
|  |  |  |  | class 1 | class 2 | class 3 | class 4 | class 5 |  |
| Linear | 1 | -10786.48 | 1 | 100 |  |  |  |  |  |
| Quadratic | 1 | -10781.62 | 1 | 100 |  |  |  |  |  |
| Cubic | 1 | -10775.03 | 1 | 100 |  |  |  |  |  |
| **Linear** | **2** | **-10768.33** | **0.48** | **96.84** | **3.16** |  |  |  | **0.8673/0.6614** |
| Quadratic | 2 | -10778.07 | 0.98 | 0.83 | 99.17 |  |  |  | 0.7734/0.9973 |
| Cubic | 2 | -10776.46 | 0.77 | 1.24 | 98.76 |  |  |  | 0.7876/0.9519 |
| Linear | 3 | -10749.03 | 0.53 | 1.79 | 97.66 | 0.55 |  |  | 0.6005/0.8083/0.5278 |
| Quadratic | 3 | -10757.89 | 0.84 | 0.83 | 98.21 | 0.96 |  |  | 0.7756/0.9503/0.6789 |
| Cubic | 3 | -10748.96 | 0.51 | 1.51 | 90.10 | 8.39 |  |  | 0.7279/0.8014/0.6402 |
| Linear | 4 | -10729.26 | 0.39 | 2.89 | 0.00 | 96.56 | 0.55 |  | 0.5358/NaN/0.6903/0.5293 |
| Quadratic | 4 | -10737.53 | 0.58 | 91.33 | 0.83 | 6.19 | 1.65 |  | 0.7822/0.7311/0.7075/0.7009 |
| Cubic | 4 | -10721.67 | 0.48 | 1.65 | 89.96 | 3.71 | 4.68 |  | 0.6527/0.7409/0.6656/0.5742 |
| Linear | 5 | -10710.71 | 0.27 | 34.80 | 3.99 | 0.00 | 59.42 | 1.79 | 0.5798/0.5217/NaN/0.4483/0.5707 |
| Quadratic | 5 | -10704.96 | 0.36 | 63.27 | 2.48 | 3.99 | 4.40 | 25.86 | 0.5969/0.6133/0.7998/0.6157/0.4169 |
| Cubic | 5 | -10694.19 | 0.39 | 0.83 | 69.74 | 19.81 | 6.60 | 3.03 | 0.7009/0.6101/0.4798/0.5825/0.6769 |

No. Latent class: latent class number of the model;

BIC: the Bayesian information Criterion;

The best fitting model is highlighted in bold characters. (NaN: not applicable).

## Table S7. GMM results of red cell distribution width (CV) model fitting process

| Shape of trajectory | No. Latent class | BIC | entropy | Sample size per class (%) | | | | | Mean posterior probabilities |
| --- | --- | --- | --- | --- | --- | --- | --- | --- | --- |
|  |  |  |  | class 1 | class 2 | class 3 | class 4 | class 5 |  |
| Linear | 1 | -10793.76 | 1 | 100 |  |  |  |  |  |
| Quadratic | 1 | -10790.63 | 1 | 100 |  |  |  |  |  |
| Cubic | 1 | -10784.86 | 1 | 100 |  |  |  |  |  |
| Linear | 2 | -10773.99 | 2.36E-04 | 58.32 | 41.68 |  |  |  | 0.5051/0.5071 |
| **Quadratic** | **2** | **-10962.23** | **0.95** | **96.15** | **3.85** |  |  |  | **0.9908/0.9197** |
| Cubic | 2 | -10956.51 | 0.94 | 3.71 | 96.29 |  |  |  | 0.9032/0.9896 |
| Linear | 3 | -10951.30 | 0.51 | 0.00 | 95.87 | 4.13 |  |  | NaN/0.7786/0.9032 |
| Quadratic | 3 | -10939.66 | 0.77 | 95.32 | 0.69 | 3.99 |  |  | 0.9216/0.6903/0.899 |
| Cubic | 3 | -10967.85 | 0.94 | 95.60 | 1.79 | 2.61 |  |  | 0.9821/0.9269/0.8051 |
| Linear | 4 | -10931.54 | 0.22 | 0.00 | 95.32 | 0.00 | 4.68 |  | NaN/0.3387/NaN/0.8309 |
| Quadratic | 4 | -10983.39 | 0.91 | 94.22 | 0.83 | 3.30 | 1.65 |  | 0.9707/0.7652/0.7683/0.9261 |
| Cubic | 4 | -10973.51 | 0.88 | 4.13 | 92.43 | 2.06 | 1.38 |  | 0.7716/0.955/0.8416/0.9842 |
| Linear | 5 | -10911.77 | 0.16 | 95.19 | 0.00 | 0.00 | 0.00 | 4.81 | 0.3188/NaN/NaN/NaN/0.8141 |
| Quadratic | 5 | -10962.65 | 0.61 | 3.85 | 0.69 | 85.28 | 8.53 | 1.65 | 0.7351/0.8121/0.7109/0.6199/0.9312 |
| Cubic | 5 | -10954.04 | 0.62 | 60.11 | 3.99 | 32.19 | 2.34 | 1.38 | 0.7072/0.767/0.6759/0.7611/0.9876 |

No. Latent class: latent class number of the model;

BIC: the Bayesian information Criterion;

The best fitting model is highlighted in bold characters. (NaN: not applicable).

## Table S8. GMM results of hematocrit model fitting process

| Shape of trajectory | No. Latent class | BIC | entropy | Sample size per class (%) | | | | | Mean posterior probabilities |
| --- | --- | --- | --- | --- | --- | --- | --- | --- | --- |
|  |  |  |  | class 1 | class 2 | class 3 | class 4 | class 5 |  |
| Linear | 1 | -9577.98 | 1.00 | 100.00 |  |  |  |  | -9577.98 |
| Quadratic | 1 | -9591.71 | 1.00 | 100.00 |  |  |  |  | -9591.71 |
| Cubic | 1 | -9592.94 | 1.00 | 100.00 |  |  |  |  | -9592.94 |
| Linear | 2 | -9558.22 | 2.60E-04 | 48.42 | 51.58 |  |  |  | -9558.22 |
| Quadratic | 2 | -9671.21 | 0.98 | 98.62 | 1.38 |  |  |  | -9671.21 |
| Cubic | 2 | -9674.14 | 0.97 | 1.65 | 98.35 |  |  |  | -9674.14 |
| Linear | 3 | -9566.37 | 0.51 | 1.10 | 98.90 | 0.00 |  |  | -9566.37 |
| Quadratic | 3 | -9670.41 | 0.65 | 49.52 | 1.24 | 49.24 |  |  | -9670.41 |
| Cubic | 3 | -9668.41 | 0.65 | 1.51 | 50.48 | 48.01 |  |  | -9668.41 |
| Linear | 4 | -9632.17 | 0.77 | 1.79 | 48.83 | 48.56 | 0.83 |  | -9632.17 |
| Quadratic | 4 | -9648.78 | 0.49 | 7.15 | 49.93 | 1.24 | 41.68 |  | -9648.78 |
| Cubic | 4 | -9666.19 | 0.69 | 1.51 | 54.33 | 43.19 | 0.96 |  | -9666.19 |
| Linear | 5 | -9629.57 | 0.95 | 0.41 | 0.14 | 0.55 | 98.35 | 0.55 | -9629.57 |
| Quadratic | 5 | -9641.32 | 0.59 | 0.96 | 12.38 | 2.75 | 47.87 | 36.04 | -9641.32 |
| Cubic | 5 | -9662.79 | 0.60 | 2.06 | 48.83 | 1.24 | 0.96 | 46.91 | -9662.79 |

No. Latent class: latent class number of the model;

BIC: the Bayesian information Criterion;

The best fitting model is highlighted in bold characters. (NaN: not applicable).

## Table S9. GMM results of mean corpuscular hemoglobin concentration model fitting process

| Shape of trajectory | No. Latent class | BIC | entropy | Sample size per class (%) | | | | | Mean posterior probabilities |
| --- | --- | --- | --- | --- | --- | --- | --- | --- | --- |
|  |  |  |  | class 1 | class 2 | class 3 | class 4 | class 5 |  |
| Linear | 1 | -14951.82 | 1.00 | 100.00 |  |  |  |  |  |
| Quadratic | 1 | -14948.22 | 1.00 | 100.00 |  |  |  |  |  |
| Cubic | 1 | -14944.18 | 1.00 | 100.00 |  |  |  |  |  |
| Linear | 2 | -14932.05 | 1.98E-04 | 47.59 | 52.41 |  |  |  | 0.5064/0.5059 |
| Quadratic | 2 | -14996.02 | 0.98 | 1.24 | 98.76 |  |  |  | 0.8894/0.9973 |
| Cubic | 2 | -14985.21 | 0.98 | 1.24 | 98.76 |  |  |  | 0.8907/0.9964 |
| Linear | 3 | -14979.54 | 0.60 | 1.38 | 98.62 | 0.00 |  |  | 0.875/0.838/NaN |
| Quadratic | 3 | -14977.51 | 0.57 | 1.38 | 11.00 | 87.62 |  |  | 0.8407/0.6574/0.8032 |
| Cubic | 3 | -14961.26 | 0.67 | 1.10 | 94.91 | 3.99 |  |  | 0.9212/0.8653/0.6825 |
| Linear | 4 | -14959.78 | 0.26 | 1.51 | 0.00 | 98.49 | 0.00 |  | 0.8243/NaN/0.4318/NaN |
| Quadratic | 4 | -14956.66 | 0.62 | 1.38 | 81.84 | 15.13 | 1.65 |  | 0.8812/0.7713/0.7248/0.7418 |
| Cubic | 4 | -14993.26 | 0.84 | 0.28 | 96.42 | 1.65 | 1.65 |  | 1/0.9333/0.6735/0.8165 |
| Linear | 5 | -14940.01 | 0.16 | 1.65 | 0.00 | 98.35 | 0.00 | 0.00 | 0.777/NaN/0.2879/NaN/NaN |
| Quadratic | 5 | -14933.65 | 0.44 | 1.38 | 4.81 | 71.25 | 20.36 | 2.20 | 0.882/0.5797/0.5861/0.603/0.7847 |
| Cubic | 5 | -14967.03 | 0.74 | 0.28 | 3.03 | 1.65 | 92.85 | 2.20 | 1/0.6489/0.7183/0.8699/0.7724 |

No. Latent class: latent class number of the model;

BIC: the Bayesian information Criterion;

The best fitting model is highlighted in bold characters. (NaN: not applicable).

## Table S10. GMM results of mean corpuscular hemoglobin model fitting process

| Shape of trajectory | No. Latent class | BIC | entropy | Sample size per class (%) | | | | | Mean posterior probabilities |
| --- | --- | --- | --- | --- | --- | --- | --- | --- | --- |
|  |  |  |  | class 1 | class 2 | class 3 | class 4 | class 5 |  |
| Linear | 1 | -12591.62 | 1.00 | 100.00 |  |  |  |  |  |
| Quadratic | 1 | -12585.04 | 1.00 | 100.00 |  |  |  |  |  |
| Cubic | 1 | -12587.44 | 1.00 | 100.00 |  |  |  |  |  |
| Linear | 2 | -12571.86 | 4.74E-05 | 24.07 | 75.93 |  |  |  | 0.5036/0.5026 |
| Quadratic | 2 | -13017.60 | 0.99 | 96.70 | 3.30 |  |  |  | 0.9991/0.9934 |
| **Cubic** | **2** | **-13061.61** | **0.98** | **3.99** | **96.01** |  |  |  | **0.9862/0.9959** |
| Linear | 3 | -12998.71 | 0.65 | 3.03 | 96.97 | 0.00 |  |  | 0.984/0.8662/NaN |
| Quadratic | 3 | -13070.07 | 0.96 | 96.01 | 2.89 | 1.10 |  |  | 0.9919/0.9926/0.8225 |
| Cubic | 3 | -13028.66 | 0.54 | 3.99 | 96.01 | 0.00 |  |  | 0.9862/0.7868/NaN |
| Linear | 4 | -12978.94 | 0.27 | 3.03 | 0.00 | 96.97 | 0.00 |  | 0.984/NaN/0.4523/NaN |
| Quadratic | 4 | -13008.50 | 0.83 | 0.41 | 3.30 | 96.29 | 0.00 |  | 0.9851/0.9945/0.9371/NaN |
| Cubic | 4 | -12995.72 | 0.25 | 3.99 | 0.00 | 96.01 | 0.00 |  | 0.9862/NaN/0.3929/NaN |
| Quadratic | 5 | -13060.59 | 0.41 | 1.24 | 2.89 | 95.87 | 0.00 | 0.00 | 0.9455/0.9599/0.4454/NaN/NaN |
| Cubic | 5 | -12962.78 | 0.17 | 4.13 | 0.00 | 95.87 | 0.00 | 0.00 | 0.9626/NaN/0.2746/NaN/NaN |

No. Latent class: latent class number of the model;

BIC: the Bayesian information Criterion;

The best fitting model is highlighted in bold characters. (NaN: not applicable).

## Table S11. GMM results of mean corpuscular volume model fitting process

| Shape of trajectory | No. Latent class | BIC | entropy | Sample size per class (%) | | | | | Mean posterior probabilities |
| --- | --- | --- | --- | --- | --- | --- | --- | --- | --- |
|  |  |  |  | class 1 | class 2 | class 3 | class 4 | class 5 |  |
| Linear | 1 | -13700.38 | 1.00 | 100.00 |  |  |  |  |  |
| Quadratic | 1 | -13695.50 | 1.00 | 100.00 |  |  |  |  |  |
| Cubic | 1 | -13690.85 | 1.00 | 100.00 |  |  |  |  |  |
| Linear | 2 | -13680.61 | 8.02E-05 | 43.19 | 56.81 |  |  |  | 0.5037/0.5029 |
| Quadratic | 2 | -13985.91 | 0.98 | 96.97 | 3.03 |  |  |  | 0.996/0.9859 |
| **Cubic** | **2** | **-14002.95** | **0.96** | **3.71** | **96.29** |  |  |  | **0.9777/0.9922** |
| Linear | 3 | -14046.36 | 0.97 | 2.20 | 96.29 | 1.51 |  |  | 0.8643/0.9943/0.866 |
| Quadratic | 3 | -14023.64 | 0.96 | 95.74 | 2.48 | 1.79 |  |  | 0.99/0.9807/0.8606 |
| Cubic | 3 | -13970.73 | 0.39 | 3.71 | 72.08 | 24.21 |  |  | 0.976/0.5734/0.5508 |
| Linear | 4 | -13958.23 | 0.25 | 3.16 | 96.84 | 0.00 | 0.00 |  | 0.9711/0.3981/NaN/NaN |
| Quadratic | 4 | -14037.46 | 0.97 | 0.41 | 2.48 | 95.32 | 1.79 |  | 0.9381/0.9816/0.9907/0.8667 |
| Cubic | 4 | -14037.75 | 0.83 | 1.93 | 92.85 | 3.44 | 1.79 |  | 0.9349/0.928/0.6696/0.9564 |
| Linear | 5 | -13938.47 | 0.17 | 3.30 | 0.00 | 96.70 | 0.00 | 0.00 | 0.9396/NaN/0.2739/NaN/NaN |
| Quadratic | 5 | -14035.41 | 0.85 | 0.41 | 2.20 | 1.38 | 89.41 | 6.60 | 0.9003/0.8916/0.8894/0.9269/0.7003 |
| Cubic | 5 | -14101.99 | 0.95 | 2.06 | 0.55 | 94.77 | 1.51 | 1.10 | 0.86/0.8921/0.9807/0.7792/0.9324 |

No. Latent class: latent class number of the model;

BIC: the Bayesian information Criterion;

The best fitting model is highlighted in bold characters. (NaN: not applicable).

## Table S12. GMM results of hemoglobin model fitting process

| Shape of trajectory | No. Latent class | BIC | entropy | Sample size per class (%) | | | | | Mean posterior probabilities |
| --- | --- | --- | --- | --- | --- | --- | --- | --- | --- |
|  |  |  |  | class 1 | class 2 | class 3 | class 4 | class 5 |  |
| Linear | 1 | -9144.35 | 1.00 | 100.00 |  |  |  |  |  |
| Quadratic | 1 | -9151.43 | 1.00 | 100.00 |  |  |  |  |  |
| Cubic | 1 | -9158.99 | 1.00 | 100.00 |  |  |  |  |  |
| Linear | 2 | -9124.59 | 4.90E-04 | 48.56 | 51.44 |  |  |  | 0.5092/0.5087 |
| Quadratic | 2 | -9307.48 | 0.98 | 98.35 | 1.65 |  |  |  | 0.9972/0.9243 |
| Cubic | 2 | -9368.68 | 0.99 | 1.65 | 98.35 |  |  |  | 0.9617/0.9974 |
| Linear | 3 | -9152.52 | 0.56 | 1.79 | 98.21 | 0.00 |  |  | 0.7866/0.8238/NaN |
| Quadratic | 3 | -9307.53 | 0.71 | 55.43 | 43.05 | 1.51 |  |  | 0.8498/0.8571/0.9576 |
| Cubic | 3 | -9380.39 | 0.95 | 1.65 | 97.52 | 0.83 |  |  | 0.9239/0.9879/0.8109 |
| Linear | 4 | -9132.76 | 0.25 | 2.20 | 97.80 | 0.00 | 0.00 |  | 0.7059/0.5023/NaN/NaN |
| Quadratic | 4 | -9333.92 | 0.95 | 97.11 | 0.28 | 0.96 | 1.65 |  | 0.9859/0.9987/0.7987/0.8898 |
| Cubic | 4 | -9319.45 | 0.77 | 1.38 | 2.34 | 51.44 | 44.84 |  | 0.9798/0.8075/0.8802/0.8431 |
| Linear | 5 | -9112.99 | 0.15 | 2.34 | 0.00 | 0.00 | 97.66 | 0.00 | 0.6799/NaN/NaN/0.2974/NaN |
| Quadratic | 5 | -9314.67 | 0.86 | 0.28 | 1.51 | 0.69 | 95.87 | 1.65 | 0.9983/0.6758/0.7048/0.9373/0.8691 |
| Cubic | 5 | -9286.52 | 0.58 | 1.38 | 2.34 | 51.17 | 26.82 | 18.29 | 0.9796/0.8076/0.7898/0.4703/0.4729 |

No. Latent class: latent class number of the model;

BIC: the Bayesian information Criterion;

The best fitting model is highlighted in bold characters. (NaN: not applicable).

## Table S13. GMM results of white blood cell count model fitting process

| Shape of trajectory | No. Latent class | BIC | entropy | Sample size per class (%) | | | | | Mean posterior probabilities |
| --- | --- | --- | --- | --- | --- | --- | --- | --- | --- |
|  |  |  |  | class 1 | class 2 | class 3 | class 4 | class 5 |  |
| Linear | 1 | -2282.63 | 1.00 | 100.00 |  |  |  |  |  |
| Quadratic | 1 | -2289.36 | 1.00 | 100.00 |  |  |  |  |  |
| Cubic | 1 | -2283.46 | 1.00 | 100.00 |  |  |  |  |  |
| Linear | 2 | -2267.86 | 0.17 | 53.23 | 46.77 |  |  |  | 0.6529/0.678 |
| **Quadratic** | **2** | **-2275.27** | **0.24** | **54.88** | **45.12** |  |  |  | **0.7276/0.7221** |
| Cubic | 2 | -2286.43 | 0.31 | 83.36 | 16.64 |  |  |  | 0.787/0.6891 |
| Linear | 3 | -2248.23 | 0.18 | 72.49 | 1.24 | 26.27 |  |  | 0.5886/0.4774/0.5638 |
| Quadratic | 3 | -2258.96 | 0.23 | 33.84 | 33.01 | 33.15 |  |  | 0.591/0.543/0.6218 |
| Cubic | 3 | -2267.69 | 0.33 | 60.39 | 9.49 | 30.12 |  |  | 0.6728/0.6187/0.6683 |
| Linear | 4 | -2235.63 | 0.35 | 3.44 | 42.37 | 53.09 | 1.10 |  | 0.6237/0.5472/0.5896/0.7143 |
| Quadratic | 4 | -2240.67 | 0.32 | 17.61 | 32.19 | 43.60 | 6.60 |  | 0.6214/0.563/0.5352/0.658 |
| Cubic | 4 | -2239.16 | 0.29 | 53.65 | 22.42 | 13.48 | 10.45 |  | 0.6154/0.425/0.5697/0.593 |
| Linear | 5 | -2215.86 | 0.25 | 3.44 | 72.35 | 22.28 | 0.00 | 1.93 | 0.6237/0.4725/0.3978/NaN/0.5818 |
| Quadratic | 5 | -2219.72 | 0.39 | 5.36 | 44.29 | 43.60 | 3.30 | 3.44 | 0.6442/0.6346/0.5354/0.564/0.7356 |
| Cubic | 5 | -2221.28 | 0.42 | 4.95 | 11.97 | 58.05 | 22.42 | 2.61 | 0.659/0.5936/0.6255/0.5441/0.7495 |

No. Latent class: latent class number of the model;

BIC: the Bayesian information Criterion;

The best fitting model is highlighted in bold characters. (NaN: not applicable).

## Table S14. GMM results of neutrophil count model fitting process

| Shape of trajectory | No. Latent class | BIC | entropy | Sample size per class (%) | | | | | Mean posterior probabilities |
| --- | --- | --- | --- | --- | --- | --- | --- | --- | --- |
|  |  |  |  | class 1 | class 2 | class 3 | class 4 | class 5 |  |
| Linear | 1 | -207.12 | 1.00 | 100.00 |  |  |  |  |  |
| Quadratic | 1 | -209.74 | 1.00 | 100.00 |  |  |  |  |  |
| Cubic | 1 | -203.16 | 1.00 | 100.00 |  |  |  |  |  |
| Linear | 2 | -187.35 | 2.84E-03 | 52.27 | 47.73 |  |  |  | 0.5197/0.5224 |
| Quadratic | 2 | -191.62 | 0.64 | 96.56 | 3.44 |  |  |  | 0.9216/0.6498 |
| **Cubic** | **2** | **-189.88** | **0.20** | **80.19** | **19.81** |  |  |  | **0.7266/0.6616** |
| Linear | 3 | -171.09 | 0.26 | 2.61 | 85.83 | 11.55 |  |  | 0.6023/0.637/0.6606 |
| Quadratic | 3 | -170.45 | 0.52 | 93.54 | 1.79 | 4.68 |  |  | 0.8222/0.6091/0.6346 |
| Cubic | 3 | -173.93 | 0.43 | 76.62 | 20.08 | 3.30 |  |  | 0.7468/0.6295/0.6479 |
| Linear | 4 | -159.53 | 0.50 | 2.75 | 0.55 | 83.49 | 13.20 |  | 0.5788/0.8624/0.7327/0.6403 |
| Quadratic | 4 | -154.54 | 0.65 | 0.96 | 92.30 | 2.61 | 4.13 |  | 0.7077/0.8497/0.5896/0.6646 |
| Cubic | 4 | -157.51 | 0.54 | 1.24 | 19.81 | 75.79 | 3.16 |  | 0.7847/0.6298/0.7546/0.6676 |
| Linear | 5 | -145.44 | 0.44 | 3.30 | 1.10 | 39.61 | 51.31 | 4.68 | 0.5648/0.6916/0.5746/0.6091/0.7172 |
| Quadratic | 5 | -137.10 | 0.50 | 1.65 | 44.98 | 46.91 | 2.61 | 3.85 | 0.7606/0.6122/0.6475/0.593/0.6846 |
| Cubic | 5 | -128.66 | 0.47 | 0.83 | 20.91 | 52.41 | 22.42 | 3.44 | 0.8306/0.6306/0.5936/0.6232/0.7436 |

No. Latent class: latent class number of the model;

BIC: the Bayesian information Criterion;

The best fitting model is highlighted in bold characters. (NaN: not applicable).

## Table S15. GMM results of neutrophil percentage model fitting process

| Shape of trajectory | No. Latent class | BIC | entropy | Sample size per class (%) | | | | | Mean posterior probabilities |
| --- | --- | --- | --- | --- | --- | --- | --- | --- | --- |
|  |  |  |  | class 1 | class 2 | class 3 | class 4 | class 5 |  |
| Linear | 1 | -5688.81 | 1.00 | 100.00 |  |  |  |  |  |
| Quadratic | 1 | -5682.22 | 1.00 | 100.00 |  |  |  |  |  |
| Cubic | 1 | -5677.67 | 1.00 | 100.00 |  |  |  |  |  |
| Linear | 2 | -5669.04 | 4.07E-04 | 48.42 | 51.58 |  |  |  | 0.5085/0.5079 |
| Quadratic | 2 | -5684.85 | 0.90 | 98.21 | 1.79 |  |  |  | 0.981/0.8428 |
| Cubic | 2 | -5696.82 | 0.97 | 1.10 | 98.90 |  |  |  | 0.9508/0.9957 |
| Linear | 3 | -5689.50 | 0.61 | 1.38 | 4.95 | 93.67 |  |  | 0.8254/0.6831/0.8343 |
| Quadratic | 3 | -5705.93 | 0.85 | 1.93 | 95.87 | 2.20 |  |  | 0.7985/0.9546/0.8178 |
| Cubic | 3 | -5672.92 | 0.59 | 1.24 | 88.31 | 10.45 |  |  | 0.899/0.8187/0.6957 |
| Linear | 4 | -5677.90 | 0.58 | 2.34 | 1.38 | 91.61 | 4.68 |  | 0.6348/0.7488/0.7788/0.6467 |
| Quadratic | 4 | -5687.04 | 0.57 | 2.75 | 48.14 | 47.59 | 1.51 |  | 0.7799/0.7253/0.7079/0.8236 |
| Cubic | 4 | -5668.67 | 0.59 | 1.65 | 3.16 | 5.64 | 89.55 |  | 0.7955/0.7607/0.5251/0.7776 |
| Linear | 5 | -5664.96 | 0.50 | 1.93 | 0.96 | 9.22 | 59.01 | 28.89 | 0.7096/0.8141/0.5869/0.6849/0.5662 |
| Quadratic | 5 | -5667.08 | 0.47 | 2.75 | 37.41 | 56.95 | 1.51 | 1.38 | 0.7456/0.5894/0.6651/0.6578/0.8441 |
| Cubic | 5 | -5648.47 | 0.54 | 1.65 | 1.93 | 24.07 | 64.24 | 8.12 | 0.8376/0.8635/0.6071/0.7109/0.5818 |

No. Latent class: latent class number of the model;

BIC: the Bayesian information Criterion;

The best fitting model is highlighted in bold characters. (NaN: not applicable).

## Table S16. GMM results of Lymphocyte count model fitting process

| Shape of trajectory | No. Latent class | BIC | entropy | Sample size per class (%) | | | | | Mean posterior probabilities |
| --- | --- | --- | --- | --- | --- | --- | --- | --- | --- |
|  |  |  |  | class 1 | class 2 | class 3 | class 4 | class 5 |  |
| Linear | 1 | -1007.70 | 1.00 | 100.00 |  |  |  |  |  |
| Quadratic | 1 | -1003.37 | 1.00 | 100.00 |  |  |  |  |  |
| Cubic | 1 | -997.98 | 1.00 | 100.00 |  |  |  |  |  |
| Linear | 2 | -987.94 | 4.34E-04 | 50.76 | 49.24 |  |  |  | 0.5084/0.5086 |
| Quadratic | 2 | -1019.57 | 0.99 | 99.72 | 0.28 |  |  |  | 0.9991/0.7986 |
| Cubic | 2 | -1047.85 | 0.89 | 98.90 | 1.10 |  |  |  | 0.9786/0.8306 |
| Linear | 3 | -993.02 | 0.71 | 0.28 | 95.74 | 3.99 |  |  | 0.7917/0.89/0.6438 |
| **Quadratic** | **3** | **-999.56** | **0.53** | **69.05** | **27.79** | **3.16** |  |  | **0.7725/0.7518/0.7566** |
| Cubic | 3 | -1026.53 | 0.62 | 93.54 | 5.23 | 1.24 |  |  | 0.8495/0.6603/0.8099 |
| Linear | 4 | -1017.52 | 0.78 | 0.28 | 3.03 | 96.56 | 0.14 |  | 0.8378/0.6378/0.8941/1 |
| Quadratic | 4 | -1012.69 | 0.67 | 0.69 | 10.87 | 88.31 | 0.14 |  | 0.787/0.6654/0.8249/1 |
| Cubic | 4 | -1001.43 | 0.47 | 8.12 | 83.63 | 7.02 | 1.24 |  | 0.5932/0.7298/0.5664/0.8017 |
| Linear | 5 | -965.01 | 0.68 | 0.28 | 9.08 | 71.80 | 18.57 | 0.28 | 0.8906/0.7233/0.7916/0.7535/0.9008 |
| Quadratic | 5 | -994.54 | 0.65 | 0.55 | 70.01 | 6.60 | 22.56 | 0.28 | 0.867/0.7626/0.7457/0.7414/0.7779 |
| Cubic | 5 | -977.00 | 0.49 | 9.63 | 80.47 | 1.51 | 7.29 | 1.10 | 0.6129/0.6944/0.6515/0.6175/0.7845 |

No. Latent class: latent class number of the model;

BIC: the Bayesian information Criterion;

The best fitting model is highlighted in bold characters. (NaN: not applicable).

## Table S17. GMM results of lymphocyte percentage model fitting process

| Shape of trajectory | No. Latent class | BIC | entropy | Sample size per class (%) | | | | | Mean posterior probabilities |
| --- | --- | --- | --- | --- | --- | --- | --- | --- | --- |
|  |  |  |  | class 1 | class 2 | class 3 | class 4 | class 5 |  |
| Linear | 1 | -1806.08 | 1.00 | 100.00 |  |  |  |  |  |
| Quadratic | 1 | -1800.42 | 1.00 | 100.00 |  |  |  |  |  |
| Cubic | 1 | -1794.03 | 1.00 | 100.00 |  |  |  |  |  |
| Linear | 2 | -1786.32 | 4.45E-04 | 47.18 | 52.82 |  |  |  | 0.5088/0.5079 |
| **Quadratic** | **2** | **-1819.87** | **0.83** | **96.42** | **3.58** |  |  |  | **0.9642/0.7978** |
| Cubic | 2 | -1810.63 | 0.82 | 96.01 | 3.99 |  |  |  | 0.9631/0.7905 |
| Linear | 3 | -1797.05 | 0.38 | 4.81 | 54.61 | 40.58 |  |  | 0.7456/0.6211/0.6389 |
| Quadratic | 3 | -1796.52 | 0.63 | 95.19 | 1.24 | 3.58 |  |  | 0.8662/0.6738/0.7845 |
| Cubic | 3 | -1793.86 | 0.80 | 1.38 | 95.87 | 2.75 |  |  | 0.6969/0.9401/0.8198 |
| Linear | 4 | -1784.24 | 0.60 | 3.44 | 65.06 | 31.36 | 0.14 |  | 0.7863/0.7728/0.6605/0.9867 |
| Quadratic | 4 | -1774.67 | 0.55 | 22.83 | 1.51 | 72.63 | 3.03 |  | 0.6747/0.6432/0.7499/0.8313 |
| Cubic | 4 | -1764.64 | 0.48 | 11.28 | 2.06 | 83.77 | 2.89 |  | 0.6299/0.6862/0.6781/0.7782 |
| Linear | 5 | -1765.70 | 0.56 | 3.44 | 67.95 | 6.33 | 22.15 | 0.14 | 0.7977/0.6818/0.6901/0.6874/0.9893 |
| Quadratic | 5 | -1755.71 | 0.53 | 1.10 | 19.12 | 3.16 | 2.48 | 74.14 | 0.6969/0.6333/0.6568/0.7685/0.72 |
| Cubic | 5 | -1742.46 | 0.57 | 3.71 | 90.23 | 1.79 | 3.44 | 0.83 | 0.6321/0.7636/0.6335/0.6468/0.8161 |

No. Latent class: latent class number of the model;

BIC: the Bayesian information Criterion;

The best fitting model is highlighted in bold characters. (NaN: not applicable).

## Table S18. GMM results of basophil count model fitting process

| Shape of trajectory | No. Latent class | BIC | entropy | Sample size per class (%) | | | | | Mean posterior probabilities |
| --- | --- | --- | --- | --- | --- | --- | --- | --- | --- |
|  |  |  |  | class 1 | class 2 | class 3 | class 4 | class 5 |  |
| Linear | 1 | -19179.74 | 1.00 | 100.00 |  |  |  |  |  |
| Quadratic | 1 | -19176.69 | 1.00 | 100.00 |  |  |  |  |  |
| Cubic | 1 | -19171.45 | 1.00 | 100.00 |  |  |  |  |  |
| Linear | 2 | -19159.97 | 3.87E-04 | 52.13 | 47.87 |  |  |  | 0.5078/0.5085 |
| **Quadratic** | **2** | **-19243.56** | **0.68** | **87.48** | **12.52** |  |  |  | **0.9214/0.8032** |
| Cubic | 2 | -19236.85 | 0.70 | 12.10 | 87.90 |  |  |  | 0.8179/0.9274 |
| Linear | 3 | -19231.77 | 0.31 | 81.02 | 0.00 | 18.98 |  |  | 0.48/NaN/0.7429 |
| Quadratic | 3 | -19238.31 | 0.65 | 78.82 | 14.44 | 6.74 |  |  | 0.8675/0.7106/0.7695 |
| Cubic | 3 | -19225.48 | 0.47 | 9.63 | 80.06 | 10.32 |  |  | 0.7058/0.7446/0.7798 |
| Linear | 4 | -19212.01 | 0.22 | 0.00 | 78.13 | 0.00 | 21.87 |  | NaN/0.3466/NaN/0.6851 |
| Quadratic | 4 | -19226.12 | 0.58 | 29.02 | 9.35 | 55.57 | 6.05 |  | 0.6454/0.7317/0.7905/0.7919 |
| Cubic | 4 | -19213.74 | 0.48 | 9.22 | 77.85 | 8.94 | 3.99 |  | 0.7195/0.6741/0.6705/0.7345 |
| Linear | 5 | -19192.24 | 0.18 | 0.00 | 76.20 | 0.00 | 0.00 | 23.80 | NaN/0.255/NaN/NaN/0.6485 |
| Quadratic | 5 | -19216.57 | 0.53 | 21.05 | 57.63 | 6.05 | 12.38 | 2.89 | 0.5913/0.7133/0.5639/0.6717/0.8719 |
| Cubic | 5 | -19218.25 | 0.47 | 59.01 | 11.14 | 14.86 | 12.24 | 2.75 | 0.5855/0.6243/0.6388/0.667/0.8601 |

No. Latent class: latent class number of the model;

BIC: the Bayesian information Criterion;

The best fitting model is highlighted in bold characters. (NaN: not applicable).

## Table S19. GMM results of basophil percentage model fitting process

| Shape of trajectory | No. Latent class | BIC | entropy | Sample size per class (%) | | | | | Mean posterior probabilities |
| --- | --- | --- | --- | --- | --- | --- | --- | --- | --- |
|  |  |  |  | class 1 | class 2 | class 3 | class 4 | class 5 |  |
| Linear | 1 | 258.29 | 1.00 | 100.00 |  |  |  |  |  |
| Quadratic | 1 | 245.88 | 1.00 | 100.00 |  |  |  |  |  |
| Cubic | 1 | 252.33 | 1.00 | 100.00 |  |  |  |  |  |
| Linear | 2 | 180.00 | 0.85 | 94.50 | 5.50 |  |  |  | 0.9668/0.8199 |
| **Quadratic** | **2** | **174.06** | **0.83** | **94.50** | **5.50** |  |  |  | **0.9638/0.8328** |
| Cubic | 2 | 187.17 | 0.83 | 94.50 | 5.50 |  |  |  | 0.9637/0.8331 |
| Linear | 3 | 199.77 | 0.32 | 92.30 | 0.00 | 7.70 |  |  | 0.5014/NaN/0.7079 |
| Quadratic | 3 | 174.20 | 0.60 | 78.82 | 16.37 | 4.81 |  |  | 0.8177/0.7293/0.7979 |
| Cubic | 3 | 188.75 | 0.54 | 9.63 | 85.56 | 4.81 |  |  | 0.7716/0.7785/0.8381 |
| Linear | 4 | 219.54 | 0.19 | 91.06 | 0.00 | 0.00 | 8.94 |  | 0.3357/NaN/NaN/0.6499 |
| Quadratic | 4 | 198.80 | 0.38 | 42.92 | 30.95 | 21.46 | 4.68 |  | 0.5594/0.5158/0.6328/0.8026 |
| Cubic | 4 | 193.76 | 0.54 | 68.23 | 8.39 | 19.81 | 3.58 |  | 0.733/0.7041/0.701/0.8866 |
| Linear | 5 | 217.88 | 0.23 | 27.37 | 0.00 | 66.16 | 0.00 | 6.46 | 0.4842/NaN/0.322/NaN/0.7033 |
| Quadratic | 5 | 197.47 | 0.48 | 71.80 | 17.19 | 2.89 | 4.40 | 3.71 | 0.674/0.642/0.6236/0.6213/0.8351 |
| Cubic | 5 | 213.58 | 0.45 | 18.71 | 61.76 | 11.00 | 4.95 | 3.58 | 0.6763/0.5798/0.6085/0.7005/0.8698 |

No. Latent class: latent class number of the model;

BIC: the Bayesian information Criterion;

The best fitting model is highlighted in bold characters. (NaN: not applicable).

## Table S20. GMM results of eosinophil count model fitting process

| Shape of trajectory | No. Latent class | BIC | entropy | Sample size per class (%) | | | | | Mean posterior probabilities |
| --- | --- | --- | --- | --- | --- | --- | --- | --- | --- |
|  |  |  |  | class 1 | class 2 | class 3 | class 4 | class 5 |  |
| Linear | 1 | -6134.91 | 1.00 | 100.00 |  |  |  |  |  |
| Quadratic | 1 | -6128.43 | 1.00 | 100.00 |  |  |  |  |  |
| Cubic | 1 | -6123.31 | 1.00 | 100.00 |  |  |  |  |  |
| Linear | 2 | -6115.14 | 5.53E-04 | 59.56 | 40.44 |  |  |  | 0.5072/0.5106 |
| Quadratic | 2 | -6549.19 | 0.99 | 99.17 | 0.83 |  |  |  | 0.9989/0.9902 |
| Cubic | 2 | -6540.31 | 0.99 | 0.83 | 99.17 |  |  |  | 0.9906/0.9986 |
| Linear | 3 | -6381.07 | 0.72 | 0.00 | 99.17 | 0.83 |  |  | NaN/0.91/0.9724 |
| Quadratic | 3 | -6692.04 | 0.92 | 9.63 | 89.55 | 0.83 |  |  | 0.8472/0.9785/0.9724 |
| Cubic | 3 | -6700.45 | 0.92 | 91.75 | 7.29 | 0.96 |  |  | 0.9741/0.9021/0.9806 |
| Linear | 4 | -6361.30 | 0.39 | 0.00 | 99.17 | 0.00 | 0.83 |  | NaN/0.6807/NaN/0.9724 |
| Quadratic | 4 | -6783.13 | 0.92 | 1.38 | 13.07 | 84.73 | 0.83 |  | 0.9942/0.8737/0.9679/0.9787 |
| Cubic | 4 | -6770.94 | 0.91 | 13.07 | 1.93 | 84.04 | 0.96 |  | 0.8604/0.8976/0.9688/0.9765 |
| Linear | 5 | -6485.82 | 0.41 | 87.62 | 11.55 | 0.00 | 0.00 | 0.83 | 0.5563/0.7913/NaN/NaN/0.9308 |
| Quadratic | 5 | -6639.85 | 0.36 | 12.10 | 0.00 | 85.69 | 1.38 | 0.83 | 0.7649/NaN/0.4042/0.5947/0.9715 |
| Cubic | 5 | -6726.29 | 0.84 | 17.88 | 3.58 | 2.61 | 74.97 | 0.96 | 0.7634/0.7988/0.949/0.9295/0.9903 |

No. Latent class: latent class number of the model;

BIC: the Bayesian information Criterion;

The best fitting model is highlighted in bold characters. (NaN: not applicable).

## Table S21. GMM results of eosinophil percentage model fitting process

| Shape of trajectory | No. Latent class | BIC | entropy | Sample size per class (%) | | | | | Mean posterior probabilities |
| --- | --- | --- | --- | --- | --- | --- | --- | --- | --- |
|  |  |  |  | class 1 | class 2 | class 3 | class 4 | class 5 |  |
| Linear | 1 | 12587.67 | 1.00 | 100.00 |  |  |  |  |  |
| Quadratic | 1 | 12591.44 | 1.00 | 100.00 |  |  |  |  |  |
| Cubic | 1 | 12597.00 | 1.00 | 100.00 |  |  |  |  |  |
| Linear | 2 | 12607.43 | 6.67E-05 | 57.50 | 42.50 |  |  |  | 0.5026/0.5036 |
| Quadratic | 2 | 12236.83 | 0.99 | 99.45 | 0.55 |  |  |  | 0.9988/1 |
| **Cubic** | **2** | **12468.89** | **0.93** | **3.85** | **96.15** |  |  |  | **0.8608/0.9873** |
| Linear | 3 | 12381.55 | 0.37 | 0.00 | 98.76 | 1.24 |  |  | NaN/0.5088/0.8489 |
| Quadratic | 3 | 12107.64 | 0.95 | 95.05 | 4.26 | 0.69 |  |  | 0.9856/0.8938/0.9479 |
| Cubic | 3 | 12112.09 | 0.92 | 93.54 | 0.69 | 5.78 |  |  | 0.9772/0.9537/0.8763 |
| Linear | 4 | 12209.16 | 0.50 | 0.00 | 88.17 | 11.42 | 0.41 |  | NaN/0.5058/0.8018/1 |
| Quadratic | 4 | 12040.04 | 0.88 | 16.51 | 79.92 | 2.89 | 0.69 |  | 0.8504/0.9549/0.9408/0.9822 |
| Cubic | 4 | 12046.20 | 0.89 | 81.84 | 14.72 | 0.69 | 2.75 |  | 0.9623/0.8531/0.9651/0.9055 |
| Linear | 5 | 12228.92 | 0.35 | 0.00 | 0.00 | 87.21 | 12.38 | 0.41 | NaN/NaN/0.3842/0.7643/1 |
| Quadratic | 5 | 12066.39 | 0.56 | 77.58 | 0.69 | 0.00 | 18.84 | 2.89 | 0.5103/0.9823/NaN/0.7957/0.9409 |
| Cubic | 5 | 12044.18 | 0.82 | 79.50 | 13.62 | 3.99 | 0.55 | 2.34 | 0.9211/0.7486/0.7489/1/0.9474 |

No. Latent class: latent class number of the model;

BIC: the Bayesian information Criterion;

The best fitting model is highlighted in bold characters. (NaN: not applicable).

## Table S22. GMM results of monocyte count model fitting process

| Shape of trajectory | No. Latent class | BIC | entropy | Sample size per class (%) | | | | | Mean posterior probabilities |
| --- | --- | --- | --- | --- | --- | --- | --- | --- | --- |
|  |  |  |  | class 1 | class 2 | class 3 | class 4 | class 5 |  |
| Linear | 1 | -6029.31 | 1.00 | 100.00 |  |  |  |  |  |
| Quadratic | 1 | -6044.43 | 1.00 | 100.00 |  |  |  |  |  |
| Cubic | 1 | -6043.71 | 1.00 | 100.00 |  |  |  |  |  |
| Linear | 2 | -6009.54 | 5.09E-04 | 52.96 | 47.04 |  |  |  | 0.5083/0.5093 |
| **Quadratic** | **2** | **-6083.20** | **0.74** | **91.06** | **8.94** |  |  |  | **0.942/0.8138** |
| Cubic | 2 | -6074.49 | 0.75 | 8.80 | 91.20 |  |  |  | 0.8027/0.9451 |
| Linear | 3 | -6062.57 | 0.61 | 67.68 | 27.37 | 4.95 |  |  | 0.8443/0.7147/0.7753 |
| Quadratic | 3 | -6085.39 | 0.64 | 79.50 | 16.78 | 3.71 |  |  | 0.8441/0.755/0.7813 |
| Cubic | 3 | -6069.14 | 0.64 | 78.82 | 17.47 | 3.71 |  |  | 0.8416/0.7607/0.7683 |
| Linear | 4 | -6042.80 | 0.40 | 61.76 | 33.29 | 0.00 | 4.95 |  | 0.5906/0.6679/NaN/0.7754 |
| Quadratic | 4 | -6082.12 | 0.54 | 19.94 | 74.14 | 1.79 | 4.13 |  | 0.7212/0.7449/0.724/0.7852 |
| Cubic | 4 | -6066.84 | 0.56 | 6.19 | 72.90 | 18.29 | 2.61 |  | 0.6268/0.7515/0.7281/0.8056 |
| Linear | 5 | -6023.04 | 0.32 | 0.00 | 56.26 | 0.00 | 38.79 | 4.95 | NaN/0.4232/NaN/0.6237/0.7753 |
| Quadratic | 5 | -6061.71 | 0.42 | 19.81 | 50.48 | 1.51 | 23.52 | 4.68 | 0.6308/0.5149/0.7513/0.65/0.766 |
| Cubic | 5 | -6040.45 | 0.43 | 18.02 | 23.52 | 49.38 | 5.36 | 3.71 | 0.6104/0.661/0.5014/0.5975/0.8221 |

No. Latent class: latent class number of the model;

BIC: the Bayesian information Criterion;

The best fitting model is highlighted in bold characters. (NaN: not applicable).

## Table S23. GMM results of monocyte percentage model fitting process

| Shape of trajectory | No. Latent class | BIC | entropy | Sample size per class (%) | | | | | Mean posterior probabilities |
| --- | --- | --- | --- | --- | --- | --- | --- | --- | --- |
|  |  |  |  | class 1 | class 2 | class 3 | class 4 | class 5 |  |
| Linear | 1 | 12043.75 | 1.00 | 100.00 |  |  |  |  |  |
| Quadratic | 1 | 12041.53 | 1.00 | 100.00 |  |  |  |  |  |
| Cubic | 1 | 12040.91 | 1.00 | 100.00 |  |  |  |  |  |
| Linear | 2 | 12019.06 | 0.66 | 83.91 | 16.09 |  |  |  | 0.9208/0.7784 |
| **Quadratic** | **2** | **12011.72** | **0.60** | **84.73** | **15.27** |  |  |  | **0.8977/0.7915** |
| Cubic | 2 | 12019.82 | 0.60 | 15.13 | 84.87 |  |  |  | 0.7904/0.8956 |
| Linear | 3 | 12036.92 | 0.42 | 34.94 | 49.66 | 15.41 |  |  | 0.602/0.7252/0.7394 |
| Quadratic | 3 | 12018.89 | 0.44 | 54.61 | 32.74 | 12.65 |  |  | 0.7089/0.6681/0.7617 |
| Cubic | 3 | 12031.21 | 0.43 | 51.86 | 34.94 | 13.20 |  |  | 0.6984/0.659/0.7558 |
| Linear | 4 | 12041.06 | 0.49 | 68.91 | 11.00 | 0.83 | 19.26 |  | 0.6362/0.7096/0.7314/0.7283 |
| Quadratic | 4 | 12038.87 | 0.39 | 31.91 | 38.65 | 16.78 | 12.65 |  | 0.6448/0.5254/0.5908/0.7149 |
| Cubic | 4 | 12033.39 | 0.40 | 5.23 | 34.53 | 42.92 | 17.33 |  | 0.6971/0.5915/0.5991/0.6945 |
| Linear | 5 | 12058.40 | 0.46 | 68.23 | 0.96 | 12.93 | 0.41 | 17.47 | 0.5729/0.5294/0.6866/0.8647/0.7056 |
| Quadratic | 5 | 12063.15 | 0.39 | 7.15 | 42.50 | 6.46 | 30.12 | 13.76 | 0.7393/0.4958/0.5855/0.5561/0.7087 |
| Cubic | 5 | 12062.65 | 0.36 | 15.54 | 15.27 | 13.48 | 46.91 | 8.80 | 0.6326/0.4952/0.4745/0.525/0.6781 |

No. Latent class: latent class number of the model;

BIC: the Bayesian information Criterion;

The best fitting model is highlighted in bold characters. (NaN: not applicable).

## Table S24. GMM results of blood platelet count model fitting process

| Shape of trajectory | No. Latent class | BIC | entropy | Sample size per class (%) | | | | | Mean posterior probabilities |
| --- | --- | --- | --- | --- | --- | --- | --- | --- | --- |
|  |  |  |  | class 1 | class 2 | class 3 | class 4 | class 5 |  |
| Linear | 1 | 34834.12 | 1.00 | 100.00 |  |  |  |  |  |
| Quadratic | 1 | 34838.97 | 1.00 | 100.00 |  |  |  |  |  |
| Cubic | 1 | 34839.22 | 1.00 | 100.00 |  |  |  |  |  |
| Linear | 2 | 34853.88 | 5.34E-04 | 51.03 | 48.97 |  |  |  | 0.5096/0.51 |
| **Quadratic** | **2** | **34784.30** | **0.53** | **81.98** | **18.02** |  |  |  | 0.8707/0.7747 |
| Cubic | 2 | 34792.11 | 0.52 | 80.33 | 19.67 |  |  |  | 0.8641/0.7751 |
| Linear | 3 | 34836.19 | 0.26 | 81.71 | 0.00 | 18.29 |  |  | 0.6297/NaN/0.6956 |
| Quadratic | 3 | 34777.90 | 0.45 | 36.31 | 50.21 | 13.48 |  |  | 0.6977/0.7226/0.7495 |
| Cubic | 3 | 34793.63 | 0.46 | 65.20 | 17.33 | 17.47 |  |  | 0.7423/0.699/0.7377 |
| Linear | 4 | 34855.96 | 0.35 | 77.44 | 0.00 | 0.00 | 22.56 |  | 0.5155/NaN/NaN/0.6403 |
| Quadratic | 4 | 34797.26 | 0.45 | 14.58 | 17.47 | 55.02 | 12.93 |  | 0.7415/0.6863/0.6109/0.7118 |
| Cubic | 4 | 34798.63 | 0.44 | 49.66 | 33.29 | 13.48 | 3.58 |  | 0.6312/0.6729/0.7467/0.6764 |
| Linear | 5 | 34913.16 | 0.00 | 0.00 | 90.78 | 0.00 | 0.00 | 9.22 | NaN/0.2436/NaN/NaN/0.2365 |
| Quadratic | 5 | 34837.03 | 0.63 | 68.78 | 14.44 | 0.00 | 0.00 | 16.78 | 0.7546/0.6618/NaN/NaN/0.7266 |
| Cubic | 5 | 34813.53 | 0.45 | 39.89 | 5.36 | 34.66 | 12.65 | 7.43 | 0.6137/0.573/0.6094/0.7533/0.5637 |

No. Latent class: latent class number of the model;

BIC: the Bayesian information Criterion;

The best fitting model is highlighted in bold characters. (NaN: not applicable).

## Table S25. GMM results of monocyte-to-lymphocyte ratio model fitting process

| Shape of trajectory | No. Latent class | BIC | entropy | Sample size per class (%) | | | | | Mean posterior probabilities |
| --- | --- | --- | --- | --- | --- | --- | --- | --- | --- |
|  |  |  |  | class 1 | class 2 | class 3 | class 4 | class 5 |  |
| Linear | 1 | 4588.69 | 1.00 | 100.00 |  |  |  |  |  |
| Quadratic | 1 | 4592.99 | 1.00 | 100.00 |  |  |  |  |  |
| Cubic | 1 | 4599.20 | 1.00 | 100.00 |  |  |  |  |  |
| Linear | 2 | 4607.35 | 0.00 | 48.01 | 51.99 |  |  |  | 0.5101/0.5097 |
| **Quadratic** | **2** | **4593.95** | **0.70** | **6.57** | **93.43** |  |  |  | **0.7651/0.9292** |
| Cubic | 2 | 4596.81 | 0.86 | 3.19 | 96.81 |  |  |  | 0.7984/0.9701 |
| Linear | 3 | 4602.24 | 0.49 | 3.39 | 78.49 | 18.13 |  |  | 0.7301/0.7793/0.5664 |
| Quadratic | 3 | 4611.95 | 0.41 | 8.96 | 80.48 | 10.56 |  |  | 0.6991/0.7082/0.689 |
| Cubic | 3 | 4621.24 | 0.46 | 4.58 | 84.26 | 11.16 |  |  | 0.7768/0.7048/0.7314 |
| Linear | 4 | 4615.65 | 0.31 | 5.38 | 42.23 | 37.25 | 15.14 |  | 0.7334/0.4558/0.5364/0.6104 |
| Quadratic | 4 | 4636.83 | 0.22 | 10.96 | 0.00 | 60.36 | 28.69 |  | 0.6389/NaN/0.3781/0.4863 |
| Cubic | 4 | 4639.11 | 0.48 | 1.20 | 78.49 | 8.57 | 11.75 |  | 0.8571/0.6922/0.6527/0.6698 |
| Linear | 5 | 4625.78 | 0.36 | 2.19 | 33.67 | 43.63 | 18.92 | 1.59 | 0.6378/0.4261/0.5282/0.5907/0.6816 |
| Quadratic | 5 | 4661.70 | 0.18 | 12.15 | 0.00 | 0.00 | 45.82 | 42.03 | 0.6056/NaN/NaN/0.3294/0.4275 |
| Cubic | 5 | 4660.53 | 0.46 | 1.20 | 75.90 | 3.78 | 14.94 | 4.18 | 0.8433/0.6743/0.7/0.5044/0.6378 |

No. Latent class: latent class number of the model;

BIC: the Bayesian information Criterion;

The best fitting model is highlighted in bold characters. (NaN: not applicable).

## Table S26. GMM results of neutrophil -to-lymphocyte ratio model fitting process

| Shape of trajectory | No. Latent class | BIC | entropy | Sample size per class (%) | | | | | Mean posterior probabilities |
| --- | --- | --- | --- | --- | --- | --- | --- | --- | --- |
|  |  |  |  | class 1 | class 2 | class 3 | class 4 | class 5 |  |
| Linear | 1 | 1288.51 | 1.00 | 100.00 |  |  |  |  |  |
| Quadratic | 1 | 1294.88 | 1.00 | 100.00 |  |  |  |  |  |
| Cubic | 1 | 1300.65 | 1.00 | 100.00 |  |  |  |  |  |
| Linear | 2 | 1308.28 | 0.00 | 49.93 | 50.07 |  |  |  | 0.5075/0.5075 |
| **Quadratic** | **2** | **1299.87** | **0.77** | **2.48** | **97.52** |  |  |  | **0.746/0.9509** |
| Cubic | 2 | 1309.10 | 0.78 | 1.24 | 98.76 |  |  |  | 0.7754/0.9575 |
| Linear | 3 | 1319.83 | 0.68 | 96.42 | 0.83 | 2.75 |  |  | 0.8882/0.7532/0.7055 |
| Quadratic | 3 | 1323.03 | 0.76 | 3.30 | 95.87 | 0.83 |  |  | 0.7133/0.9259/0.6604 |
| Cubic | 3 | 1330.66 | 0.81 | 0.69 | 98.21 | 1.10 |  |  | 0.7785/0.9442/0.7147 |
| Linear | 4 | 1333.04 | 0.42 | 3.44 | 86.66 | 4.95 | 4.95 |  | 0.7703/0.6873/0.5804/0.7159 |
| Quadratic | 4 | 1340.80 | 0.69 | 5.09 | 1.10 | 92.43 | 1.38 |  | 0.7562/0.7904/0.8647/0.7007 |
| Cubic | 4 | 1355.43 | 0.63 | 1.51 | 92.16 | 4.26 | 2.06 |  | 0.7336/0.8268/0.7104/0.7296 |
| Linear | 5 | 1351.87 | 0.35 | 4.13 | 82.94 | 6.19 | 1.24 | 5.50 | 0.7207/0.5454/0.5331/0.5703/0.6924 |
| Quadratic | 5 | 1358.33 | 0.62 | 0.55 | 1.38 | 85.01 | 11.28 | 1.79 | 0.7718/0.7724/0.789/0.6301/0.738 |
| Cubic | 5 | 1381.31 | 0.41 | 8.80 | 2.06 | 2.06 | 78.82 | 8.25 | 0.6876/0.7036/0.7105/0.6217/0.5795 |

No. Latent class: latent class number of the model;

BIC: the Bayesian information Criterion;

The best fitting model is highlighted in bold characters. (NaN: not applicable).

## Table S27. GMM results of eosinophil -to-lymphocyte ratio model fitting process

| Shape of trajectory | No. Latent class | BIC | entropy | Sample size per class (%) | | | | | Mean posterior probabilities |
| --- | --- | --- | --- | --- | --- | --- | --- | --- | --- |
|  |  |  |  | class 1 | class 2 | class 3 | class 4 | class 5 |  |
| Linear | 1 | 3914.71 | 1.00 | 100.00 |  |  |  |  |  |
| Quadratic | 1 | 3920.65 | 1.00 | 100.00 |  |  |  |  |  |
| Cubic | 1 | 3925.00 | 1.00 | 100.00 |  |  |  |  |  |
| **Linear** | **2** | **3926.60** | **0.46** | **21.74** | **78.26** |  |  |  | **0.7579/0.852** |
| Quadratic | 2 | 3932.41 | 0.29 | 27.88 | 72.12 |  |  |  | 0.7518/0.735 |
| Cubic | 2 | 3940.73 | 0.35 | 10.23 | 89.77 |  |  |  | 0.7235/0.803 |
| Linear | 3 | 3935.75 | 0.61 | 2.30 | 80.82 | 16.88 |  |  | 0.7398/0.8596/0.7011 |
| Quadratic | 3 | 3948.09 | 0.53 | 29.16 | 70.08 | 0.77 |  |  | 0.7376/0.75/0.8062 |
| Cubic | 3 | 3960.71 | 0.40 | 17.90 | 74.17 | 7.93 |  |  | 0.7138/0.7012/0.6658 |
| Linear | 4 | 3951.70 | 0.50 | 2.56 | 17.65 | 64.71 | 15.09 |  | 0.691/0.7276/0.6965/0.674 |
| Quadratic | 4 | 3968.83 | 0.38 | 24.30 | 17.65 | 57.03 | 1.02 |  | 0.6452/0.522/0.5747/0.7095 |
| Cubic | 4 | 3980.34 | 0.31 | 8.44 | 45.01 | 13.81 | 32.74 |  | 0.6585/0.5039/0.609/0.5334 |
| Linear | 5 | 3966.29 | 0.42 | 2.81 | 18.93 | 6.65 | 60.10 | 11.51 | 0.622/0.6635/0.4865/0.5771/0.6571 |
| Quadratic | 5 | 3991.05 | 0.35 | 31.97 | 8.95 | 52.94 | 4.86 | 1.28 | 0.5693/0.4771/0.5119/0.5651/0.6391 |
| Cubic | 5 | 4003.95 | 0.44 | 7.67 | 13.04 | 25.83 | 1.53 | 51.92 | 0.6814/0.673/0.5812/0.751/0.5886 |

No. Latent class: latent class number of the model;

BIC: the Bayesian information Criterion;

The best fitting model is highlighted in bold characters. (NaN: not applicable).

## Table S28. GMM results of platelet -to-lymphocyte ratio model fitting process

| Shape of trajectory | No. Latent class | BIC | entropy | Sample size per class (%) | | | | | Mean posterior probabilities |
| --- | --- | --- | --- | --- | --- | --- | --- | --- | --- |
|  |  |  |  | class 1 | class 2 | class 3 | class 4 | class 5 |  |
| Linear | 1 | 571.57 | 1.00 | 100.00 |  |  |  |  |  |
| Quadratic | 1 | 575.96 | 1.00 | 100.00 |  |  |  |  |  |
| Cubic | 1 | 582.06 | 1.00 | 100.00 |  |  |  |  |  |
| Linear | 2 | 591.34 | 0.00 | 44.98 | 55.02 |  |  |  | 0.5091/0.5075 |
| Quadratic | 2 | 545.64 | 0.37 | 78.13 | 21.87 |  |  |  | 0.8073/0.7497 |
| Cubic | 2 | 557.90 | 0.39 | 78.68 | 21.32 |  |  |  | 0.811/0.7592 |
| Linear | 3 | 567.56 | 0.55 | 13.62 | 82.94 | 3.44 |  |  | 0.685/0.8138/0.744 |
| **Quadratic** | **3** | **544.90** | **0.53** | **9.49** | **82.26** | **8.25** |  |  | **0.7338/0.793/0.8217** |
| Cubic | 3 | 552.13 | 0.47 | 82.12 | 8.94 | 8.94 |  |  | 0.7882/0.6258/0.682 |
| Linear | 4 | 583.59 | 0.60 | 17.19 | 0.96 | 77.58 | 4.26 |  | 0.6995/0.6736/0.7972/0.7138 |
| Quadratic | 4 | 562.88 | 0.45 | 61.49 | 28.47 | 6.19 | 3.85 |  | 0.6352/0.6571/0.8276/0.7744 |
| Cubic | 4 | 551.72 | 0.46 | 38.10 | 52.27 | 3.85 | 5.78 |  | 0.6431/0.6887/0.7172/0.8117 |
| Linear | 5 | 600.95 | 0.43 | 74.28 | 5.36 | 7.02 | 9.35 | 3.99 | 0.6446/0.6164/0.561/0.4764/0.7254 |
| Quadratic | 5 | 565.64 | 0.52 | 59.42 | 29.71 | 6.46 | 3.99 | 0.41 | 0.6503/0.6631/0.8121/0.722/0.7282 |
| Cubic | 5 | 568.75 | 0.51 | 9.77 | 72.21 | 4.95 | 5.50 | 7.57 | 0.6424/0.6931/0.8056/0.6766/0.6419 |

No. Latent class: latent class number of the model;

BIC: the Bayesian information Criterion;

The best fitting model is highlighted in bold characters. (NaN: not applicable).

## Table S29. Logistic-regression results of relationship between hematological parameters trajectories and severity of COVID-19

| **hematological parameters** | **Trajectories** | **Unadjusted model** | | **Model 1** | | **Model 2** | | **Model 3** | |  |
| --- | --- | --- | --- | --- | --- | --- | --- | --- | --- | --- |
|  |  | **OR (95% CI)** | **P** | **OR (95% CI)** | **P** | **OR (95% CI)** | **P** | **OR (95% CI)** | **P** |  |
| Red blood cell count | Decreasing (ref.) |  |  |  |  |  |  |  |  |  |
|  | Increasing | 0.610 (0.294-1.264) | 0.183 | 1.143 (0.488-2.680) | 0.758 | 1.092 (0.449-2.547) | 0.841 | 1.094 (0.463-2.587) | 0.838 |  |
| Red cell distribution width (SD) | Low-increasing (ref.) |  |  |  |  |  |  |  |  |  |
|  | High-increasing | 6.241 (2.170-17.944) | 0.001 | 1.524 (0.418-5.562) | 0.524 | 1.406 (0.337-4.830) | 0.610 | 1.017 (0.261-3.971) | 0.981 |  |
| Red cell distribution width (CV) | Stable (ref.) |  |  |  |  |  |  |  |  |  |
|  | Decreasing | 0.000 (0.000-Inf) | 0.984 | 0.000 (0.000-Inf) | 0.990 | 0.000 (0.000-Inf) | 0.990 | 0.000 (0.000-Inf) | 0.990 |  |
| Mean corpuscular hemoglobin | N-shape (ref.) |  |  |  |  |  |  |  |  |  |
|  | Increasing | 0.670 (0.153-2.938) | 0.595 | 0.495 (0.074-3.300) | 0.467 | 0.433 (0.072-4.108) | 0.407 | 0.431 (0.054-3.438) | 0.427 |  |
| Mean corpuscular volume | N-shape (ref.) |  |  |  |  |  |  |  |  |  |
|  | Increasing | 0.618 (0.140-2.722) | 0.525 | 0.546 (0.085-3.496) | 0.523 | 0.496 (0.087-4.503) | 0.474 | 0.576 (0.080-4.156) | 0.584 |  |
| White blood cell count | U-shape (ref.) |  |  |  |  |  |  |  |  |  |
|  | Decreasing | 2.267 (1.111-4.629) | 0.025 | 1.112 (0.478-2.590) | 0.805 | 1.100 (0.471-2.621) | 0.826 | 1.028 (0.432-2.448) | 0.950 |  |
| Neutrophil count | Inverted N shape (ref.) |  |  |  |  |  |  |  |  |  |
|  | N-shape | 0.366 (0.111-1.214) | 0.100 | 0.503 (0.140-1.811) | 0.293 | 0.508 (0.114-1.609) | 0.301 | 0.549 (0.154-1.959) | 0.356 |  |
| Lymphocyte count | stable (ref.) |  |  |  |  |  |  |  |  |  |
|  | Low-decreasing | 1.136 (0.528-2.444) | 0.744 | 1.629 (0.658-4.031) | 0.291 | 1.664 (0.651-4.135) | 0.275 | 1.451 (0.568-3.707) | 0.437 |  |
|  | U-shape | 3.273 (0.904-11.848) | 0.071 | 7.085 (1.529-32.831) | 0.012 | 6.837 (1.242-29.740) | 0.015 | 6.357 (1.303-31.023) | 0.022 |  |
| Lymphocyte percentage | Decreasing (ref.) |  |  |  |  |  |  |  |  |  |
|  | U-shape | 2.727 (0.778-9.559) | 0.117 | 3.484 (0.836-14.518) | 0.087 | 3.229 (0.635-12.452) | 0.113 | 3.148 (0.717-13.827) | 0.129 |  |
| Basophil count | Stable (ref.) |  |  |  |  |  |  |  |  |  |
|  | Increasing | 0.411 (0.097-1.741) | 0.227 | 0.495 (0.105-2.338) | 0.375 | 0.409 (0.056-1.703) | 0.286 | 0.433 (0.085-2.201) | 0.313 |  |
| Basophil percentage | Stable (ref.) |  |  |  |  |  |  |  |  |  |
|  | Inverted U-shape | 0.492 (0.066-3.692) | 0.491 | 1.254 (0.141-11.175) | 0.839 | 1.166 (0.057-7.477) | 0.893 | 1.374 (0.153-12.318) | 0.776 |  |
| Eosinophil percentage | N-shape (ref.) |  |  |  |  |  |  |  |  |  |
|  | Stable | 0.644 (0.147-2.830) | 0.560 | 0.341 (0.065-1.777) | 0.201 | 0.330 (0.072-2.382) | 0.194 | 0.322 (0.060-1.728) | 0.186 |  |
| Monocyte count | J-shape (ref.) |  |  |  |  |  |  |  |  |  |
|  | Inverted U-shape | 0.605 (0.142-2.581) | 0.497 | 1.451 (0.296-7.111) | 0.646 | 1.320 (0.191-5.557) | 0.735 | 0.926 (0.167-5.128) | 0.930 |  |
| Monocyte percentage | J-shape (ref.) |  |  |  |  |  |  |  |  |  |
|  | Inverted U-shape | 0.507 (0.153-1.685) | 0.268 | 1.088 (0.271-4.372) | 0.905 | 1.124 (0.224-4.046) | 0.871 | 1.124 (0.284-4.446) | 0.867 |  |
| Blood platelet count | U-shape (ref.) |  |  |  |  |  |  |  |  |  |
|  | Inverted U-shape | 0.574 (0.199-1.655) | 0.304 | 1.775 (0.528-5.968) | 0.353 | 1.687 (0.440-5.336) | 0.400 | 1.707 (0.491-5.934) | 0.400 |  |
| Monocyte-to-Lymphocyte Ratio | U-shape (ref.) |  |  |  |  |  |  |  |  | |
|  | Increasing | 0.539 (0.154-1.894) | 0.335 | 0.304 (0.073-1.263) | 0.101 | 0.278 (0.065-1.202) | 0.087 | 0.297 (0.064-1.375) | 0.121 | |
| Neutrophil-to-Lymphocyte Ratio | N-shape (ref.) |  |  |  |  |  |  |  |  | |
|  | Stable | 0.236 (0.065-0.858) | 0.028 | 0.311 (0.054-1.777) | 0.189 | 0.330 (0.057-1.919) | 0.217 | 0.395 (0.064-2.427) | 0.316 | |
| Eosinophil-to-Lymphocyte Ratio | Stable (ref.) |  |  |  |  |  |  |  |  | |
|  | Increasing | 1.508 (0.429-5.302) | 0.522 | 1.465 (0.371-5.785) | 0.586 | 1.514 (0.378-6.061) | 0.558 | 1.486 (0.356-6.197) | 0.586 | |
| Platelet-to-Lymphocyte Ratio | N-shape (ref.) |  |  |  |  |  |  |  |  | |
|  | Stable | 0.709 (0.239-2.101) | 0.535 | 0.373 (0.105-1.318) | 0.126 | 0.373 (0.104-1.337) | 0.130 | 0.388 (0.108-1.397) | 0.147 | |
|  | U-shape | 1.806 (0.484-6.729) | 0.379 | 0.707 (0.162-3.085) | 0.644 | 0.669 (0.148-3.022) | 0.602 | 0.562 (0.121-2.611) | 0.462 | |

Ref. The reference group in the model; Model 1: adjusted for baseline age, gender, and BMI; Model 2: further adjusted for smoking status and drinking habits; Model 3: further adjusted for hypertension and diabetes.

## Table S30 Results of binary logistic analysis testing the association of hematological parameters and severe COVID-19 cases by age group.

| hematological parameters | Patients of all ages (n=998) | | Patients<80 years old (n=986) | |
| --- | --- | --- | --- | --- |
|  | OR (95%CI) | P value | OR (95%CI) | P value |
| Red blood cell count | 0.480 (0.206-1.117) | 0.089 | 0.485 (0.203-1.156) | 0.103 |
| Red cell distribution width (SD) | 1.177 (1.044-1.328) | 0.008 | 1.174 (1.038-1.328) | 0.010 |
| Red cell distribution width (CV) | 1.490 (1.036-2.143) | 0.032 | 1.459 (0.996-2.136) | 0.052 |
| Hematokrit | 0.001 (0.000-23.535) | 0.167 | 0.001 (0.000-66.702) | 0.229 |
| Mean corpuscular hemoglobin concentration | 0.981 (0.943-1.019) | 0.323 | 0.978 (0.939-1.020) | 0.299 |
| Mean corpuscular hemoglobin | 1.031 (0.823-1.292) | 0.789 | 1.047 (0.828-1.324) | 0.702 |
| Mean corpuscular volume | 1.034 (0.955-1.119) | 0.411 | 1.041 (0.959-1.131) | 0.334 |
| Hemoglobin | 0.974 (0.945-1.004) | 0.086 | 0.976 (0.947-1.007) | 0.124 |
| Equation K value of erythrocyte sedimentation rate | 1.012 (0.957-1.071) | 0.669 | 1.016 (0.961-1.073) | 0.582 |
| Erythrocyte sedimentation rate | 1.105 (0.851-1.435) | 0.453 | 1.118 (0.862-1.452) | 0.400 |
| White blood cell count | 0.862 (0.675-1.101) | 0.236 | 0.898 (0.687-1.174) | 0.432 |
| Neutrophil count | 0.877 (0.637-1.209) | 0.425 | 1.005 (0.714-1.413) | 0.978 |
| Neutrophil percent | 1.007 (0.962-1.054) | 0.77 | 1.035 (0.984-1.088) | 0.181 |
| Lymphocyte count | 0.683 (0.371-1.255) | 0.219 | 0.524 (0.261-1.052) | 0.069 |
| Lymphocyte percentage | 0.994 (0.948-1.043) | 0.818 | 0.966 (0.916-1.018) | 0.195 |
| Basophil count | 1.120 (0.162-7.732) | 0.908 | 0.876 (0.122-6.294) | 0.896 |
| Basophil percentage | 1.743 (0.533-5.707) | 0.358 | 1.416 (0.420-4.774) | 0.575 |
| Eosinophil count | 0.180 (0.005-6.954) | 0.358 | 0.166 (0.004-7.680) | 0.359 |
| Eosinophil percentage | 0.942 (0.751-1.181) | 0.604 | 0.934 (0.736-1.184) | 0.571 |
| Monocyte count | 0.245 (0.013-4.770) | 0.353 | 0.364 (0.015-8.598) | 0.531 |
| Monocyte percentage | 1.006 (0.792-1.278) | 0.96 | 1.002 (0.784-1.280) | 0.986 |
| CD3 count | 0.988 (0.969-1.007) | 0.211 | 0.990 (0.977-1.004) | 0.149 |
| CD3 percentage | 0.951 (0.840-1.077) | 0.431 | 1.005 (0.887-1.139) | 0.935 |
| CD4 count | 0.951 (0.879-1.028) | 0.205 | 0.951 (0.879-1.029) | 0.209 |
| CD4 percentage | 0.998 (0.881-1.130) | 0.971 | 1.059 (0.942-1.190) | 0.340 |
| CD8 count | 0.992 (0.978-1.005) | 0.23 | 0.991 (0.977-1.005) | 0.190 |
| CD8 percentage | 0.952 (0.830-1.092) | 0.485 | 0.946 (0.839-1.067) | 0.365 |
| CD4/CD8 ratio | 1.708 (0.761-3.837) | 0.195 | 1.894 (0.829-4.329) | 0.130 |
| Blood platelet count | 0.997 (0.990-1.004) | 0.385 | 0.997 (0.990-1.005) | 0.465 |
| Monocyte-to-Lymphocyte Ratio | 1.223 (0.901-1.659) | 0.197 | 1.311 (0.968-1.774) | 0.080 |
| Neutrophil-to-Lymphocyte Ratio | 1.335 (0.893-1.996) | 0.160 | 1.508 (0.997-2.282) | 0.052 |
| Eosinophil-to-Lymphocyte Ratio | 0.751 (0.003-185.258) | 0.919 | 0.758 (0.003-220.929) | 0.924 |
| Basophil-to-Lymphocytes Ratio | 1.218 (0.883-1.681) | 0.230 | 1.173 (0.841-1.635) | 0.348 |
| Platelet-to-Lymphocyte Ratio | 1.003 (0.994-1.013) | 0.477 | 1.005 (0.996-1.015) | 0.292 |
