# Supplementary material for: Characterization and trajectories of hematological parameters prior to severe COVID-19 based on a large-scale prospective health checkup cohort in western China: a longitudinal study of 13-year follow-up
Source: BMC Med. 2024 Mar 7;22:105. doi: 10.1186/s12916-024-03326-x (PMC10921814; doi:10.1186/s12916-024-03326-x)
Supplement: Supplementary file 3 — Additional file 3: Figures S1-S19. FigS1-S19 Figures of trajectories for hematological parameters. [file 12916_2024_3326_MOESM3_ESM.docx]

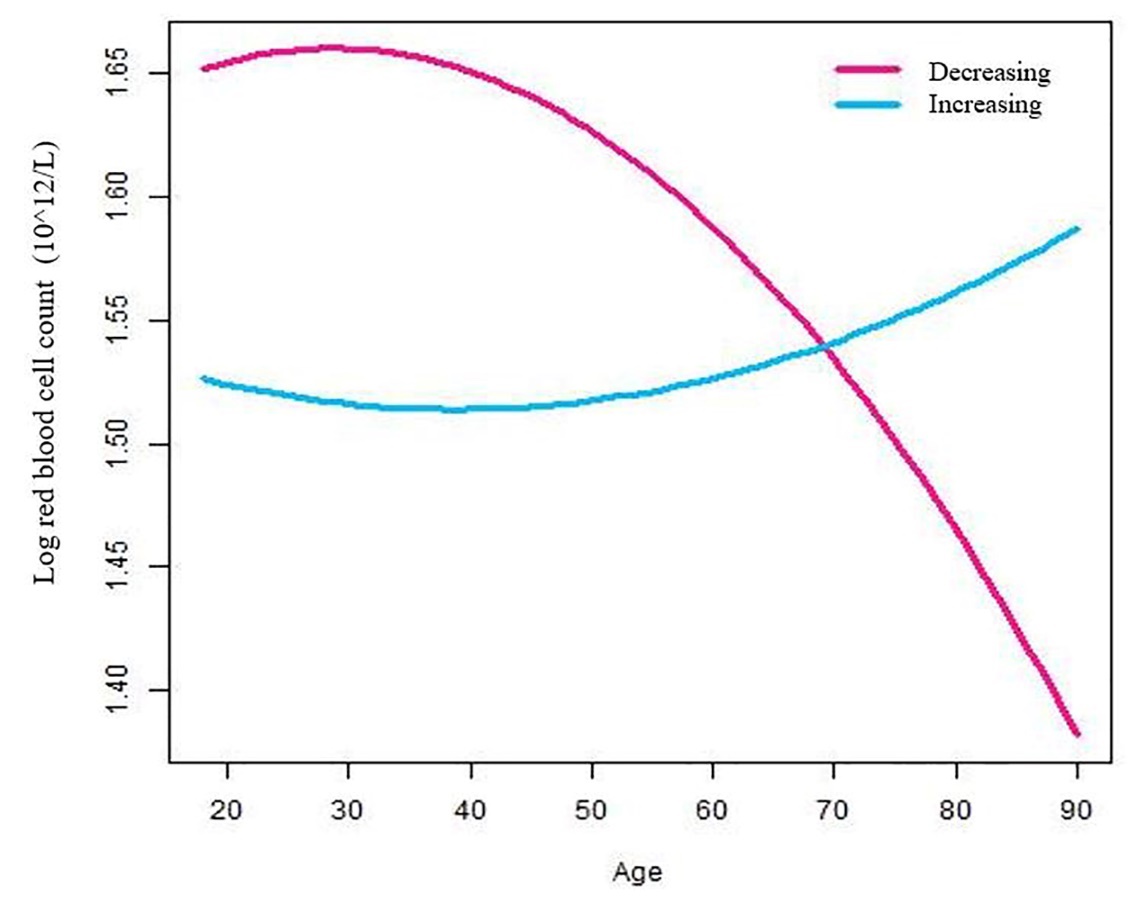


## Fig.S1 Trajectories for red blood cell count


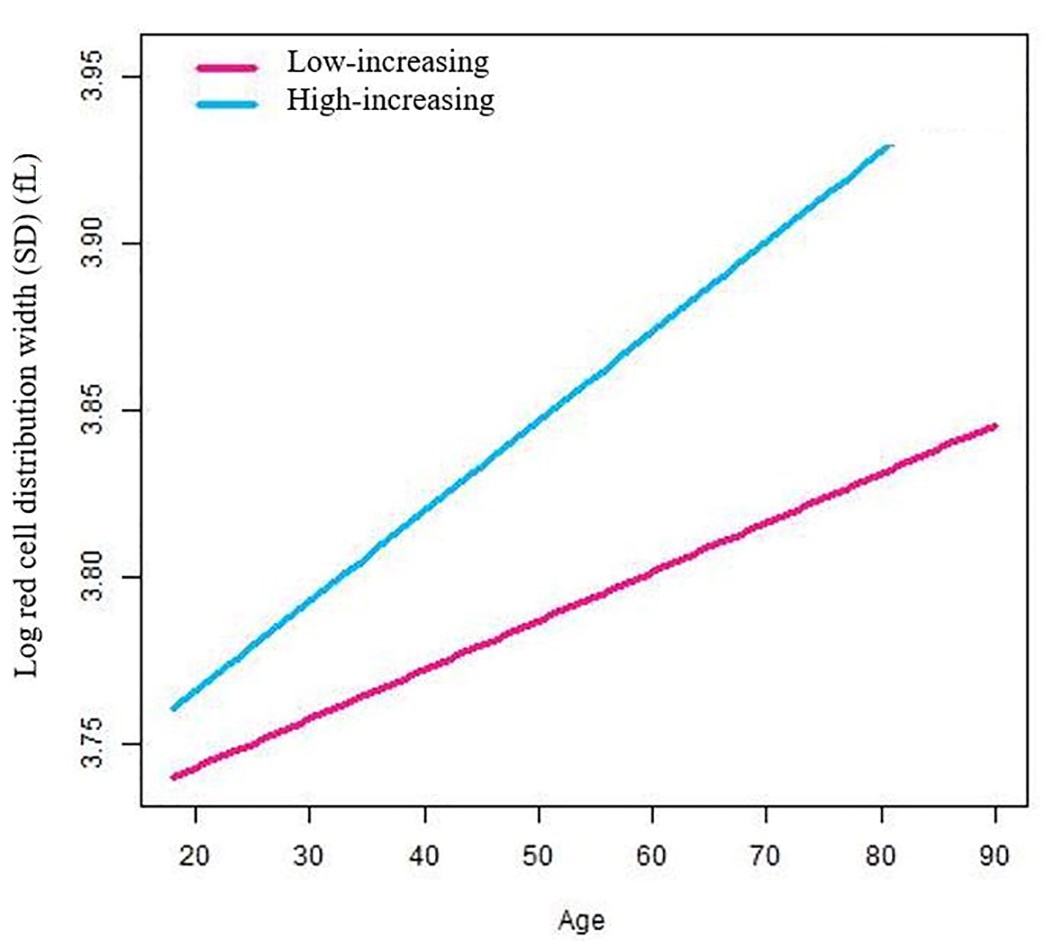


## Fig.S2 Trajectories for red cell distribution width (SD)


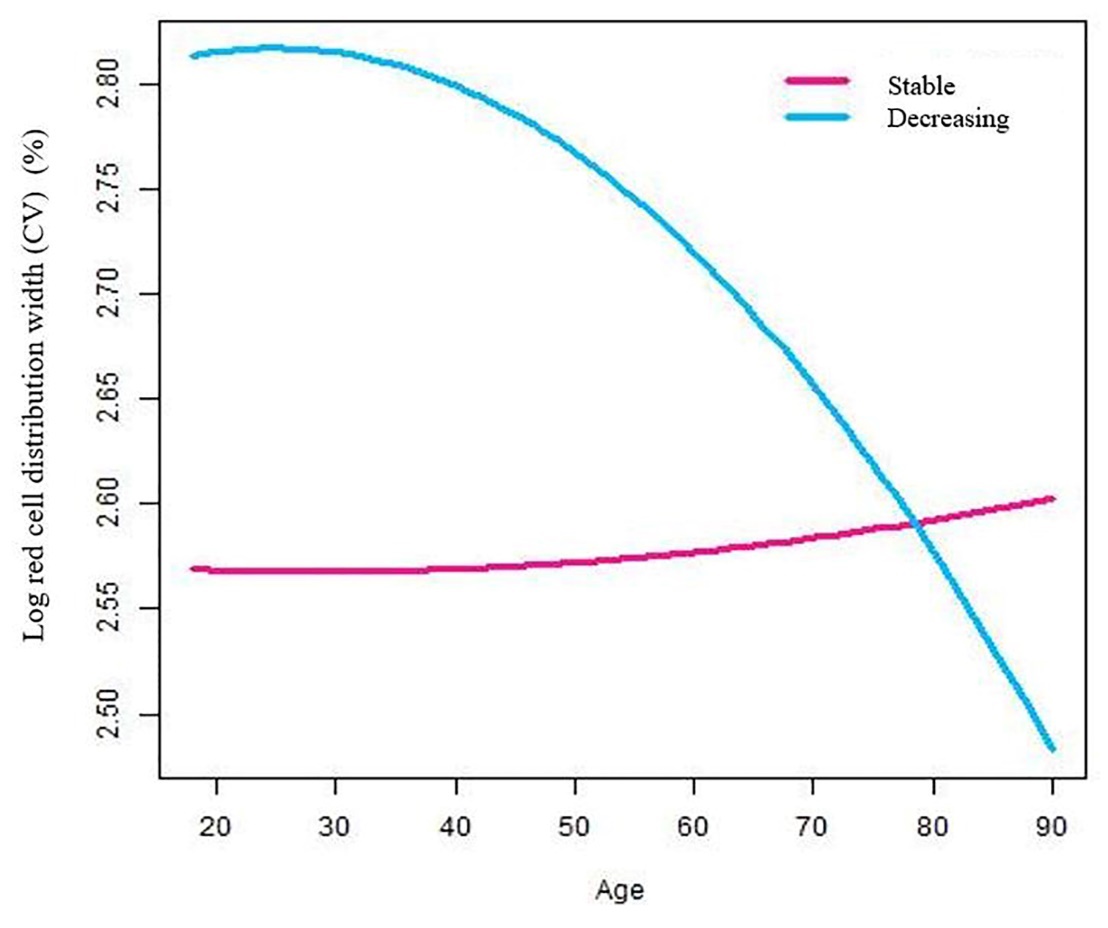


## Fig.S3 Trajectories for red cell distribution width (CV)


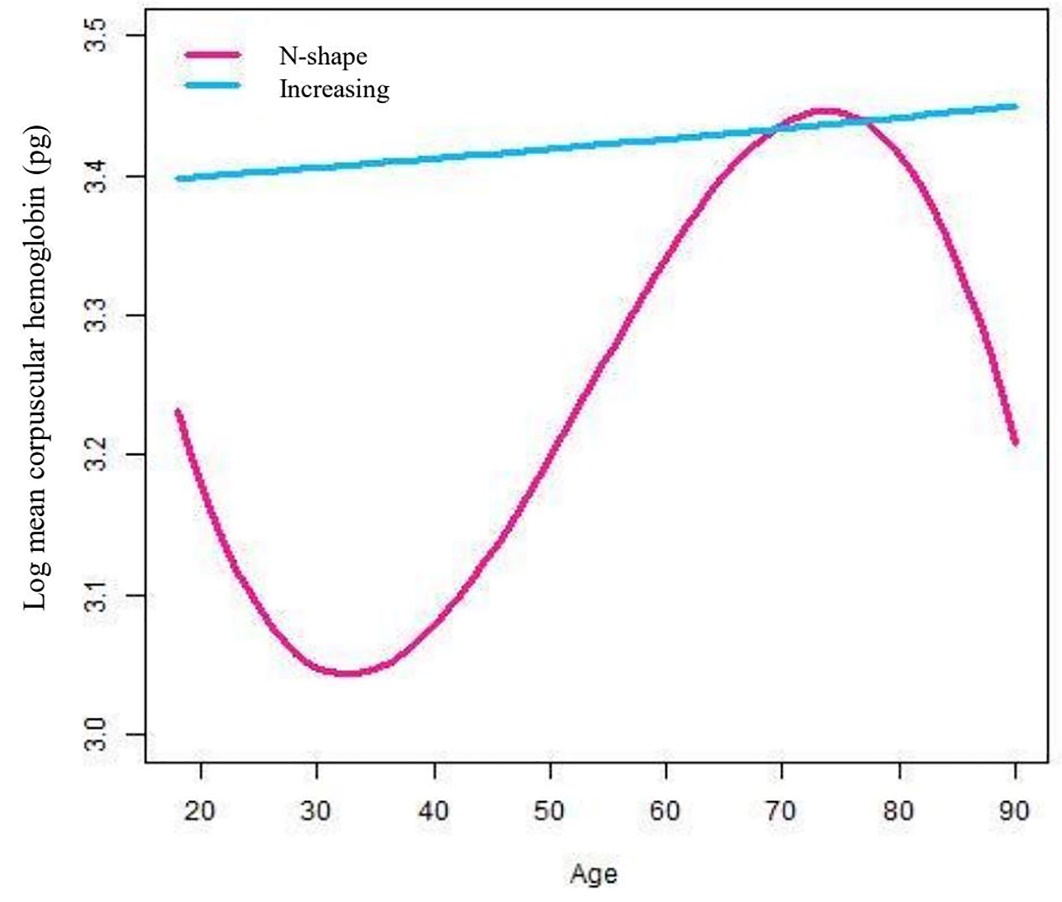


## Fig.S4 Trajectories for mean corpuscular hemoglobin


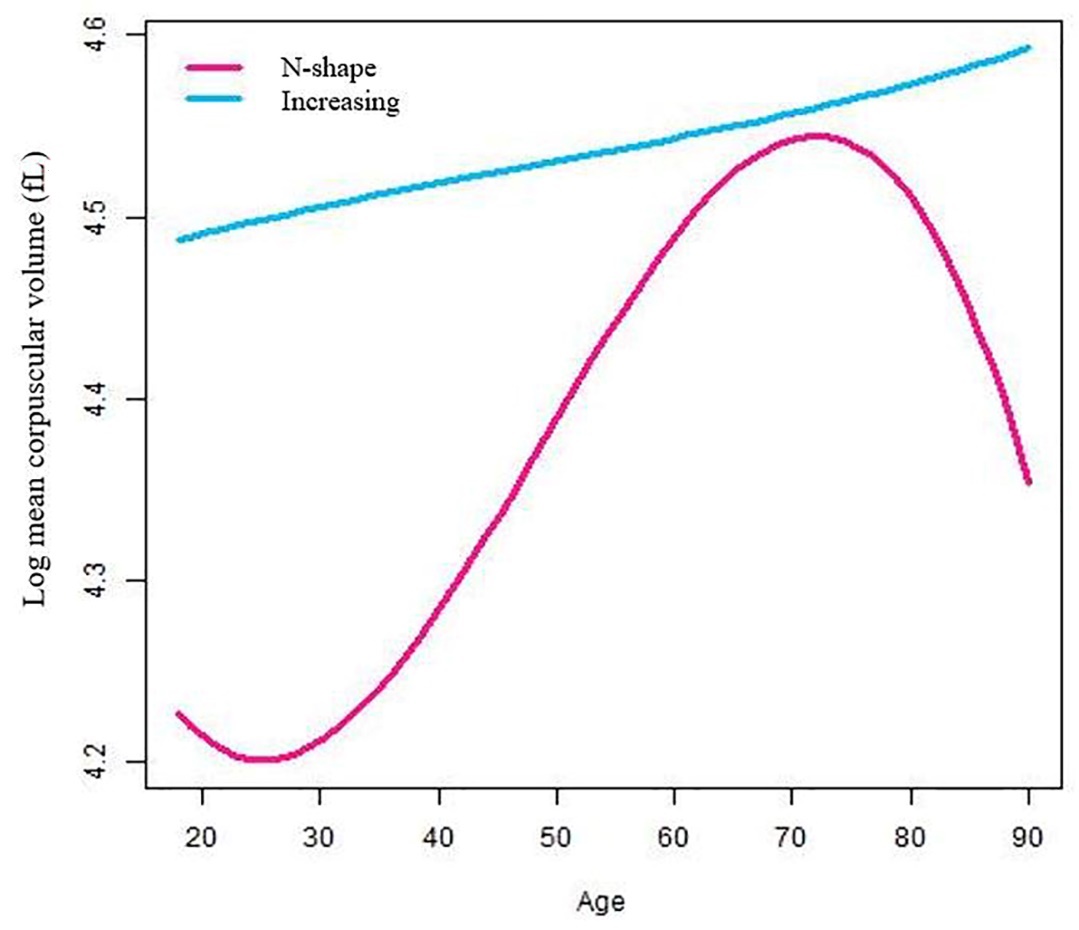


## Fig.S5 Trajectories for mean corpuscular volume


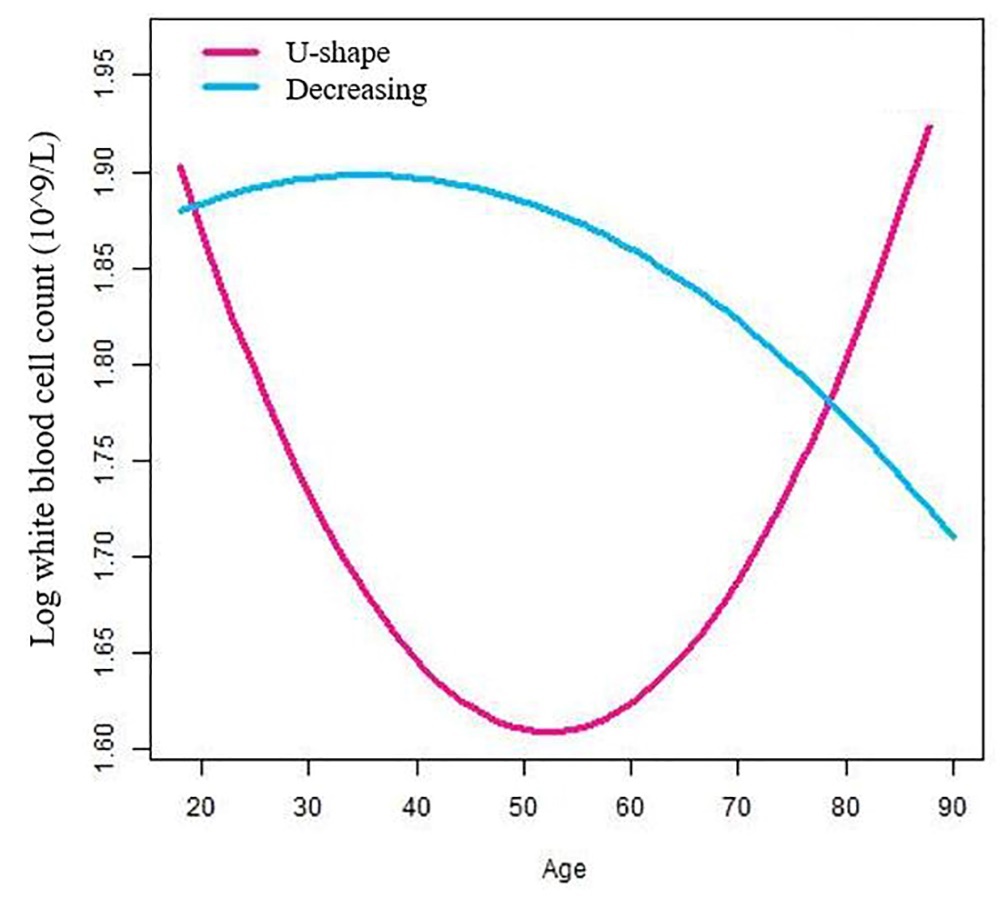


## Fig.S6 Trajectories for white blood cell count


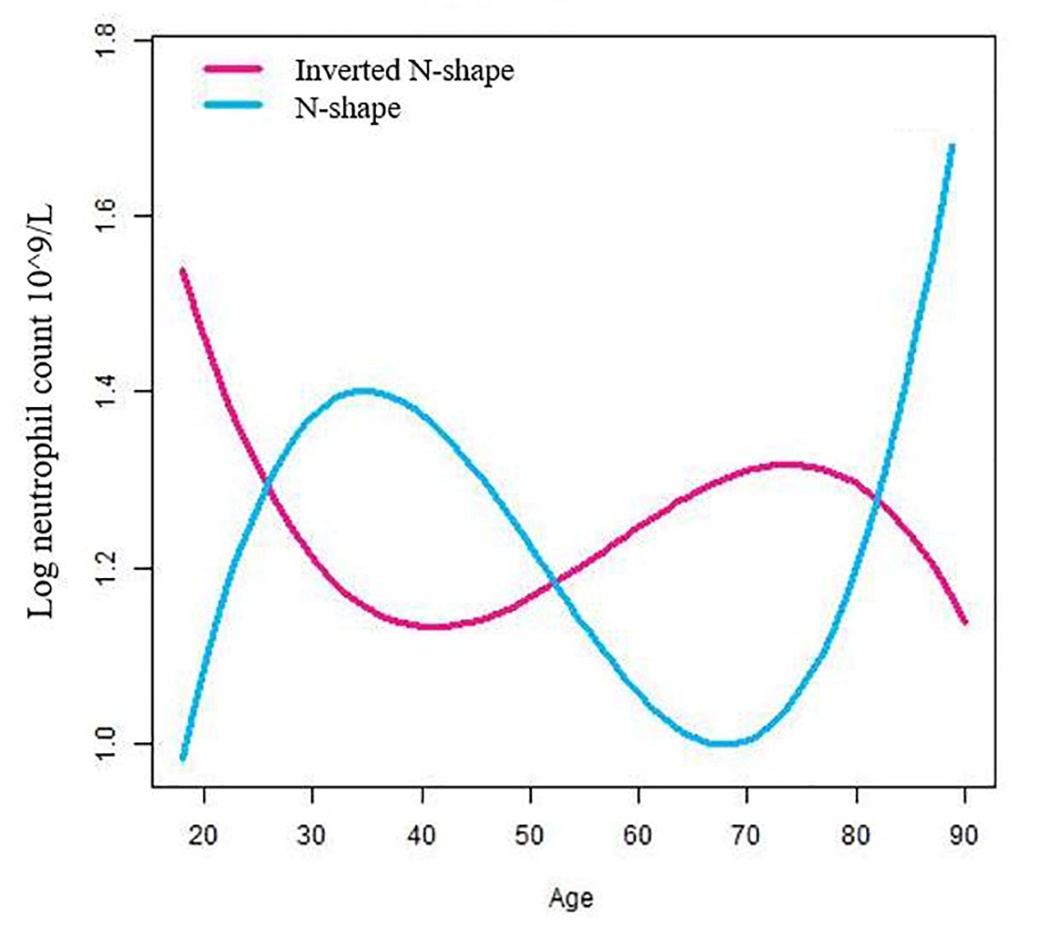


## Fig.S7 Trajectories for neutrophil count


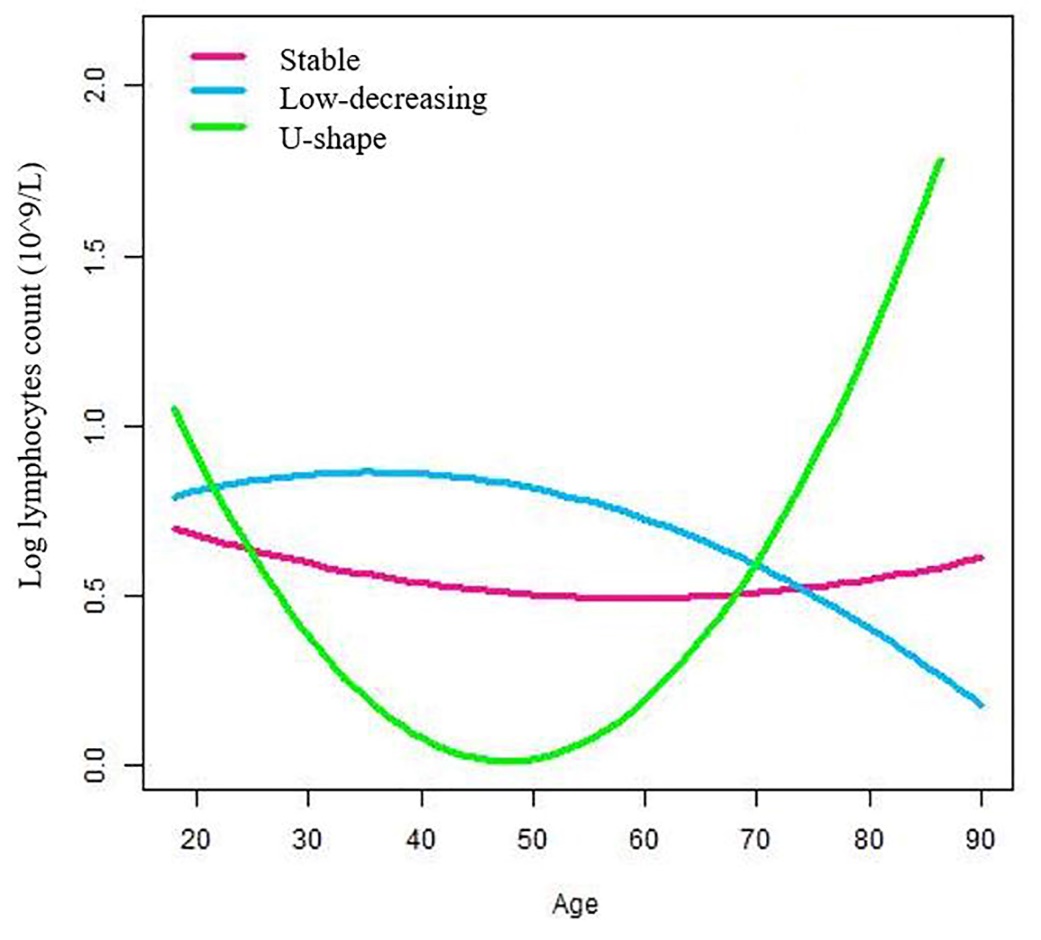


## Fig.S8 Trajectories for Lymphocyte count


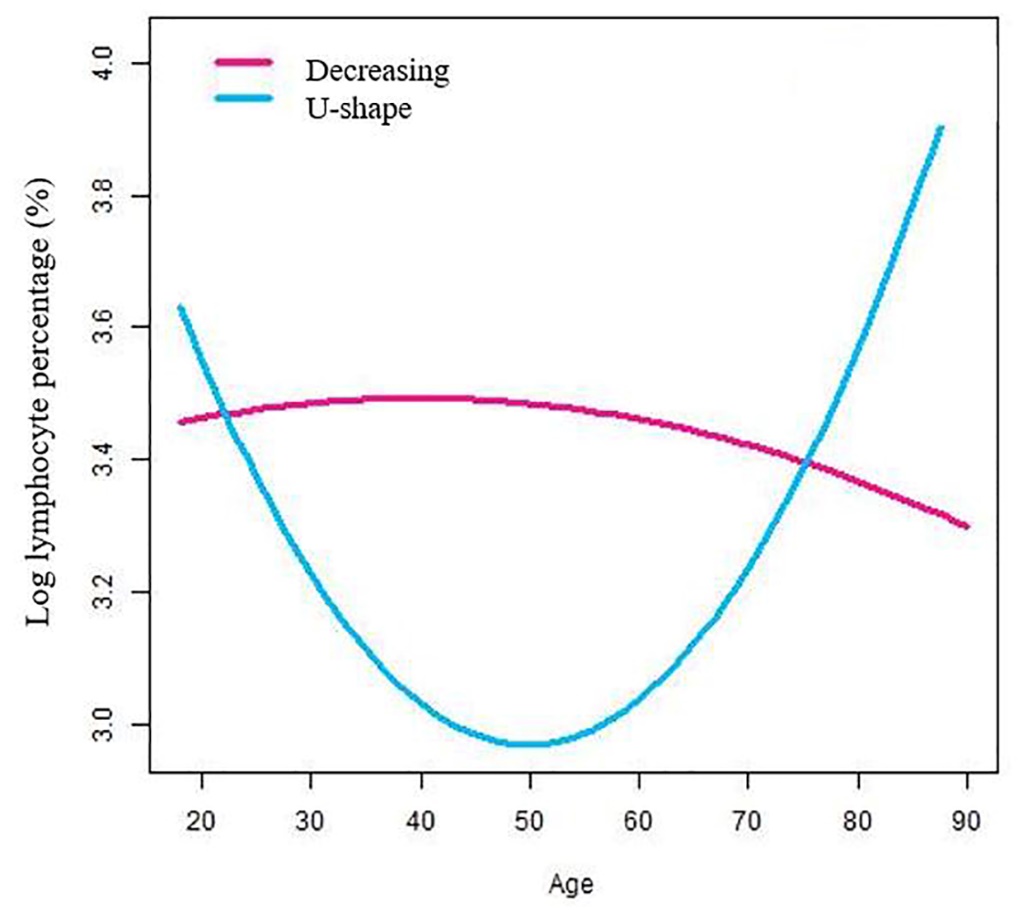


## Fig.S9 Trajectories for lymphocyte percentage


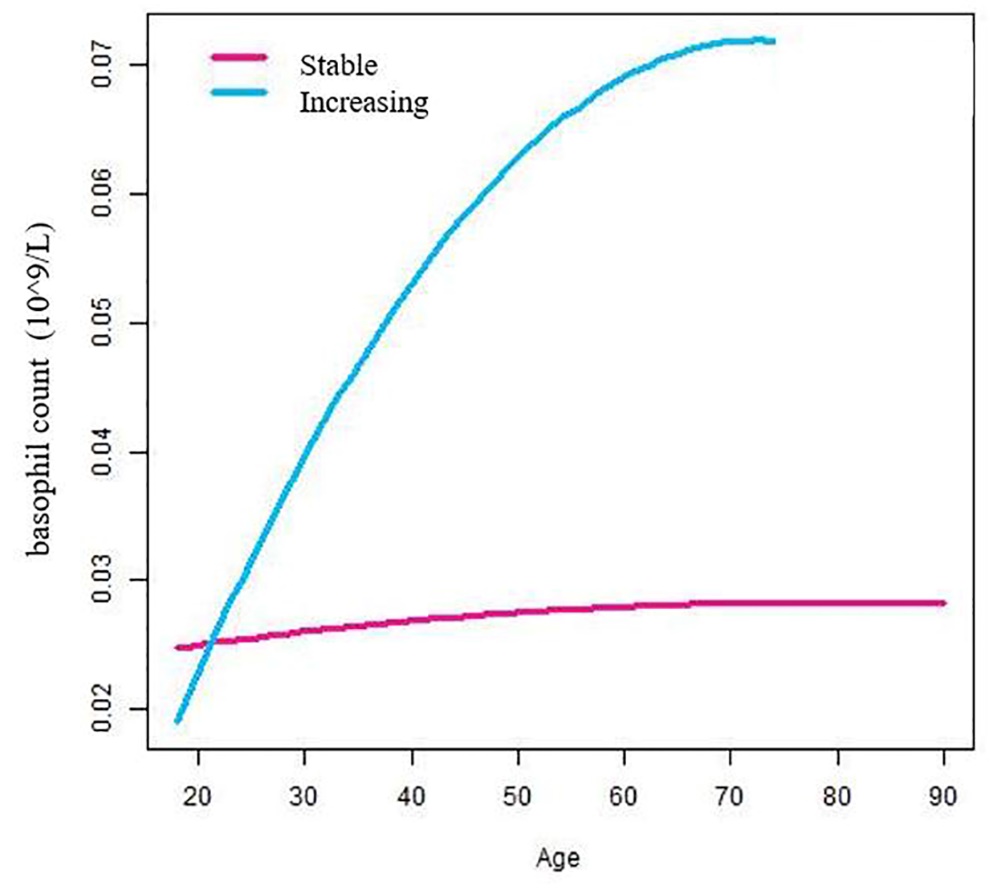


## Fig.S10 Trajectories for basophil count


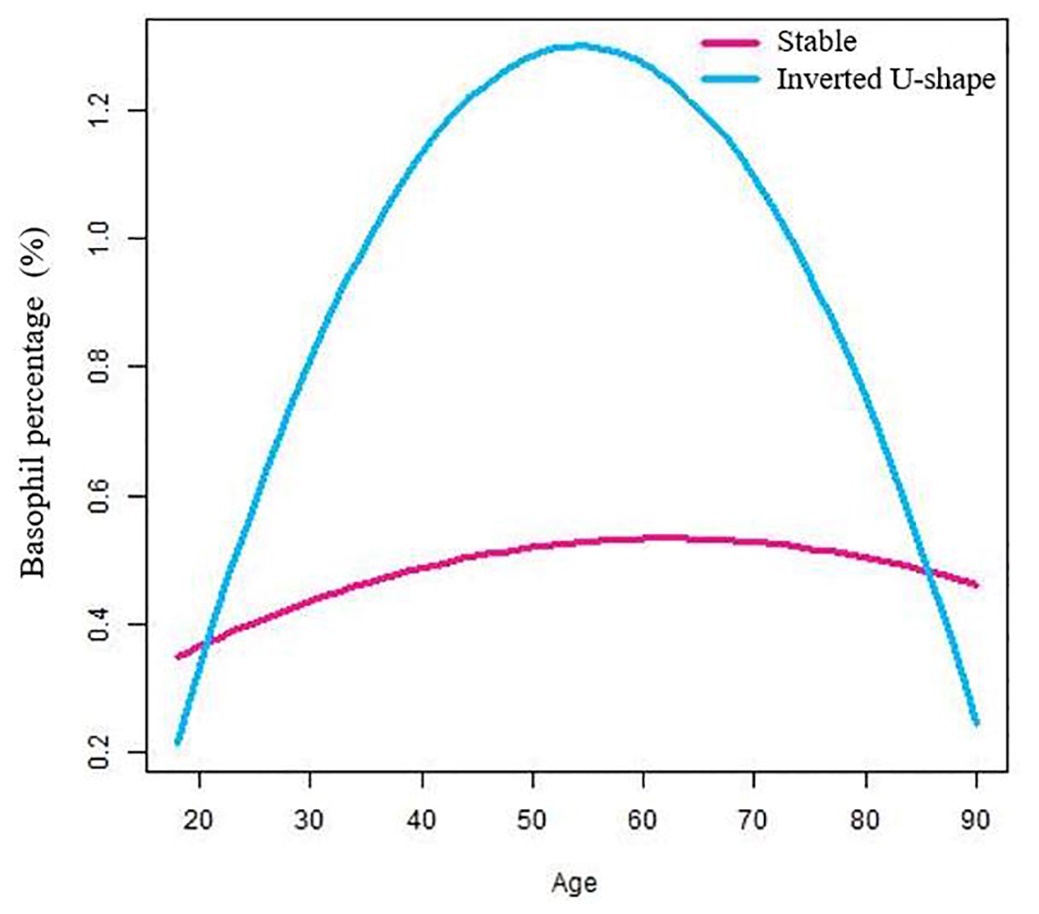


## Fig.S11 Trajectories for basophil percentage


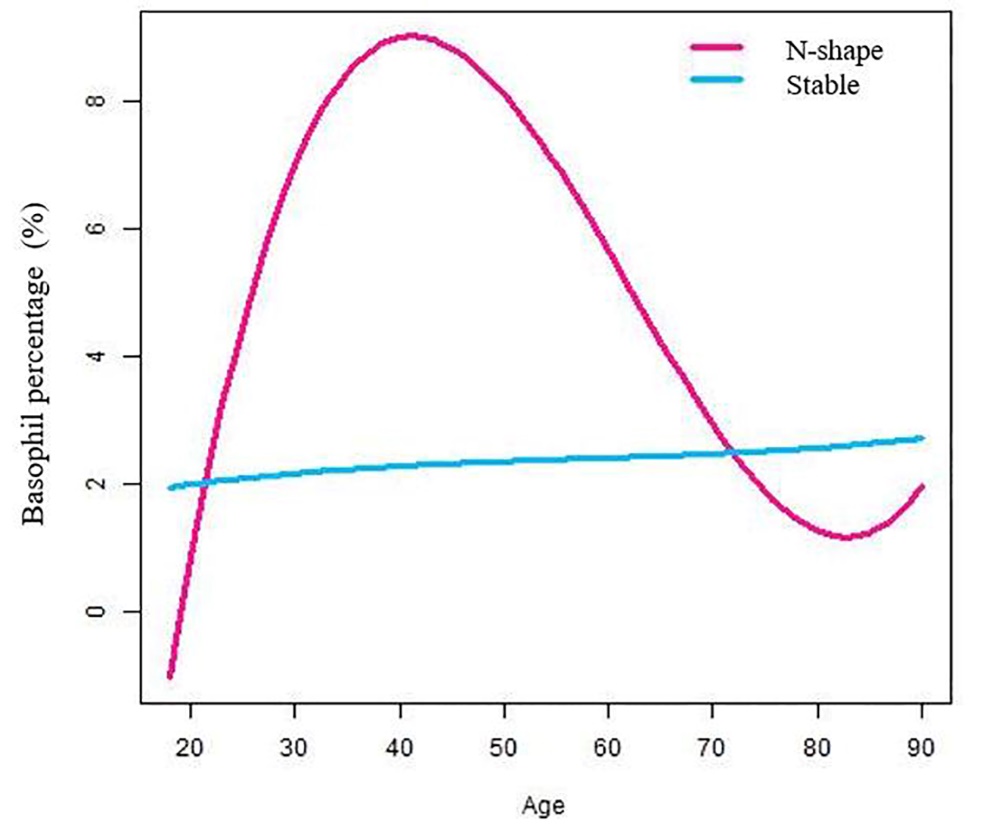


## Fig.S12 Trajectories for eosinophil percentage


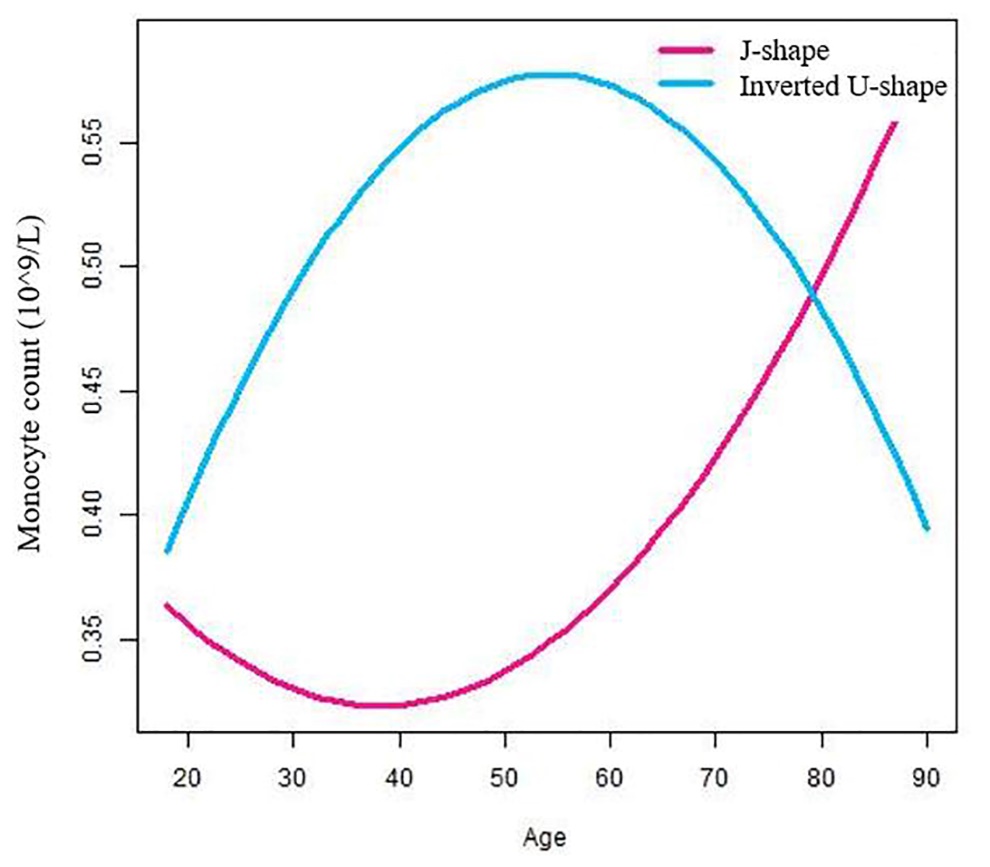


## Fig.S13 Trajectories for monocyte count


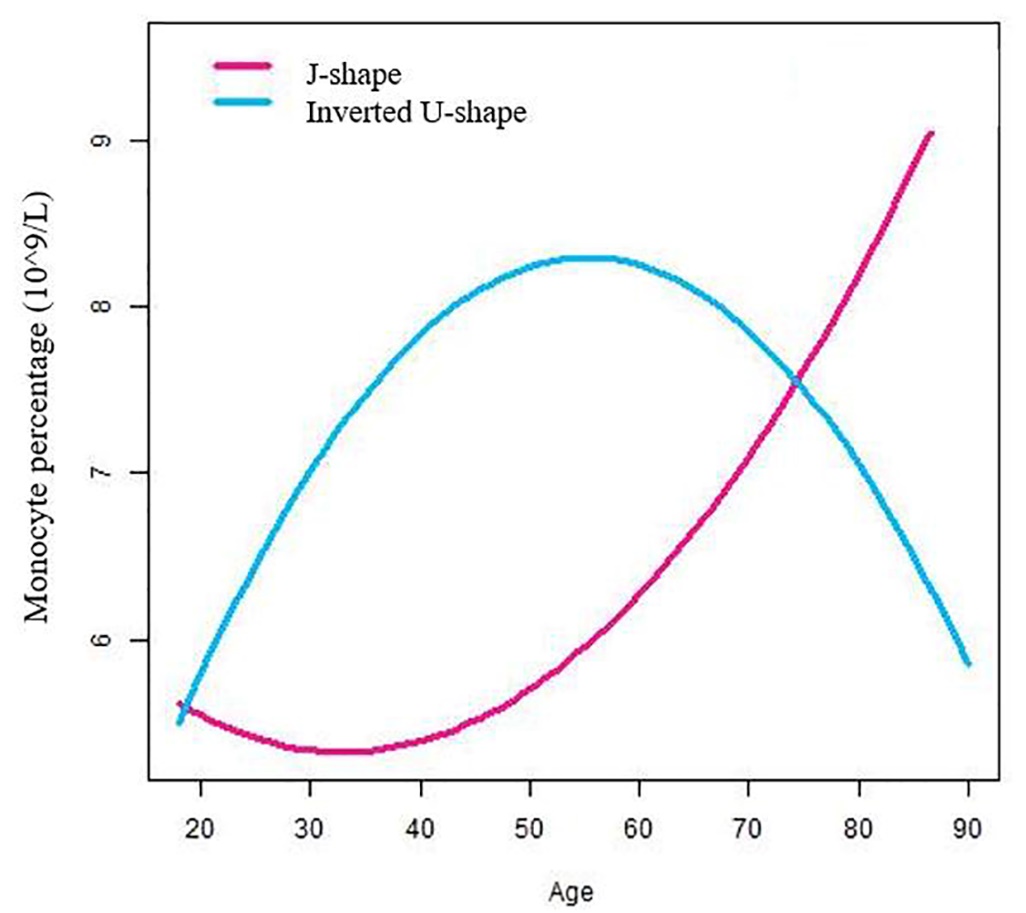


## Fig.S14 Trajectories for monocyte percentage


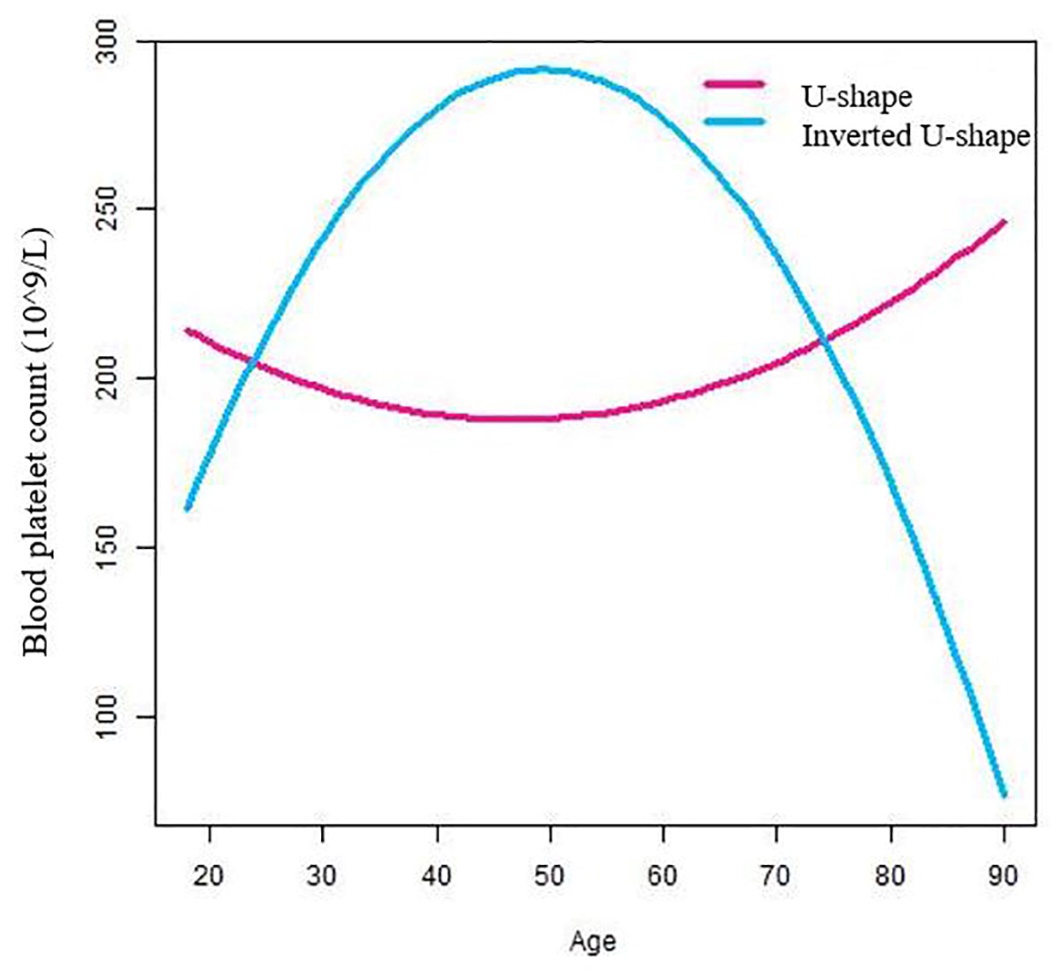


## Fig.S15 Trajectories for blood platelet count


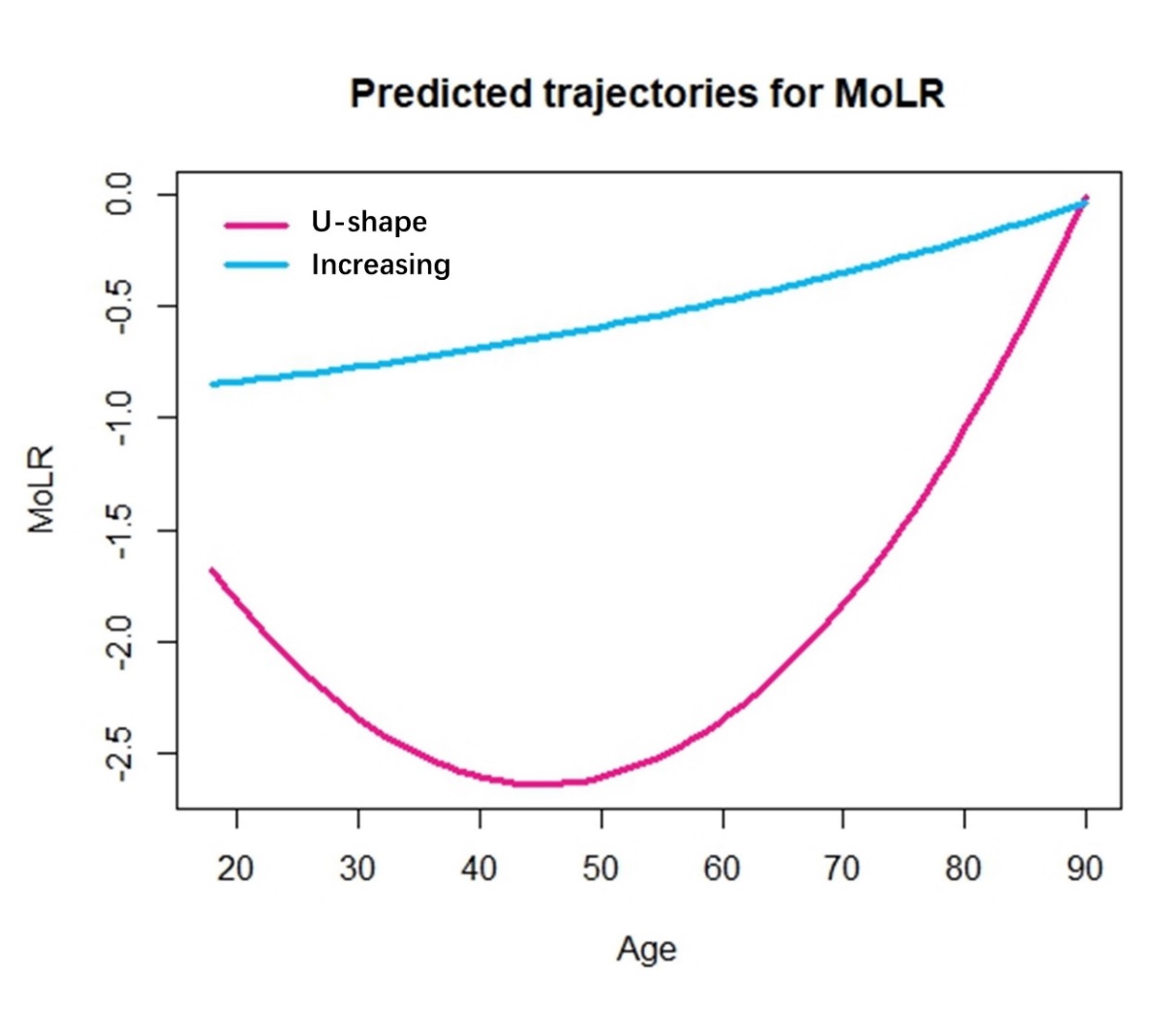


## Fig.S16 Trajectories for monocyte-to-lymphocyte ratio


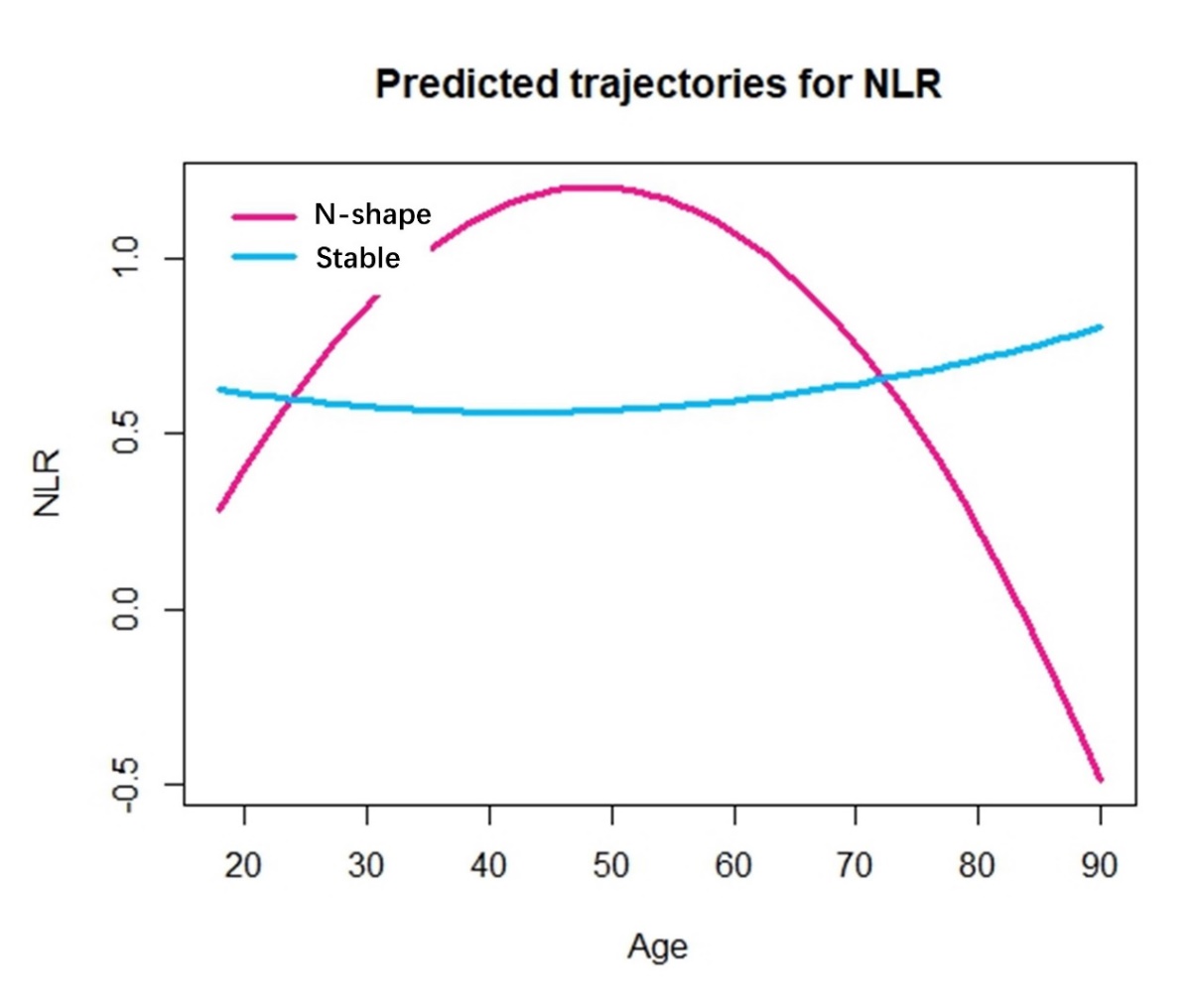


## Fig.S17 Trajectories for neutrophil-to-lymphocyte ratio


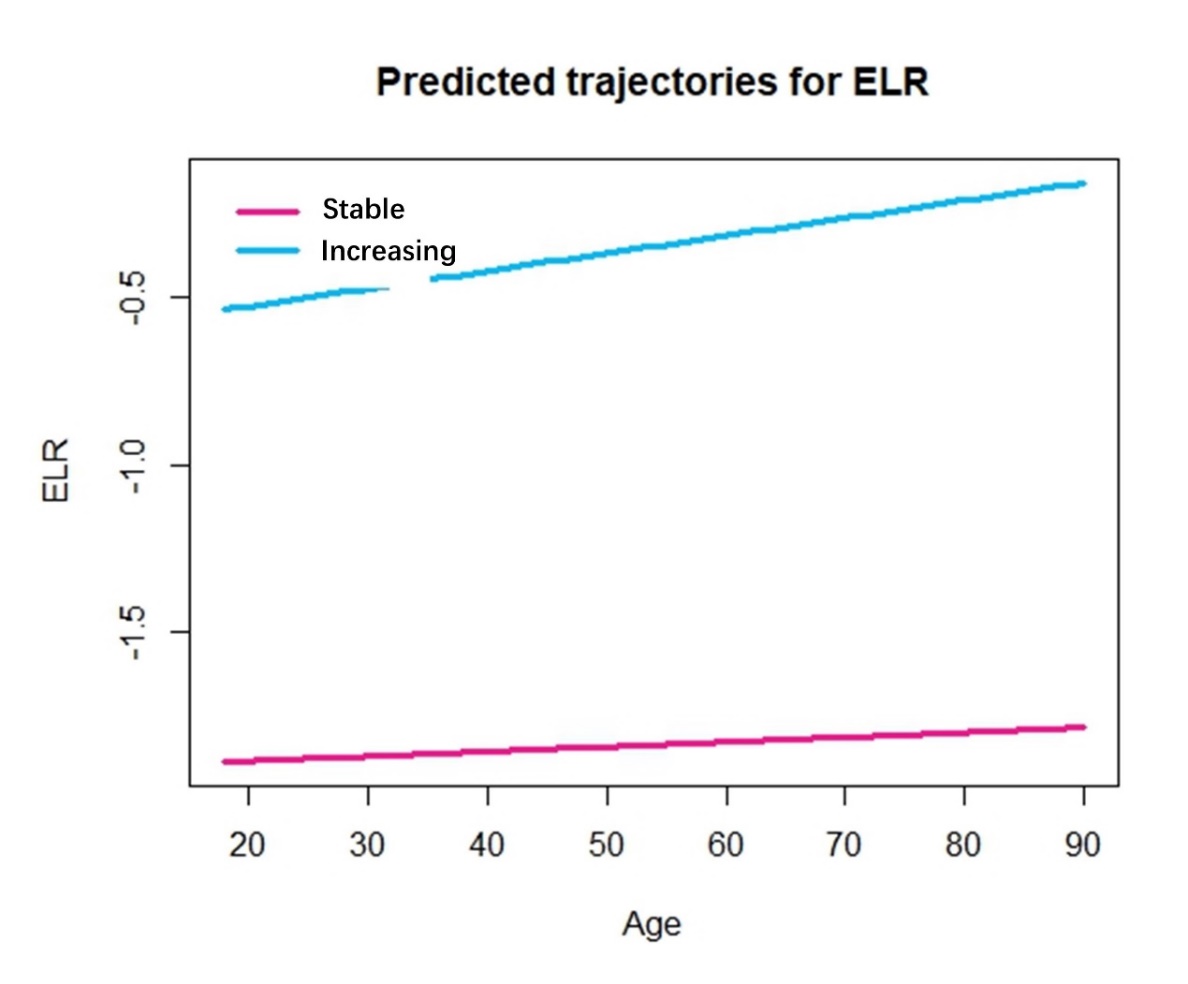


## Fig.S18 Trajectories for eosinophil-to-lymphocyte ratio


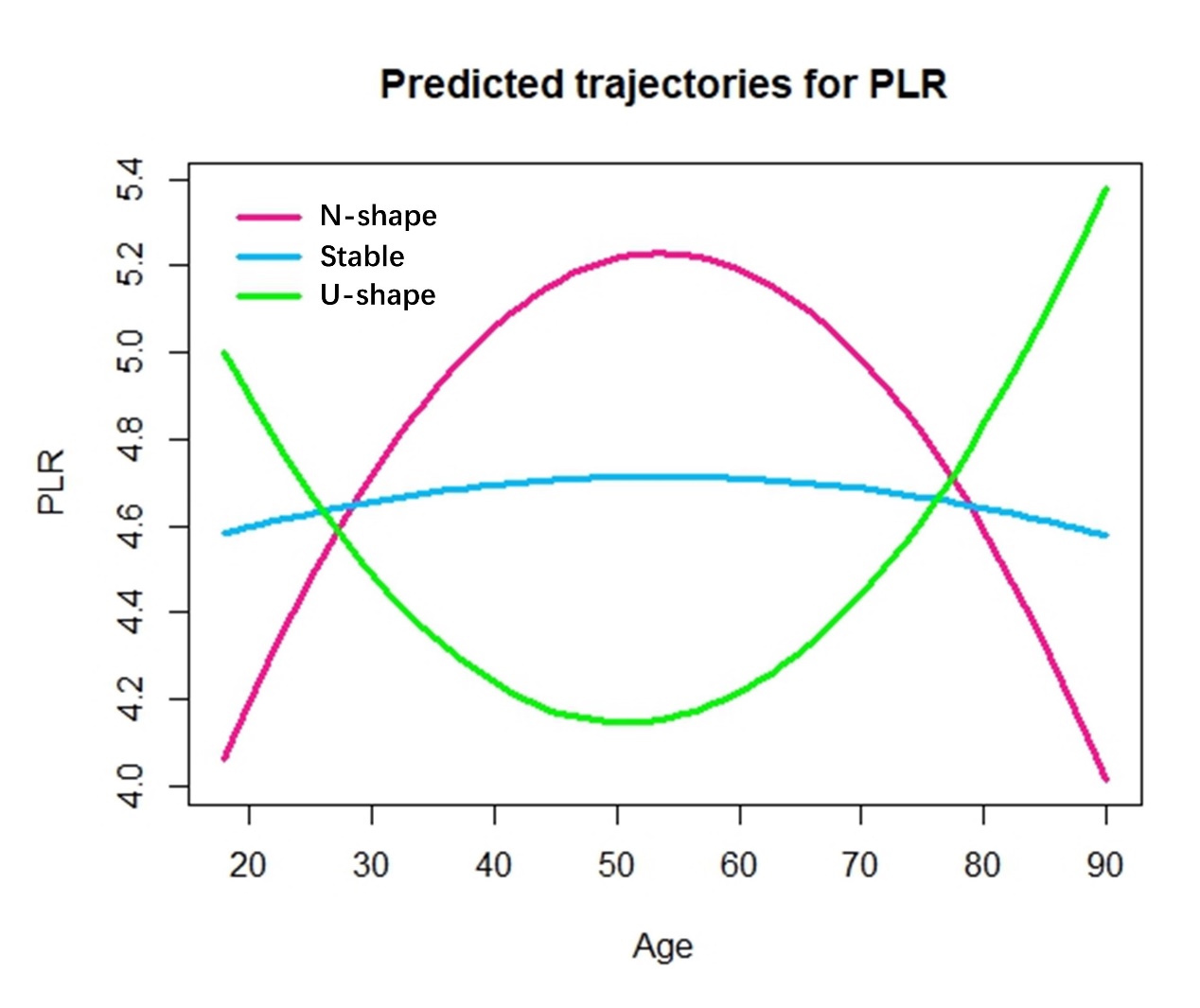


## Fig.S19 Trajectories for platelet-to-lymphocyte ratio
